# Mutagenesis of *FAD2* genes in peanut with CRISPR/Cas9 based gene editing

**DOI:** 10.1186/s12896-019-0516-8

**Published:** 2019-04-29

**Authors:** Mei Yuan, Jun Zhu, Limin Gong, Liangqiong He, Crystal Lee, Suoyi Han, Charles Chen, Guohao He

**Affiliations:** 1 Tuskegee University, Tuskegee, AL 36088 USA; 2 Shandong Peanut Research Institute, Qingdao, 266100 China; 3 Hainan University, Haikou, 570228 China; 4 Guangxi Academy of Agricultural Sciences, Nanning, 530007 China; 5 Henan Academy of Agricultural Sciences, Zhengzhou, 450002 China; 6 Auburn University, Auburn, AL 36849 USA

**Keywords:** Gene-editing, High oleic acid, *ahFAD2* gene, CRISPR/Cas9

## Abstract

**Background:**

Increasing the content of oleic acid in peanut seeds is one of the major goals in peanut breeding due to consumer and industry benefits, such as anti-oxidation and long shelf-life. Homeologous *ahFAD2A and ahFAD2B* genes encode fatty acid desaturases, which are the key enzymes for converting oleic acid to linoleic acid that oxidizes readily. To date, all high oleic acid peanut varieties result from natural mutations occurred in both genes. A method to induce mutations in the genes of other elite cultivars could speed introgression of this valuable trait. The gene-editing approach utilizing CRISPR/Cas9 technology was employed to induce de novo mutations in the *ahFAD2* genes using peanut protoplasts and hairy root cultures as models.

**Results:**

The hot spot of natural mutation in these genes was selected as the target region. Appropriate sgRNAs were designed and cloned into a CRISPR/Cas9 expression plasmid. As a result of CRISPR/Cas9 activity, three mutations were identified - G448A in *ahFAD2A*, and 441_442insA and G451T in *ahFAD2B*. The G448A and 441_442insA mutations are the same as those seen in existing high oleate varieties and the G451T is new mutation. Because natural mutations appear more often in the *ahFAD2A* gene than in the *ahFAD2B* gene in subspecies *A. hypogaea var. hypogaea*, the mutations induced in *ahFAD2B* by gene editing may be useful in developing high oleate lines with many genetic backgrounds after validation of oleic acid content in the transformed lines. The appearance of the G448A mutation in *ahFAD2A* is a further benefit for high oleic acid oil content.

**Conclusions:**

Overall, these results showed that mutations were, for the first time, induced by CRISPR-based gene editing approach in peanut. This research demonstrated the potential application of gene editing for mutagenesis in peanut and suggested that CRISPR/Cas9 technology may be useful in the peanut breeding programs.

**Electronic supplementary material:**

The online version of this article (10.1186/s12896-019-0516-8) contains supplementary material, which is available to authorized users.

## Background

Peanut (*Arachis hypogaea* L.) is an allotetraploid crop of worldwide importance due to its abundant high quality oil production. Natural peanut oil contains two unsaturated fatty acids, oleic acid (36–67%) and linoleic acid (15–43%), that comprise of 80% of the total fatty acid content of peanut seed oil [1]. The quality of oil is dependent on the ratio of these two fatty acids (O/L). High polyunsaturated linoleic acid oil is prone to oxidation, leading to rancidity, off flavors, and short shelf-life; while a high monounsaturated oleic acid oil has 10-fold higher auto-oxidative stability than linoleic acid [2]. In addition, oils containing a high level of oleic acid are nutritionally beneficial for lowering cholesterol [3] and reducing systolic blood pressure [4]. Despite linoleic acid’s adverse impacts on oil stability and its vulnerability to rancid, it is an essential fatty acid for health and nutrition, that cannot be synthesized in humans and must be supplied through diet [5]. Therefore, many efforts have been made in peanut breeding programs to increase the level of oleic acid while keeping a lower level of linoleic acid to improve oil quality and maintain the overall health benefits of peanut oil for the consumer.

The first high-oleate spontaneous mutant line (F435) had about 80% oleic acid and 2% linoleic acid and was identified in 1987 through a peanut germplasm screening project [6]. Since then, several high O/L cultivars have been developed using the mutant line F435 as one of parents in conventional breeding programs [7]. The enzyme, fatty acid desaturase (*FAD2*) catalyzes the conversion of oleic acid to linoleic acid by the addition of a second double bond, generating a polyunsaturated fatty acid from a monounsaturated fatty acid [8]. In peanut, the enzyme is encoded by two homeologous genes, *ahFAD2A* and *ahFAD2B*, located in the A- and B-genomes, respectively, having 99% sequence homology in the coding region with only 11 base pair differences [9–11]. An overall reduction of *FAD2* activity is required to increase the ratio of O/L for accumulation of oleic acid. In addition to natural mutations, various treatments, such as X rays, EMS, gamma rays, and sodium azide, were used to generate mutations in *FAD2* genes to increase oleic acid accumulation, however, these methods generated many other mutations in the genome other than in the target gene [12–15].

Comparison of FAD2 coding sequences from the high oleate (F435) and low oleate (T-90) lines revealed two mutations associated with the high O/L trait [10]. The first mutation in F435 showed a “G” to “A” substitution at 448 bp after the start codon (G448A) in the *ahFAD2A* gene, resulting in a missense amino acid from aspartic acid to asparagine (D150N). The second mutation, an “A” insertion between bp 441 and 442 (441_442insA), in the *ahFAD2B* gene resulted in a frame-shift and generated a premature stop codon [9–11]. In addition, a new high oleate genotype (PI342666) was recently identified through a mass screening effort [16]. The mutation occurred from a single “C” to “G” substitution at 301 bp after the start codon (C301G) in *ahFAD2B* and resulted in an H101D amino acid change. These reports demonstrated that mutations in the coding region of both *ahFAD2A* and *ahFAD2B* genes would alter enzymatic activity to increase the content of high oleic acid in mutant genotypes [7, 9, 10, 17]. To date, there are no reports of the use of an efficient targeted mutagenesis method in peanut that can make gene alterations for desirable traits, such as high oleic acid.

Recently, the RNA-guided Cas9 nucleases from the microbial CRISPR (clustered regularly interspaced short palindromic repeat)-Cas system has emerged as a robust and versatile tool for genome editing in a variety of organisms [18–24]. In an effort to develop novel sources of FAD2 mutants for the high oleate peanut breeding program, we tested the CRISPR/Cas9 method to inactivate both *ahFAD2* genes. Here, we demonstrated high frequency modification of *FAD2* genes by CRISPR/Cas9 that yielded precise mutagenesis of a targeted genomic region in this important crop.

## Results

### Protoplast transfection

CRISPR/Cas9 constructs expressing either sgRNA5 or 6, targeting different areas of the *ahFAD2* genes, were transfected into protoplast isolated from fresh leaves of peanut line 14 AU-01, which contains the G448A mutation in *ahFAD2A* but no mutation in the *ahFAD2B* gene. Purified DNA from protoplasts transfected with the sgRNA6 construct was PCR amplified using FAD2 primer sets (Table 1). Amplicons were cloned and sequenced. Based on 11 A/B SNPs between *ahFAD2A* and *ahFAD2B* genes, 16 *ahFAD2A* sequences and 30 *ahFAD2B* sequences were identified from 46 cloned amplicons. As expected, all 16 *ahFAD2A* sequences contained the G448A mutation. From 30 *ahFAD2B* sequences, surprisingly 18 sequences carried an induced “G” to “T” mutation at position 451 (G451T), the remaining 12 sequences had no mutations, and none of these sequences contained the 441_442insA mutation as seen in some varieties. (Table 2). Cloned PCR products from protoplasts transfected with the sgRNA5 construct did not show evidence of mutation in either *ahFAD2A* or *ahFAD2B*.

**Table 1:** Primers used to amplify genes

Primer name	Forward sequence (5′-3′)	Reverse sequence (5′-3′)
FAD2	GAGGGCGTGTCACTAAGATTG	GGCCATCCTAGTGTGAGTGT
ahFAD2A	GATTACTGATTATTGACTT	CCAACCCAAACCTTTCAGAG
ahFAD2B	CAGAACCATTAGCTTTG	CTCTGACTATGCATCAG
BAR	GCACCATCGTCAACCACTAC	GAAGTCCAGCTGCCAGAAAC
Cas9	GATCGCAAAGTCTGAGCAGG	GATGAATCAGTGTGGCGTCC

**Table 2:** The number of mutations induced from transformed roots and transfected protoplasts by gene editing in the *FAD2* genes

Genotype	Transformed root event	G448A	441_442insA	G451T	No mutation	Total number
Exp27–15,16	10	37^a^	13	28	22	100
GT-C20	10	10				10
14 AU-01	Transfected protoplasts	16^a^		18	12	46
Total reads		63	13	46	34	156

^a^mutation G448A was naturally existed in these two genotypes, not induced by gene editing

### Hairy root transformation

**Fig. 1:**
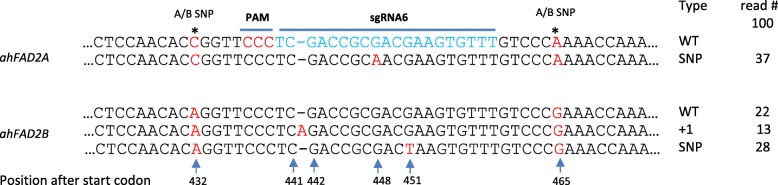
Mutations detected in the targeted region of *ahFAD2A* and *ahFAD2B* genes in the genotype Exp27–1516. In the *ahFAD2A* gene, 37 G448A were pre-existed mutations, while in *ahFAD2B,* 13 441_442insA, 28 G451A mutations, and 22 no mutations were identified

Only the sgRNA6 construct was used for hairy root transformation since the sgRNA5 construct failed in the protoplast assay. Hairy roots were generated from peanut line Exp27–1516, which containing the G448A mutation in the *ahFAD2A* gene and no mutation in the *ahFAD2B* gene, using *Agrobacterium rhizogenes* based transformation then 10 root samples were subjected to PCR amplification using the FAD2 primers listed in the Table 1 [25]. However, direct sequencing of amplicons resulted in a mixture of both *ahFAD2* sequences using FAD2 primers that amplified both genes. Amplicons were separately cloned and up to 10 colonies from each transformation event were sequenced. Among 100 cloned sequences from 10 transformed roots, there were 37 *ahFAD2A* sequences and 63 *ahFAD2B* sequences. All 37 *ahFAD2A* sequences had the expected G448A mutation. Of the 63 *ahFAD2B* sequences, 21% (13/63) displayed mutation 441_442insA, 44% (28/63) had the G451T mutation, and 35% (22/63) were no mutations (Table 2, Fig. 1). Mutations G448A in *ahFAD2A* and 441_442insA in *ahFAD2B* were the same as those observed in line F435. The G451T mutation was new in the *ahFAD2B* gene and it did not occur in the same sequence with mutation 441_442insA. Mutation G451T also led to a pre-mature stop codon.

In a second hairy root transformation experiment, the peanut genotype GT-C20 containing wild type FAD2 genes was transformed with sgRNA6 construct using *A. rhizogenes*. This experiment resulted in 10 transformed roots from which DNAs were extracted and amplified using ahFAD2A [7] and ahFAD2B primers separately. Direct sequencing of PCR products generated unambiguous results so cloning was not necessary. The result showed the mutation G448A mutation in all *ahFAD2A* sequences, but there were no mutations observed in the *ahFAD2B* sequences (Table 2). All mutated sequences are listed in Additional file 1, Additional file 2, and Additional file 3.

## Discussion

Gene editing is a method used to make any targeted genetic change to a chromosome that produces a viable organism. CRISPR/Cas9-based gene editing is the current standard owing to ease of use, high efficiency and success in a wide range of species. We initiated gene editing experiments in the allotetraploid species *Arachis hypogaea*, which contains a large genome (2.8 Gb), to test the suitability of CRISPR/Cas9 technology for future peanut based experiments. Because there is no report of gene editing available in peanut, the protocols developed by [24] in soybean were followed and modified. The well-characterized *ahFAD2* genes were selected as target genes due to their simplicity, known mutant effects, trait value, and scientific interest.

In peanut, the only sources of the high oleate trait are natural mutations in both homeologous *ahFAD2* genes, which leads to high oleic acid content (> 80%), such as observed in the F435 line [6, 9, 10]. While mutation only in the *ahFAD2A* gene results in moderate oleic acid accumulation (50–60%) and no mutations in either genes produces low oleic acid content (40–50%) [26]. After screening the Chinese mini core collection, the study reported that 53.1% of genotypes possessed mutation G448A in the *ahFAD2A* gene and 46.9% with no mutations. Interestingly, the higher frequency (up to 82.8%) of this mutation was existed in *A. hypogaea* subsp. *hypogaea* while the low frequency (15.4%) was observed in *A. hypogaea* subsp. *fastigiat*. However, if there were no mutations occurred in the *ahFAD2A* gene, no mutations were observed in the *ahFAD2B* gene in any lines of the collection. To test if we can induce mutations in the *ahFAD2B* gene, we first chose lines 14 AU-01 and Exp27–1516 for this study since they already contain the G448A mutation in the *ahFAD2A*. Thus, if mutations could be induced in the *ahFAD2B* gene through gene editing then theoretically these genotypes could be converted to high oleate lines. The resulted 441_442insA and the new G451T mutations induced by CRISPR/Cas9-based gene editing in the coding region of *ahFAD2B* may be helpful in breeding programs for the high oleate trait especially since the 441_442insA mutation has been previous characterized and accepted although the actual phenotypic trait still needs to be validated in fully regenerated plants. After validation, mutations in the *ahFAD2B* gene induced by CRISPR/Cas9 based gene editing may provide an alternative way to increase oleic acid content in peanut when the line contains the pre-existing G448A mutation in the *ahFAD2A* gene.

To test if CRISPR/Cas9 based gene editing could also induce a mutation in the *ahFAD2A* gene, the genotype GT-C20, which has no mutations in either *ahFAD2* genes, was then used for editing by the sgRNA6. As a result, mutation G448A was induced in 100% of the *ahFAD2A* gene sequence samples indicating that this is a well-tolerated single point mutation in this coding region. Since no mutation was detected in the *ahFAD2B* gene by gene editing in this genotype, it may be due to a low frequency of mutation in the *ahFAD2B* in this genotype and a higher number of roots may be necessary to be transformed to obtain a result.

Classical breeding relies on the availability of desirable traits in a limited number of natural accessions. Generation of targeted mutations in existing lines would increase genetic diversity and overcome this limitation. In peanut, several approaches have been used in an effort to decrease activity of fatty acid desaturase to allow accumulation of oleic acid in developing seeds. Examples were chemical mutagenesis to induce miniature inverted repeat-transposable element (MITE) insertion in the *ahFAD2* genes [27], and RNAi based silencing of *ahFAD2* genes [28]. A recent study has reported that novel mutations were induced at two locations in the *ahFAD2B* gene through chemical (EMS) and physical (γ rays) mutagenesis. These two induced mutations in the *ahFAD2B* combined with pre-existed mutation in the *ahFAD2A* have resulted in two high-oleic (> 70%) mutant lines [29]. In this study, we used CRISPR/Cas9 gene editing to induce mutagenesis directly at the *ahFAD2* target site. We demonstrated that the same mutations of *ahFAD2* genes that occur in nature could be induced by gene editing using the sgRNA6 construct, which may lead to the high oleate phenotypic trait. Validation of oleic acid content in seeds is difficult due to a challengeable seed production through protoplast and hairy root transformation. Although there were some protocols developed for regeneration by particle bombardment and agrobacterium-mediated methods, and at least 30 genes from other plant species or microorganism were transformed into peanuts, there were still some issues to be concerned, such as genotype-dependance, low frequency of transformation, instability of transformants, and long mandatory procedure with 12–18 months [30, 31]. In our previous experiment for plant regeneration from hairy roots, we only generated calli which hardly produced buds on differentiation medium. A recent study successfully obtained regenerated plants by cotyledonary node transformation [32]. We will use cotyledonary node as explants in gene editing to validate seed phenotype. We also showed that a region targeted by sgRNA5 did not result in measurable mutations suggesting that the sgRNA6 targeted region may be a hot spot of mutagenesis. However, a deletion mutation at the target site of *ahFAD2* was not generated by gene editing even in the hot spot region. Thus, a further study is needed to gain insight into specific mutation that is tolerated in this region.

Application of CRISPR/Cas9 based gene editing is a challenge in polyploidy species due to multiple copies of target genes, particular those with closely related subgenomes, such as in allotetraploid peanut. Studies on subgenome-specific transcriptomes have showed a remarkably low degree of gene loss and functional gene differentiation between subgenomes in some species [33, 34]. [35] reported the effects of gene dosage on oleic acid traits in a panel of peanut accessions and pointed out the relationship between mutations in homeologous *ahFAD2* genes with the ratio of oleic acid to linoleic acid (O/L). The ratio of O/L (> 10) was observed in those genotypes with mutations in both *ahFAD2* genes, the ratio of O/L (2–3) was found in genotypes only having the *ahFAD2A* gene mutated, and the ratio of O/L (~ 1) was seen in genotypes with wild type of both genes. Because their conclusion was drawn on the basis of natural mutations occurring at one location, effects of mutations at different locations in the coding region on the ratio of (O/L) remains unknown. Additionally, combinatorial mutations in different subgenomes may also contribute to oleic acid trait. The effects of gene editing induced mutant allele combinations were recently evaluated at the *FAD2* loci in the hexaploid species *Camelina sativa* [36]. Their report indicated that different mutant allele combinations have resulted in varying content of oleic acid accumulation, ranged from 10 to 62%. But complete *FAD2* loss of function led to important development defects, revealing the importance of polyunsaturated fatty acids in plants. Therefore, multiple sgRNAs designed to target different locations in the coding region of both homeologous *ahFAD2* genes are necessary to study the effects of mutation combinations induced by gene editing on the content of oleic acid and provide unique sources of the high oleate trait for peanut breeding. Moreover, a thorough study of the *ahFAD2* genes is imperative to better understanding *ahFAD2* gene expression, regulation and mechanism that will help improve peanut oil quality.

## Methods

### Plant materials

Three peanut genotypes ‘14 AU-01’, ‘Exp27–1516’ and ‘GT-C20’ were used in this study. The genotype 14 AU-01 is a newly developed high yield breeding line in Auburn University and the Exp27–1516 line has a higher transformation rate compared to other peanut genotypes. These two advanced breeding lines were derived from crosses between ‘AT201 x VIRUGARD’ and ‘GK7-HO X H95’, respectively. The *FAD2* genes of these two genotypes were characterized to ensure they had the expected sequence prior to initiation of experiment. Genomic DNA was extracted from roots and *FAD2* genes were PCR amplified. Amplicons were sequenced and sequencing results confirmed that both genotypes possess the expected G448A *ahFAD2A* mutation as found in line F435. The third genotype GT-C20, a Spanish-type peanut, was kindly provided by Dr. Baozhu Guo (USDA/ARS, Tifton, GA). This genotype was used as plant material because of no mutations detected in either *FAD2A* or *FAD2B* genes after sequence verification of the FAD2 genes.

### Plasmid construction

**Fig. 2:**
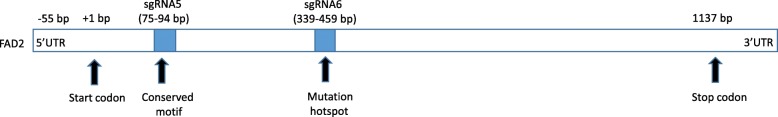
Two sgRNAs, sgRNA5 and sgRNA6, were designed based on the coding region of *FAD2* gene. The sgRNA5 targeted on a conserved motif and the sgRNA6 on the hotspot region

Sequences of *Arachis hypogaea FAD2* genes (accession number: AF272950 and AF272951) were downloaded from the Genbank (https://www.ncbi.nlm.nih.gov/genbank). The open reading frame (ORF) of the *ah*FAD2 genes consists of 1140 bp, encoding 379 amino acids with no introns in the coding sequence [9]. To target and modify the *ahFAD2* genes, two sgRNAs, sgRNA5 (5′-GTTGGCCAACACTGAATGGA-3′) and sgRNA6 (5′-TCGACCGCGACGAAGTGTTT-3′), were designed based on the sequence of coding region using software CRISPR-P 1.0 [37]. Previous reports have demonstrated that natural mutations occur between 441 and 448 bp after the start codon in each gene, which is the area adjacent to the second histidine rich coding region. This region was used as the sgRNA6 target site because it is the hotspot for natural mutations and a PAM motif (GGG) is conveniently located nearby. We speculated that sgRNA6 may cause similar mutations to those seen in natural occurring mutant lines, which may be easier to use for demonstration purposes. The sgRNA5 construct was designed based on the sequences of a conserved motif closed to the 5′ end of the *ahFAD2* genes and was used to test if mutations could be induced at a different location in the *ahFAD2* genes (Fig. 2). These two sgRNAs were synthesized at Eurofins Genomics (Louisville, KY).

Each sgRNA was separately inserted into the p201B-Cas9 binary vector (Addgene, #59177) using ssDNA oligos described by [38]. Briefly, the p201B-Cas9 plasmid was linearized by digestion with two restriction enzymes, *Spel* and *Swal*. A 60-mer oligo was designed to include the sgRNA sequence GN_19_ flanked by 20-nt at both ends that overlap with MtU6 promoter and the Scaffold. The MtU6 promoter and Scaffold DNAs from the pUC gRNA Shuttle (Addgene, # 47024) were amplified using the primers Swal_MtU6F/MtU6R and ScaffordF/Spel_ScaffoldR described by [24]. All four DNAs including linearized vector, MtU6 promoter, scaffold DNA, and sgRNA oligo were combined and incubated at 50^o^ C using NEBuilder® (HiFi DNA Assembly Mix kit, NEB#E5520). The assembled mix was transformed into competent *E. coli* cells (DH5α) and positive clones were identified by sequencing plasmid from single colonies. Plasmids from positive clones were transformed into *Agrobacterium rhizogenes* strain K599 for hairy root transformation. *A. rhizoge*nes strain K599 was kindly provided by Dr. Jianping Wang (University of Florida, USA).

### Protoplasts isolation and transfection

Genotype 14 AU-01 was used for protoplast transfection to test the designed sgRNA constructs. Seeds were sown in 10-cm pots and transferred to the green house after germination. Expanded young leaves from 2 to 3 weeks old plants were collected for protoplast preparation. Protoplast isolation and PEG transfection were performed using the methods described by Xiyan Li (http://www.bio-protocol.org/e70). After 2 days of incubation, protoplasts were collected by centrifugation and protoplast DNA was extracted using the CTAB method [38]. PCR amplification of protoplast DNA was performed using FAD2 primers (Table 1) and amplicons were cloned into the vector in the TOPO TA Cloning kit then sequenced (Invitrogen).

### Hairy root transformation

Since Exp27–1516 showed a higher transformation rate according to our previous experience, we used this genotype for hairy root transformation to test the efficiency of gene editing in peanut. Sterilized Exp27–1516 seeds were germinated on ½ MS liquid medium under sterile conditions and grown for approximately 1 week. The embryo roots and lower hypocotyl were cut from seedlings and the remaining upper portion of each seeding was used as explants for hairy root transformation following the protocol previously described by [24]. Briefly, *A. rhizogenes* was streaked on solid LB with kanamycin (50 mg/L) and grew at 28 °C overnight. *A. rhizogenes* cells were scraped from the plate and resuspended in 6 ml of ½ MS liquid. Explants were dipped in the *A. rhizogenes* solutions and incubated for 20 min with occasional inverting. After incubation, explants were transferred to ½ MS media for co-cultivation in the dark at room temperature for 2 days. After co-cultivation, explants were transferred to ½ MS media supplemented with timentin (300 mg/L) used as *Agrobacterium*-suppressing antibiotics and basta (3.2 mg/L) for selection of transformed tissue containing *bar* gene. Explants were then cultured under fluorescent lights at room temperature with a 16-h photoperiod. After 1.5–2 weeks, transformed roots were harvested from selective media for DNA extraction.

### Mutation detection

Protoplasts or three cm hairy root sections were collected for CTAB DNA isolation [39]. PCR was performed to confirm the presence of the Cas9 and *bar* genes integrated in constructs using specific primers (Table 1). Only those samples that were PCR positive for both genes were used for amplification of *FAD*2 genes from the three genotypes. PCR reactions were carried out in 10 μl aliquots using 1Xbuffer, 20 ng DNA, 0.2 μM of each primer, 0.2 mM dNTPs and 0.25 U Taq Polymerase (Promega). Amplification of *FAD2* genes was performed using specific primer sets: ahFAD2B primers for transformed genotypes 14 AU-01 and Exp27–1516; both ahFAD2A primers [25] and ahFAD2B primers [7] for transformed genotype GT-C20 with wild type DNAs (Table 1). PCR parameters were as follows: 95 °C for 5 min followed by 35 cycles of 95 °C for 30 s, 55 °C for 30 s, and 72 °C for 30 s. PCR products were sequenced directly or cloned and sequenced to detect mutations in each FAD2 gene.

## Additional files

Additional file 1: 46 FAD2 gene sequences isolated from protoplast DNAs transfected with gRNA6 in 14 AU-1. (DOCX 23 kb)

Additional file 2: 100 FAD2 gene sequences isolated from hairy root DNAs transformed with gRNA6 in Exp-1516. (DOCX 14 kb)

Additional file 3: 10 FAD2A gene sequences isolated from hairy root DNAs transformed with gRNA6 in GT-C20. (DOCX 12 kb)

## Abbreviations

CRISPR: clustered regularly interspaced short palindromic repeat
CTAB: Cetyltrimethylammonium bromide
dNTP: deoxyribonucleotide triphosphate
FAD: fatty acid desaturase
MITE: miniature inverted repeat-transposable element
MS medium: Murashige and Skoog medium
