# Supplementary material for: Mutagenesis of FAD2 genes in peanut with CRISPR/Cas9 based gene editing
Source: BMC Biotechnol. 2019 Apr 29;19:24. doi: 10.1186/s12896-019-0516-8 (PMC6489235; doi:10.1186/s12896-019-0516-8)
Supplement: Supplementary file 1 — 46 FAD2 gene sequences isolated from protoplast DNAs transfected with gRNA6 in 14 AU-1. (DOCX 23 kb) [file 12896_2019_516_MOESM1_ESM.docx]

Additional file 1

46 FAD2 gene sequences isolated from protoplast DNAs transfected with gRNA6 in 14AU-1.

>1 fad2-1f

TCACTGAAGCTCTTTCAGGGTTCACATTCAACCCTCCATTCAGTGTTGGCCAACTCAAGA

AAGCAATTCCACCACATTGCTTTGAACGTTCTCTTTTCATATCATTCTCCTATGTTGTCT

ATGATCTCTTAATGGCCTACTTACTCTTCTACATTGCCACCACTTATTTCCACCAGCTTC

CATACCCATTTTCCTTCCTTGCTTGGCCAATCTATTGGGCCATCCAAGGCTGCATTCTCA

CTGGTGTTTGGGTGATTGCTCATGAGTGTGGCCACCATGCCTTCAGCAAGTATCAACTTG

TTGATGACATGGTTGGTTTGATCCTTCACTCTTGTCTATTAGTCCCTTATTTCTCATGGA

AAATCAGCCACCGCCACCTCCACTCCAACACCGGTTCCCTCGACCGCGACTAAGTGTTTG

TCCTGAAACCAAAATCAAAGGTATCACGGTATAACAAGTACATGAACAATCCACTAGAGA

GGGCTATTTCCCTTTTCATCACACTCACACTAGGATGGCCCTGATAATAATGACGTCAGA

ATTCTCGAGTCGGGGAAATGTGCGCGGAACCCCTATTTGTTTATTTTTCTAAATACATTC

AAATATGTATCCGCTCATGAGACAATAACCCTGATAAATGCTTCAATAATATTGAAAAAG

GAAGAGTATGAGTATTCAACATTTCCGTGTCGCCCTTATTCCCTTTTTTGCGGCATTTTG

CCTTCCTGTTTTTGCTCACCCAGAAACGCTGGTGAAAGTAAAAGATGCTGAAGATCAGTT

GGGTGCACGAGTGGGTTACATCGAACTGGATCTCAACAGCGGTAAGATCCTTGAGAGTTT

TCGCCCCGAAGAACGTTTTCCAATGATGAGCACTTTTAAAGTTCTGCTATGTGGCGCGGT

ATTATCCCGTATTGACGCCGGGCAAGAGCAACTCGGTCGCCGCATACACTATTCTCAGAA

TGACTTGGTTGAGTACTCACCAGTCACAGAAAAGCATCCTTACGGATGGCATGACAGTAA

GAGAATTATGCAGTGCTGCCATACCATGAGGTGATACACTGCGGCCAACTTACTTCTGAC

AACGATCGAGGACCGACGGAGCTACCGCTTTTTTGCACACATGGGGGATCATGTACTCGG

CTGATCGTTGGGACGGAGCTTGAATGGAGCATACTAACGACGAGCCGTGAACCCGAGTCC

GTAGCATGGCACACGTTGCGCAACTATTTACTGGCGAACACTACTCATTACTCT

>2 fad2-1f

AGAAAGCTCTTTCAGGGTTCACATTCAAACCCTCCATTCAGTGTTGGCCAACTCAAGAAA

GCAATTCCACCACATTGCTTTGAACGTTCTCTTTTCATATCATTCTCCTATGTTGTCTAT

GATCTCTTAGTGGCCTACTTACTCTTCTACATTGCCACCACTTATTTCCACAAGCTTCCA

TACCCATTTTCCTTCCTTGCTTGGCCAATCTATTGGGCCATCCAAGGCTGCATTCTCACT

GGTGTTTGGGTGATTGCTCATGAGTGTGGCCACCATGCCTTCAGCAAGTACCAACTTGTT

GATGACATGGTTGGTTTGACCCTTCACTCTTGTCTATTAGTTCCTTATTTCTCATGGAAA

ATCAGCCACCGCCGCCACCACTCCAACACCGGTTCCCTCGACCGCAACGAAGTGTTTGTC

CCAAAACCAAAATCAAAGGTATCATGGTATAACAAGTACATGAACAATCCACCAGGGAGG

GCTATCTCCCTCTTCATCACACTCACACTAGGATGGCCCTGATAATAATGACGTCAGAAT

TCTCGAGTCGGGGAAATGTGCGCGGAACCCCTATTTGTTTATTTTTCTAAATACATTCAA

ATATGTATCCGCTCATGAGACAATAACCCTGATAAATGCTTCAATAATATTGAAAAAGGA

AGAGTATGAGTATTCAACATTTCCGTGTCGCCCTTATTCCCTTTTTTGCGGCATTTTGCC

TTCCTGTTTTTGCTCACCCAGAAACGCTGGTGAAAGTAAAAGATGCTGAAGATCAGTTGG

GTGCACGAGTGGGTTACATCGAACTGGATCTCAACAGCGGTAAGATCCTTGAGAGTTTTC

GCCCCGAAGAACGTTTTCCAATGATGAGCACTTTTAAAGTTCTGCTATGTGGCGCGGTAT

TATCCCGTATTGACGCCGGGCAAGAGCAACTCGGTCGCCGCATACACTATTCTCAGAATG

ACTTGGTTGAGTACTCACCAGTCACAGAAAAGCATCTTACGGATGGCATGACAGTAAGAG

AATTATGCAGTGCTGCCATAACCATGAGTGATACACTGCGGCCAACTTACTTCTGACACG

ATCGGAGACGAGGAGCTAACCGCTTTTTGCCACATGGGGGATCATGTTAACTCCGCCTGA

TCGTTGGAAACCGGGAGCTGGATGAGCATACTAACGAGGAGCGTGACCCGATGCTGTAGC

ATGGCCACACGGTTGGCGCAAACTATTATAACCTTGGGCGGCCAACTAACATT

>3 fad2-1f

CTCAGAAGCTCTTTCAGGGTTCACATTCAAACCCTCCATTCAGTGTTGGCCAACTCAAGA

AAGCAATTCCACCACATTGCTTTGAACGTTCTCTTTTCATATCATTCTCCTATGTTGTCT

ATGATCTCTTAGTGGCCTACTTACTCTTCTACATTGCCACCACTTATTTCCACAAGCTTC

CATACCCATTTTCCTTCCTTGCTTGGCCAATCTATTGGGCCATCCAAGGCTGCATTCTCA

CTGGTGTTTGGGTGATTGCTCATGAGTGTGGCCACCATGCCTTCAGCAAGTACCAACTTG

TTGATGACATGGTTGGTTTGACCCTTCACTCTTGTCTATTAGTTCCTTATTTCTCATGGA

AAATCAGCCACCGCCGCCACCACTCCAACACCGGTTCCCTCGACCGCAACGAAGTGTTTG

TCCCAAAACCAAAATCAAAGGTATCATGGTATAACAAGTACATGAACAATCCACCAGGGA

GGGCTATCTCCCTCTTCATCACACTCACACTAGGATGGCCCTGATAATAATGACGTCAGA

ATTCTCGAGTCGGGGAAATGTGCGCGGAACCCCTATTTGTTTATTTTTCTAAATACATTC

AAATATGTATCCGCTCATGAGACAATAACCCTGATAAATGCTTCAATAATATTGAAAAAG

GAAGAGTATGAGTATTCAACATTTCCGTGTCGCCCTTATTCCCTTTTTTGCGGCATTTTG

CCTTCCTGTTTTTGCTCACCCAGAAACGCTGGTGAAAGTAAAAGATGCTGAAGATCAGTT

GGGTGCACGAGTGGGTTACATCGAACTGGATCTCAACAGCGGTAAGATCCTTGAGAGTTT

TCGCCCCGAAGAACGTTTTCCAATGATGAGCACTTTTAAAGTTCTGCTATGTGGCGCGGT

ATTATCCCGTATTGACGCCGGGCAAGAGCAACTCGGTCGCCGCATACACTATTCTCAGAA

TGACTTGGTTGAGTACTCACCAGTCACAGAAAAGCATCTTACGGATGGCATGACAGTAGA

GATTATGCAGTGCTGCCATACCATGAGTGATAACACTGCGCCACTTACTTCTGACACGAT

CGAGGACGAAGGAGCTACCGCTTTTGCACACATGGGGGATCATGTACTCGGCTGATCGTG

GAACGGACTGATGAGCCATACCAACGACGAGGTAACCCGAGCTTAGCATGGCACACGTGG

CCACCTATAACTGGCGAACCATGCTAATCCT

>4 fad2-1f

GCAAAGGCTCTTTCAGGGTTCACATTCAAACCCTCCATTCAGTGTTGGCCAACTCAAGAA

AGCAATTCCACCACATTGCTTTGAACGTTCTCTTTTCATATCATTCTCATATGTTGTCTA

TGATCTCTTAATGGCCTACTTACTCTTCTACATTGCCACCACTTATTTCCACAAGCTTCC

ATACCCATTTTCCTTCCTTGCTTGGCCAATCTATTGGGCCATCCAAGGCTGCATTCTCAC

CGGTGTTTGGGTGATTGCTCATGAGTGTGGCCACCATGCCTTCAGCAAGTACCAACTTGT

TGATGACATGGTTGGTTTGACCCTTCACTCTTGTCTATTAGTTCCTTATTTCTCATGGAA

AATCAGCCACCGCCGCCACCACTCCAACACAGGTTCCCTCGACCGCGACGAAGTGTTTGT

CCCGAAACCAAAATCAAAGGTATCATGGTATAACAAGTACATGAACAATCCACCAGGGAG

GGCTATTTCCCTTTTCATCACACTCACACTAGGATGGCCCTGATAATAATGACGTCAGAA

TTCTCGAGTCGGGGAAATGTGCGCGGAACCCCTATTTGTTTATTTTTCTAAATACATTCA

AATATGTATCCGCTCATGAGACAATAACCCTGATAAATGCTTCAATAATATTGAAAAAGG

AAGAGTATGAGTATTCACATTTCCGTGTCGCCCTTATTCCCTTTTTTGCGCATTTTGCTT

CTGTTTTTGCTCACCCAGAAACGCTGTGAAAGTAAAAGATGCTGAAGATCAGTGGGTGCA

CGAGTGGGCTACATCGACTGATCTCACAGCGTAGATCTGAGAAGTTTCGCCGAAGAACGT

TCATGATGAGCACTTTAAAGTTCTTCTATGTGGGCGCGGATTATCCCGATTGACGCAGGC

AAGAGCATCCGGTGCGCCGCCATACATATTCTCAGAATGAACCTGGTTGAGAATTCCAGA

GTCAGAAGCCTCTCACGATGCTGAACTAAGAATTCGAGGTGTCCTACTTAGGGTTAACCT

TG

>5 fad2-1f

TAAGCAGAGCTCTTTCAGGGTTCACATTCAAACCCTCCATTCAGTGTTGGCCAACTCAAG

AAAGCAATTCCACCACATTGCTTTGAACGTTCTCTTTTCATATCATTCTCCTATGTTGTC

TATGATCTCTTAGTGGCCTACTTACTCTTCTACATTGCCACCACTTATTTCCACAAGCTT

CCATACCCATTTTCCTTCCTTGCTTGGCCAATCTATTGGGCCATCCAAGGCTGCATTCTC

ACTGGTGTTTGGGTGATTGCTCATGAGTGTGGCCACCATGCCTTCAGCAAGTACCAACTT

GTTGATGACATGGTTGGTTTGACCCTTCACTCTTGTCTATTAGTTCCTTATTTCTCATGG

AAAATCAGCCACCGCCGCCACCACTCCAACACCGGTTCCCTCGACCGCAACGAAGTGTTT

GTCCCAAAACCAAAATCAAAGGTATCATGGTATAACAAGTACATGAACAATCCACCAGGG

AGGGCTATCTCCCTCTTCATCACACTCACACTAGGATGGCCCTGATAATAATGACGTCAG

AATTCTCGAGTCGGGGAAATGTGCGCGGAACCCCTATTTGTTTATTTTTCTAAATACATT

CAAATATGTATCCGCTCATGAGACAATAACCCTGATAAATGCTTCAATAATATTGAAAAA

GGAAGAGTATGAGTATTCAACATTTCCGTGTCGCCCTTATTCCCTTTTTTGCGGCATTTT

GCCTTCCTGTTTTTGCTCACCCAGAAACGCTGGTGAAAGTAAAAGATGCTGAAGATCAGT

TGGGTGCACGAGTGGGTTACATCGAACTGGATCTCAACAGCGGTAAGATCCTTGAGAGTT

TTCGCCCCGAAGAACGTTTTCCAATGATGAGCACTTTTAAAGTTCTGCTATGTGGCGCGG

TATTATCCCGTATTGACGCCGGGCAAGAGCAACTCGGTCGCCGCATACACTATTCTCAGA

ATGACTTGGTTGAGTACTCACCAGTCACAGAAAAGCATCTTACGATGGCATGACAGTAGA

GGATTATTGCAGTGCTGCCATACCATGAGTGATACCACTGCGGCCAACTTAACTTCTGAC

ACGATCGGGAGGACTCGAGGAGCTACCGCTTTTTGACACAATGGGGGATCATGGTAACTC

GCCTGGATCGGTGGAAACCGGGACCTGATGAAGCCATATCTAAAGGACGAGCGTGACCCC

GAGTCCTGTAGCAGTGGCACTCACGGTTTTCGCGCCAACCTTTATTACCGTTGGCGTAAT

GCATC

>6 fad2-1f

AGCTAAGCTCATTCAGGGTTCACATTCAACCCTCCATTCAGTGTTGGCCAACTCAAGAAA

GCAATTCCACCACATTGCTTTGAACGTTCTCTTTTCATATCATTCTCCTATGTTGTCTAT

GATCTCTTAGTGGCCTACTTACTCTTCTACATTGCCACCACTTATTTCCACAAGCTTCCA

TACCCATTTTCCTTCCTTGCTTGGCCAATCTATTGGGCCATCCAAGGCTGCATTCTCACT

GGTGTTTGGGTGATTGCTCATGAGTGTGGCCACCATGCCTTCAGCAAGTACCAACTTGTT

GATGACATGGTTGGTTTGACCCTTCACTCTTGTCTATTAGTTCCTTATTTCTCATGGAAA

ATCAGCCACCGCCGCCACCACTCCAACACCGGTTCCCTCGACCGCAACGAAGTGTTTGTC

CCAAAACCAAAATCAAAGGTATCATGGTATAACAAGTACATGAACAATCCACCAGGGAGG

GCTATCTCCCTCTTCATCACACTCACACTAGGATGGCCCTGATAATAATGACGTCAGAAT

TCTCGAGTCGGGGAAATGTGCGCGGAACCCCTATTTGTTTATTTTTCTAAATACATTCAA

ATATGTATCCGCTCATGAGACAATAACCCTGATAAATGCTTCAATAATATTGAAAAAGGA

AGAGTATGAGTATTCAACATTTCCGTGTCGCCCTTATTCCCTTTTTTGCGGCATTTTGCC

TTCCTGTTTTTGCTCACCCAGAAACGCTGGTGAAAGTAAAAGATGCTGAAGATCAGTTGG

GTGCACGAGTGGGTTACATCGAACTGGATCTCAACAGCGGTAAGATCCTTGAGAGTTTTC

GCCCCGAAGAACGTTTTCCAATGATGAGCACTTTTAAAGTTCTGCTATGTGGCGCGGTAT

TATCCCGTATTGACGCCGGGCAAGAGCAACTCGGTCGCCGCATACACTATTCTCAGAATG

ACTTGGTTGAGTACTCACCAGTCACAGAAAGCATCTTACGGATGGCATGACAGTAGAGAA

TTATGCAGTGCTGCCATACCATGAGTGATAACACTGCGGCACTTACTTCTGACACGATCG

GAGAACGAAGGAGCTACCGCTTTTGCCAACATGGGGGATCATGTAACTCGCTGATCGTGG

GAACGGAGCCTGAATGGAAGCATACTCGACGAGGGACCCAGAGCCTTAGCATGGCACACG

TGGCGCCACTATTACGTGGCGCGAACTA

>7 fad2-1f

GCATAGGCTCTTTCATGGTTCACATTCAAACCCTCCATTCAGTGTTGGCCAACTCAAGAA

AGCAATTCCACCACATTGCTTTGAACGTTCTCTTTTCATATCATTCTCCTATGTTGTCTA

TGATCTCTTAGTGGCCTACTTACTCTTCTACATTGCCACCACTTATTTCCACAAGCTTCC

ATACCCATTTTCCTTCCTTGCTTGGCCAATCTATTGGGCCATCCAAGGCTGCATTCTCAC

TGGTGTTTGGGTGATTGCTCATGAGTGTGGCCACCATGCCTTCAGCAAGTACCAACTTGT

TGATGACATGGTTGGTTTGACCCTTCACTCTTGTCTATTAGTTCCTTATTTCTCATGGAA

AATCAGCCACCGCCGCCACCACTCCAACACCGGTTCCCTCGACCGCAACGAAGTGTTTGT

CCCAAAACCAAAATCAAAGGTATCATGGTATAACAAGTACATGAACAATCCACCAGGGAG

GGCTATCTCCCTCTTCATCACACTCACACTAGGATGGCCCTGATAATAATGACGTCAGAA

TTCTCGAGTCGGGGAAATGTGCGCGGAACCCCTATTTGTTTATTTTTCTAAATACATTCA

AATATGTATCCGCTCATGAGACAATAACCCTGATAAATGCTTCAATAATATTGAAAAAGG

AAGAGTATGAGTATTCAACATTTCCGTGTCGCCCTTATTCCCTTTTTTGCGGCATTTTGC

CTTCCTGTTTTTGCTCACCCAGAAACGCTGGTGAAAGTAAAAGATGCTGAAGATCAGTTG

GGTGCACGAGTGGGTTACATCGAACTGGATCTCAACAGCGGTAAGATCCTTGAGAGTTTT

CGCCCCGAAGAACGTTTTCCAATGATGAGCACTTTTAAAGTTCTGCTATGTGGCGCGGTA

TTATCCCGTATTGACGCCCGGGCAAGAGCAACTCGGTCGCCGCATACACTATTCTCAGAA

TGACTTGGGTTGAGTACTCACCAGTCACAGAAAAAGCATCTTACGGATGGCATGACAGTA

AGAGATTATGCAGTGCTGCCATACATGGAGTGATAACACTGGCGGCAAACTTACTTCTGA

CACGATCGAAGGACGGAGGAGCTAACGCTTTTTTGCACACATGGGGATCATGTTAACTCG

CTGGATCGTGGACGAGCTGATGAAGCATACAACGACGAGCGGACCAGATGCGTGAGCAGT

GCACACGTGTGCGCCAACATTACGTGGGCGGATACTC

>8 fad2-1f

GCAAAGCTCTTTCAGGGTTCACATTCAACCCTCCATTCAGTGTTGGCCAACTCAAGAAAG

CAATTCCACCACATTGCTTTGAACGTTCTCTTTTCATATCATTCTCATATGTTGTCTATG

ATCTCTTAATGGCCTACTTACTCTTCTACATTGCCACCACTTATTTCCACAAGCTTCCAT

ACCCATTTTCCTTCCTTGCTTGGCCAATCTATTGGGCCATCCAAGGCTGCATTCTCACCG

GTGTTTGGGTGATTGCTCATGAGTGTGGCCACCATGCCTTCAGCAAGTACCAACTTGTTG

ATGACATGGTTGGTTTGACCCTTCACTCTTGTCTATTAGTTCCTTATTTCTCATGGAAAA

TCAGCCACCGCCGCCACCACTCCAACACAGGTTCCCTCGACCGCGACGAAGTGTTTGTCC

CGAAACCAAAATCAAAGGTATCATGGTATAACAAGTACATGAACAATCCACCAGGGAGGG

CTATTTCCCTTTTCATCACACTCACACTAGGATGGCCCTGATAATAATGACGTCAGAATT

CTCGAGTCGGGGAAATGTGCGCGGAACCCCTATTTGTTTATTTTTCTAAATACATTCAAA

TATGTATCCGCTCATGAGACAATAACCCTGATAAATGCTTCAATAATATTGAAAAAGGAA

GAGTATGAGTATTCAACATTTCCGTGTCGCCCTTATTCCCTTTTTTGCGGCATTTTGCCT

TCCTGTTTTTGCTCACCCAGAAACGCTGGTGAAAGTAAAAGATGCTGAAGATCAGTTGGG

TGCACGAGTGGGTTACATCGAACTGGATCTCAACAGCGGTAAGATCCTTGAGAGTTTTCG

CCCCGAAGAACGTTTTCCAATGATGAGCACTTTTAAAGTTCTGCTATGTGGCGCGGTATT

ATCCCGTATTGACGCCGGGCAAGAGCAACTCGGTCGCCGCATACACTATTCTCAGAATGA

CTTGGGTTGAGTACTCACCAGTCACAGAAAAGCATCTTACGGATGGCATGACAGTAAGAG

ATTATGCAGTGCTGCCATAACCATGAGTGATACACTGCGGCCACTTACTTCTGACACGAT

CGGAGGACCGAAGGAGCTACGCTTTTTTGCACAACATGGGGGATCATGTACTCGCCTGGA

TCGGTGGCACGACTGATGAAGCCTACCACGACGAGCGGGAACCCCGATGCCGTAGCATGG

CACCACGTTGGCCCACATATATACTTGGCAACCACCTTC

>9 fad2-1f

CGCGAAAGCTCTTTCAGGGTTCACATTCAACCCTCCATTCAGTGTTGGCCAACTCAAGAA

AGCAATTCCACCACATTGCTTTGAACGTTCTCTTTTCATATCATTCTCATATGTTGTCTA

TGATCTCTTAATGGCCTACTTACTCTTCTACATTGCCACCACTTATTTCCACAAGCTTCC

ATACCCATTTTCCTTCCTTGCTTGGCCAATCTATTGGGCCATCCAAGGCTGCATTCTCAC

CGGTGTTTGGGTGATTGCTCATGAGTGTGGCCACCATGCCTTCAGCAAGTACCAACTTGT

TGATGACATGGTTGGTTTGACCCTTCACTCTTGTCTATTAGTTCCTTATTTCTCATGGAA

AATCAGCCACCGCCGCCACCACTCCAACACAGGTTCCCTCGACCGCGACGAAGTGTTTGT

CCCGAAACCAAAATCAAAGGTATCATGGTATAACAAGTACATGAACAATCCACCAGGGAG

GGCTATTTCCCTTTTCATCACACTCACACTAGGATGGCCCTGATAATAATGACGTCAGAA

TTCTCGAGTCGGGGAAATGTGCGCGGAACCCCTATTTGTTTATTTTTCTAAATACATTCA

AATATGTATCCGCTCATGAGACAATAACCCTGATAAATGCTTCAATAATATTGAAAAAGG

AAGAGTATGAGTATTCAACATTTCCGTGTCGCCCTTATTCCCTTTTTTGCGGCATTTTGC

CTTCCTGTTTTTGCTCACCCAGAAACGCTGGTGAAAGTAAAAGATGCTGAAGATCAGTTG

GGTGCACGAGTGGGTTACATCGAACTGGATCTCAACAGCGGTAAGATCCTTGAGAGTTTT

CGCCCCGAAGAACGTTTTCCAATGATGAGCACTTTTAAAGTTCTGCTATGTGGCGCGGTA

TTATCCCGTATTGACGCCGGGCAAGAGCAACTCGGTCGCCGCATACACTATTCTCAGAAT

GACTTGGTTGAGTACTCACCAGTCACAGAAAAGCATCTTACGGATGGCATGACAGTAAGA

GATTATGCAGTGCTGCCATACCATGAGTGATACACTGCGGCAACTTACTTCTGACACGAT

CGGAGGACCGAGGAGCTAACGCTTTTTTGCACAACATGGGGGATCATGTACTCGCCTGAT

CGTGGCACGGGCTGAATGAGCATACAACGACGGCGTGAACCCGCAATGCTTAGCATGGCA

CAGCGTGCGCTACCTATACCTGGGCGAACACTACCTAAACTCTCTAGAG

>10 fad2-1f

ACCGAAAGCTCTTTCAGGGTTCACATTCAAACCCTCCATTCAGTGTTGGCCAACTCAAGA

AAGCAATTCCACCACATTGCTTTGAACGTTCTCTTTTCATATCATTCTCCTATGTTGTCT

ATGATCTCTTAGTGGCCTACTTACTCTTCTACATTGCCACCACTTATTTCCACAAGCTTC

CATACCCATTTTCCTTCCTTGCTTGGCCAATCTATTGGGCCATCCAAGGCTGCATTCTCA

CTGGTGTTTGGGTGATTGCTCATGAGTGTGGCCACCATGCCTTCAGCAAGTACCAACTTG

TTGATGACATGGTTGGTTTGACCCTTCACTCTTGTCTATTAGTTCCTTATTTCTCATGGA

AAATCAGCCACCGCCGCCACCACTCCAACACCGGTTCCCTCGACCGCAACGAAGTGTTTG

TCCCAAAACCAAAATCAAAGGTATCATGGTATAACAAGTACATGAACAATCCACCAGGGA

GGGCTATCTCCCTCTTCATCACACTCACACTAGGATGGCCCTGATAATAATGACGTCAGA

ATTCTCGAGTCGGGGAAATGTGCGCGGAACCCCTATTTGTTTATTTTTCTAAATACATTC

AAATATGTATCCGCTCATGAGACAATAACCCTGATAAATGCTTCAATAATATTGAAAAAG

GAAGAGTATGAGTATTCAACATTTCCGTGTCGCCCTTATTCCCTTTTTTGCGGCATTTTG

CCTTCCTGTTTTTGCTCACCCAGAAACGCTGGTGAAAGTAAAAGATGCTGAAGATCAGTT

GGGTGCACGAGTGGGTTACATCGAACTGGATCTCAACAGCGGTAAGATCCTTGAGAGTTT

TCGCCCCGAAGAACGTTTTCCAATGATGAGCACTTTTAAAGTTCTGCTATGTGGCGCGGT

ATTATCCCGTATTGACGCCGGGCAAGAGCAACTCGGTCGCCGCATACACTATTCTCAGAA

TGACTTGGTTGAGTACTCACCAGTCACAGAAAAGCATCTTACGGATGGCATGACAGTAAG

AGAATTATGCAGTGCTGCCATAACCATGGAGTGATACACTGCGGCACTTACTTCTGACAC

GATCGGAGACGAAGGAGCTACGCTTTTTGCACACATGGGGATCCATGGTTACTCGCTTGA

ATCGTGGCACCGGAGCTGGAATGGAGCATACAACGACGAGCGTGACACCGATGCTGTAGC

ATTGACACCACGTGCCCGACATATTACGTGGCGACTTACCATATACTCATCTAC

>11 fad2-1f

CGCGGAGCTCTTTCAGGGTTCACATTCAAACCCTCCATTCAGTGTTGGCCAACTCAAGAA

AGCAATTCCACCACATTGCTTTGAACGTTCTCTTTTCATATCATTCTCATATGTTGTCTA

TGATCTCTTAATGGCCTACTTACTCTTCTACATTGCCACCACTTATTTCCACAAGCTTCC

ATACCCATTTTCCTTCCTTGCTTGGCCAATCTATTGGGCCATCCAAGGCTGCATTCTCAC

CGGTGTTTGGGTGATTGCTCATGAGTGTGGCCACCATGCCTTCAGCAAGTACCAACTTGT

TGATGACATGGTTGGTTTGACCCTTCACTCTTGTCTATTAGTTCCTTATTTCTCATGGAA

AATCAGCCACCGCCGCCACCACTCCAACACAGGTTCCCTCGACCGCGACGAAGTGTTTGT

CCCGAAACCAAAATCAAAGGTATCATGGTATAACAAGTACATGAACAATCCACCAGGGAG

GGCTATTTCCCTTTTCATCACACTCACACTAGGATGGCCCTGATAATAATGACGTCAGAA

TTCTCGAGTCGGGGAAATGTGCGCGGAACCCCTATTTGTTTATTTTTCTAAATACATTCA

AATATGTATCCGCTCATGAGACAATAACCCTGATAAATGCTTCAATAATATTGAAAAAGG

AAGAGTATGAGTATTCAACATTTCCGTGTCGCCCTTATTCCCTTTTTTGCGGCATTTTGC

CTTCCTGTTTTTGCTCACCCAGAAACGCTGGTGAAAGTAAAAGATGCTGAAGATCAGTTG

GGTGCACGAGTGGGTTACATCGAACTGGATCTCAACAGCGGTAAGATCCTTGAGAGTTTT

CGCCCCGAAGAACGTTTTCCAATGATGAGCACTTTTAAAGTTCTGCTATGTGGCGCGGTA

TTATCCCGTATTGACGCCCGGGCAGAGCAACTCGGTCGCCGCATACACTATTCTCAGAAT

GACTTGGTTTGAGTACTCACCAGTCACAGAAAAGCATCTTACGGATGGCATGACAGTAAG

AGATTATGCAGTGCTGCATAACCATGAGTGATACACTGCGGCACTACTTCTGACACGATC

GGAGGACCGAAGGAGCTACCGCTTTTTGCCCAACATGGGGGATCATGTTACTTCGCTTGG

ATCGGTGGCACGGGAGCTGATTGAGGCATCAACGACGAGCGTGACCCCAGATGCTGTAGC

ATTGGCCACACGTGTGGCAACATACTATACCTGTGGCGAACTACTAACTCA

>12 fad2-1f

GGCGGAGCCTCTTTCAGGTTCACATTCAAACCCTCCATTCAGTGTTGGCCAACTCAAGAA

AGCAATTCCACCACATTGCTTTGAACGTTCTCTTTTCATATCATTCTCCTATGTTGTCTA

TGATCTCTTAATGGCCTACTTACTCTTCTACATTGCCACCACTTATTTCCACCAGCTTCC

ATACCCATTTTCCTTCCTTGCTTGGCCAATCTATTGGGCCATCCAAGGCTACATTCTCAC

TGGTGTTTGGGTGATTGCTCATGAGTGTGGCCACCATGCCTTCAGCAAGTATCAACTTGT

TGATGACATGGTTGGTTTGATCCTTCACTCTTGTCTATTAGTCCCTTATTTCTCATGGAA

AATCAGCCACCGCCACCTCCACTCCAACACCGGTTCCCTCGACCGCGACTAAGTGTTTGT

CCTGAAACCAAAATCAAAGGTATCACGGTATAACAAGTACATGAACAATCCACTAGAGAG

GGCTATTTCCCTTTTCATCACACTCACACTAGGATGGCCCTGATAATAATGACGTCAGAA

TTCTCGAGTCGGGGAAATGTGCGCGGAACCCCTATTTGTTTATTTTTCTAAATACATTCA

AATATGTATCCGCTCATGAGACAATAACCCTGATAAATGCTTCAATAATATTGAAAAAGG

AAGAGTATGAGTATTCAACATTTCCGTGTCGCCCTTATTCCCTTTTTTGCGGCATTTTGC

CTTCCTGTTTTTGCTCACCCAGAAACGCTGGTGAAAGTAAAAGATGCTGAAGATCAGTTG

GGTGCACGAGTGGGTTACATCGAACTGGATCTCAACAGCGGTAAGATCCTTGAGAGTTTT

CGCCCCGAAGAACGTTTTCCAATGATGAGCACTTTTAAAGTTCTGCTATGTGGCGCGGTA

TTATCCCGTATTGACGCCCGGGCAAGAGCAACTCGGTCGCCGCATACACTATTCTCAGAA

TGACTTGGGTTGAGTACTCACCAGTCACAGAAAAGCATCTTACGGATGGCATGACAGTAA

GAGATTATGCAGTGCTGCATACCATGAGTGATACACTGCGGCCAACTTACTTCTGACACG

ATCGGAGGACCGAAGGAGCTAACGCTTTTTGCACAACATGGGGGATCATTTAACTCGCCT

TGATTCGTGGTACGGAGCTGAATTGAGCCAACAACGACCGAGCGTGAACCAGATGCCTGT

AGCCAGTGGCAACACGGTGTGGCCAACCTAATATAACGCTGGGCGCAAACTATCT

>13 fad2-1f

GCTAAGCTCTTTCAGGGTTCACATTCAAACCCTCCATTCAGTGTTGGCCAACTCAAGAAA

GCAATTCCACCACATTGCTTTGAACGTTCTCTTTTCATATCATTCTCCTATGTTGTCTAT

GATCTCTTAATGGCCTACTTACTCTTCTACATTGCCACCACTTATTTCCACCAGCTTCCA

TACCCATTTTCCTTCCTTGCTTGGCCAATCTATTGGGCCATCCAAGGCTGCATTCTCACT

GGTGTTTGGGTGATTGCTCATGAGTGTGGCCACCATGCCTTCAGCAAGTATCAACTTGTT

GATGACATGGTTGGTTTGATCCTTCACTCTTGTCTATTAGTCCCTTATTTCTCATGGAAA

ATCAGCCACCGCCACCTCCACTCCAACACCGGTTCCCTCGACCGCGACTAAGTGTTTGTC

CTGAAACCAAAATCAAAGGTATCACGGTATAACAAGTACATGAACAATCCACTAGAGAGG

GCTATTTCCCTTTTCATCACACTCACACTAGGATGGCCCTGATAATAATGACGTCAGAAT

TCTCGAGTCGGGGAAATGTGCGCGGAACCCCTATTTGTTTATTTTTCTAAATACATTCAA

ATATGTATCCGCTCATGAGACAATAACCCTGATAAATGCTTCAATAATATTGAAAAAGGA

AGAGTATGAGTATTCAACATTTCCGTGTCGCCCTTATTCCCTTTTTTGCGGCATTTTGCC

TTCCTGTTTTTGCTCACCCAGAAACGCTGGTGAAAGTAAAAGATGCTGAAGATCAGTTGG

GTGCACGAGTGGGTTACATCGAACTGGATCTCAACAGCGGTAAGATCCTTGAGAGTTTTC

GCCCCGAAGAACGTTTTCCAATGATGAGCACTTTTAAAGTTCTGCTATGTGGCGCGGTAT

TATCCCGTATTGACGCCCGGCAAGAGCAACTCGGTCGCCGCATACACTATTCTCAGAATG

ACTTGGTTGAGTACTCCACCAGTCACAGAAAAGCATCTTACGGATGGCATGACAGTAAGA

GAATTATGCAGTGCTGCCATAACATGAGTGATAACACTGCGGCAACTTACTTCTGACACG

ATCGGAGACCGAGAGCTAACCGCTTTTTTGCACAACATGGGGGAATCATGTAACTCGCTG

AATCGTGGAACGGAGCTGATGAAGCATACTACGACGAGCGGTGAACCCCAGATGCCTGTA

GCCATTGCACCACGGTGGCGCAATCTTATTACCGTGGCGGAACTTACAT

>14 fad2-1f

ACCGAAGCTCTTTCAGGGTTCACATTCAAACCCTCCATTCAGTGTTGGCCAACTCAAGAA

AGCAATTCCACCACATTGCTTTGAACGTTCTCTTTTCATATCATTCTCATATGTTGTCTA

TGATCTCTTAATGGCCTACTTACTCTTCTACATTGCCACCACTTATTTCCACAAGCTTCC

ATACCCATTTTCCTTCCTTGCTTGGCCAATCTATTGGGCCATCCAAGGCTGCATTCTCAC

CGGTGTTTGGGTGATTGCTCATGAGTGTGGCCACCATGCCTTCAGCAAGTACCAACTTGT

TGATGACATGGTTGGTTTGACCCTTCACTCTTGTCTATTAGTTCCTTATTTCTCATGGAA

AATCAGCCACCGCCGCCACCACTCCAACACAGGTTCCCTCGACCGCGACGAAGTGTTTGT

CCCGAAACCAAAATCAAAGGTATCATGGTATAACAAGTACATGAACAATCCACCAGGGAG

GGCTATTTCCCTTTTCATCACACTCACACTAGGATGGCCCTGATAATAATGACGTCAGAA

TTCTCGAGTCGGGGAAATGTGCGCGGAACCCCTATTTGTTTATTTTTCTAAATACATTCA

AATATGTATCCGCTCATGAGACAATAACCCTGATAAATGCTTCAATAATATTGAAAAAGG

AAGAGTATGAGTATTCAACATTTCCGTGTCGCCCTTATTCCCTTTTTTGCGGCATTTTGC

CTTCCTGTTTTTGCTCACCCAGAAACGCTGGTGAAAGTAAAAGATGCTGAAGATCAGTTG

GGTGCACGAGTGGGTTACATCGAACTGGATCTCAACAGCGGTAAGATCCTTGAGAGTTTT

CGCCCCGAAGAACGTTTTCCAATGATGAGCACTTTTAAAGTTCTGCTATGTGGCGCGGTA

TTATCCCGTATTGACGCCGGGCAAGAGCAACTCGGTCGCCGCATACACTATTCTCAGAAT

GACTTGGGTTGAGTACTCACCAGTCACAGAAAAGCATCTTACGGATGGCATGACAGTAGA

GAATTATGCAGTGCTGCCATAACCATGAGTGATAACACTGCGGCCACTTACTTCTGACAC

GATCGGAGGACCGAGGAGCTACGCTTTTTGCACAACATGGGGGATCATGTAACTCGGCTG

ATCGTGGGAACCGGAGCTGAATGAAGCATACTAACCGACGAGCGGACCCAGAGTGCCTGT

AGCAATGGCACCACGTTGGGCCGAACTATTTAACTGCGCGGGAACACTC

>15 fad2-1f

AAAAAGCTCTTTCAGGGTTCACATTCAACCCTCCATTCAGTGTTGGCCAACTCAAGAAAG

CAATTCCACCACATTGCTTTGAACGTTCTCTTTTCATATCATTCTCATATGTTGTCTATG

ATCTCTTAATGGCCTACTTACTCTTCTACATTGCCACCACTTATTTCCACAAGCTTCCAT

ACCCATTTTCCTTCCTTGCTTGGCCAATCTATTGGGCCATCCAAGGCTGCATTCTCACCG

GTGTTTGGGTGATTGCTCATGAGTGTGGCCACCATGCCTTCAGCAAGTACCAACTTGTTG

ATGACATGGTTGGTTTGACCCTTCACTCTTGTCTATTAGTTCCTTATTTCTCATGGAAAA

TCAGCCACCGCCGCCACCACTCCAACACAGGTTCCCTCGACCGCGACGAAGTGTTTGTCC

CGAAACCAAAATCAAAGGTATCATGGTATAACAAGTACATGAACAATCCACCAGGGAGGG

CTATTTCCCTTTTCATCACACTCACACTAGGATGGCCCTGATAATAATGACGTCAGAATT

CTCGAGTCGGGGAAATGTGCGCGGAACCCCTATTTGTTTATTTTTCTAAATACATTCAAA

TATGTATCCGCTCATGAGACAATAACCCTGATAAATGCTTCAATAATATTGAAAAAGGAA

GAGTATGAGTATTCAACATTTCCGTGTCGCCCTTATTCCCTTTTTTGCGGCATTTTGCCT

TCCTGTTTTTGCTCACCCAGAAACGCTGGTGAAAGTAAAAGATGCTGAAGATCAGTTGGG

TGCACGAGTGGGTTACATCGAACTGGATCTCAACAGCGGTAAGATCCTTGAGAGTTTTCG

CCCCGAAGAACGTTTTCCAATGATGAGCACTTTTAAAGTTCTGCTATGTGGCGCGGTATT

ATCCCGTATTGACGCCGGGCAAGAGCAACTCGGTCGCCGCATACACTATTCTCAGAATGA

CTTGGTTGAGTACTCACCAGTCACAGAAAAGCATCTTACGGATGGCATGACAGTAGAGAT

TATGCAGTGCTGCCATACCATGAGTGATACACTGCGGCACTTACTTCTGACACGATCGGA

GGACCGAGGAGCTACCGCTTTTTGCACACATGGGGGATCATGTACTCCGCTGATCGTGGA

CCGACCTGATGAGGCATACCAACGACGAGGCGTGACCCACGATGCTGTAGACATTGGCAC

ACGGTGGCCCACCATTTTATCGTTGCGAAACTTACTGTAACCTCCTAGGC

>16 fad2-1f

GCAAAGCTCTTTCAGGGTTCACATTCAAACCCTCCATTCAGTGTTGGCCAACTCAAGAAA

GCAATTCCACCACATTGCTTTGAACGTTCTCTTTTCATATCATTCTCCTATGTTGTCTAT

GATCTCTTAGTGGCCTACTTACTCTTCTACATTGCCACCACTTATTTCCACAAGCTTCCA

TACCCATTTTCCTTCCTTGCTTGGCCAATCTATTGGGCCATCCAAGGCTGCATTCTCACT

GGTGTTTGGGTGATTGCTCATGAGTGTGGCCACCATGCCTTCAGCAAGTACCAACTTGTT

GATGACATGGTTGGTTTGACCCTTCACTCTTGTCTATTAGTTCCTTATTTCTCATGGAAA

ATCAGTCACCGCCGCCACCACTCCAACACCGGTTCCCTCGACCGCAACGAAGTGTTTGTC

CCAAAACCAAAATCAAAGGTATCATGGTATAACAAGTACATGAACAATCCACCAGGGAGG

GCTATCTCCCTCTTCATCACACTCACACTAGGATGGCCCTGATAATAATGACGTCAGAAT

TCTCGAGTCGGGGAAATGTGCGCGGAACCCCTATTTGTTTATTTTTCTAAATACATTCAA

ATATGTATCCGCTCATGAGACAATAACCCTGATAAATGCTTCAATAATATTGAAAAAGGA

AGAGTATGAGTATTCAACATTTCCGTGTCGCCCTTATTCCCTTTTTTGCGGCATTTTGCC

TTCCTGTTTTTGCTCACCCAGAAACGCTGGTGAAAGTAAAAGATGCTGAAGATCAGTTGG

GTGCACGAGTGGGTTACATCGAACTGGATCTCAACAGCGGTAAGATCCTTGAGAGTTTTC

GCCCCGAAGAACGTTTTCCAATGATGAGCACTTTTAAAGTTCTGCTATGTGGCGCGGTAT

TATCCCGTATTGACGCCGGGCAAGAGCAACTCGGTCGCCGCATACACTATTCTCAGAATG

ACTTGGTTGAGTACTCACCAGTCACAGAAAAGCATCTTACGGATGGCATGACAGTAGAGA

ATTATGCAGTGCTGCATACCATGAGTGATACACTGCGGCACTTACTTCTGACACGATCGG

AGGACCGAGAGCTAACGCTTTTTGCCACATGGGGGATCATGTACTCGCCTGATCGGTGGA

CGGAGCTGATGAAGCCATACCTACCGACGGAGCGGTGAACCCCGATGCTTAGCAATTGAT

ACACGGTGGCCCACACTAATAACTTGGGCGACCTACCTTAATC

>17 fad2-1f

CCGAAGAGCTCTTTCAGGGTTCACATTCAAACCCTCCATTCAGTGTTGGCCAACTCAAGA

AAGCAATTCCACCACATTGCTTTGAACGTTCTCTTTTCATATCATTCTCCTATGTTGTCT

ATGATCTCTTAATGGCCTACTTACTCTTCTACATTGCCACCACTTATTTCCACCAGCTTC

CATACCCATTTTCCTTCCTTGCTTGGCCAATCTATTGGGCCATCCAAGGCTGCATTCTCA

CTGGTGTTTGGGTGATTGCTCATGAGTGTGGCCACCATGCCTTCAGCAAGTATCAACTTG

TTGATGACATGGTTGGTTTGATCCTTCACTCTTGTCTATTAGTCCCTTATTTCTCATGGA

AAATCAGCCACCGCCACCTCCACTCCAACACCGGTTCCCTCGACCGCGACTAAGTGTTTG

TCCTGAAACCAAAATCAAAGGTATCACGGTATAACAAGTACATGAACAATCCACTAGAGA

GGGCTATTTCCCTTTTCATCACACTCACACTAGGATGGCCCTGATAATAATGACGTCAGA

ATTCTCGAGTCGGGGAAATGTGCGCGGAACCCCTATTTGTTTATTTTTCTAAATACATTC

AAATATGTATCCGCTCATGAGACAATAACCCTGATAAATGCTTCAATAATATTGAAAAAG

GAAGAGTATGAGTATTCAACATTTCCGTGTCGCCCTTATTCCCTTTTTTGCGGCATTTTG

CCTTCCTGTTTTTGCTCACCCAGAAACGCTGGTGAAAGTAAAAGATGCTGAAGATCAGTT

GGGTGCACGAGTGGGTTACATCGAACTGGATCTCAACAGCGGTAAGATCCTTGAGAGTTT

TCGCCCCGAAGAACGTTTTCCAATGATGAGCACTTTTAAAGTTCTGCTATGTGGCGCGGT

ATTATCCCGTATTGACGCCGGGCAAGAGCAACTCGGTCGCCGCATACACTATTCTCAGAA

TGACTTGGGTTGAGTACTCACCAGTCACAGAAAAGCATCTTACGGATGGCATGACAGTAA

GAGAATTATGCAGTGCTGCCATACCATGAGTGATACACTGCGGCAACTTACTTCTGACAC

GATCGGAGGACCGAAGGAGCTACCGCTTTTTGCACACATGGGGGATCATGTAACTCGCCT

GATCGTGGCACCGGAGCTGATGGAGCCAAACGAACGACCGAGGTGACCACCACCGAATGC

TGTAGCATGCACACGTTTGGCCAAACTATTAACTTGGCGAAACATAACTAACTCTCTAGT

GCC

>18 fad2-1f

CCCGAGCTCTTTCAGGGTTCACATTCAACCCTCCATTCAGTGTTGGCCAACTCAAGAAAG

CAATTCCACCACATTGCTTTGAACGTTCTCTTTTCATATCATTCTCCTATGTTGTCTATG

ATCTCTTAGTGGCCTACTTACTCTTCTACATTGCCACCACTTATTTCCACAAGCTTCCAT

ACCCATTTTCCTTCCTTGCTTGGCCAATCTATTGGGCCATCCAAGGCTGCATTCTCACTG

GTGTTTGGGTGATTGCTCATGAGTGTGGCCACCATGCCTTCAGCAAGTACCAACTTGTTG

ATGACATGGTTGGTTTGACCCTTCACTCTTGTCTATTAGTTCCTTATTTCTCATGGAAAA

TCAGCCACCGCCGCCACCACTCCAACACCGGTTCCCTCGACCGCAACGAAGTGTTTGTCC

CAAAACCAAAATCAAAGGTATCATGGTATAACAAGTACATGAACAATCCACCAGGGAGGG

CTATCTCCCTCTTCATCACACTCACACTAGGATGGCCCTGATAATAATGACGTCAGAATT

CTCGAGTCGGGGAAATGTGCGCGGAACCCCTATTTGTTTATTTTTCTAAATACATTCAAA

TATGTATCCGCTCATGAGACAATAACCCTGATAAATGCTTCAATAATATTGAAAAAGGAA

GAGTATGAGTATTCAACATTTCCGTGTCGCCCTTATTCCCTTTTTTGCGGCATTTTGCCT

TCCTGTTTTTGCTCACCCAGAAACGCTGGTGAAAGTAAAAGATGCTGAAGATCAGTTGGG

TGCACGAGTGGGTTACATCGAACTGGATCTCAACAGCGGTAAGATCCTTGAGAGTTTTCG

CCCCGAAGAACGTTTTCCAATGATGAGCACTTTTAAAGTTCTGCTATGTGGCGCGGTATT

ATCCCGTATTGACGCCGGGCAAGAGCAACTCGGTCGCCGCATACACTATTCTCAGAATGA

CTTGGTTGAGTACTCACCAGTCACAGAAAAGCATCTTACGGATGGCATGACAGTAGGAGG

ATTATGCAGTGCTGCCATACCCATGGAGTGATAACACTGCGCCACTTACTTCTGACACGA

TCGGAGGACCGAAGGAGCTACGGCTTTTGCACAACATGGGGGATCCATGTAACTCGCCTT

GATCGTGGGAACCGGAGCTGATGAGGCAACTACGGACGGCGTGAACCCACGATGCTGTAC

CATGGCTACACGTGCCGACCATATACTGGCGCAGACTAACTTACT

>19 fad2-1f

ACAGGAGCTCTTTCAGGGTTCACATTCAAACCCTCCATTCAGTGTTGGCCAACTCAAGAA

AGCAATTCCACCACATTGCTTTGAACGTTCTCTTTTCATATCATTCTCCTATGTTGTCTA

TGATCTCTTAGTGGCCTACTTACTCTTCTACATTGCCACCACTTATTTCCACAAGCTTCC

ATACCCATTTTCCTTCCTTGCTTGGCCAATCTATTGGGCCATCCAAGGCTGCATTCTCAC

TGGTGTTTGGGTGATTGCTCATGAGTGTGGCCACCATGCCTTCAGCAAGTACCAACTTGT

TGATGACATGGTTGGTTTGACCCTTCACTCTTGTCTATTAGTTCCTTATTTCTCATGGAA

AATCAGCCACCGCCGCCACCACTCCAACACCGGTTCCCTCGACCGCAACGAAGTGTTTGT

CCCAAAACCAAAATCAAAGGTATCATGGTATAACAAGTACATGAACAATCCACCAGGGAG

GGCTATCTCCCTCTTCATCACACTCACACTAGGATGGCCCTGATAATAATGACGTCAGAA

TTCTCGAGTCGGGGAAATGTGCGCGGAACCCCTATTTGTTTATTTTTCTAAATACATTCA

AATATGTATCCGCTCATGAGACAATAACCCTGATAAATGCTTCAATAATATTGAAAAAGG

AAGAGTATGAGTATTCAACATTTCCGTGTCGCCCTTATTCCCTTTTTTGCGGCATTTTGC

CTTCCTGTTTTTGCTCACCCAGAAACGCTGGTGAAAGTAAAAGATGCTGAAGATCAGTTG

GGTGCACGAGTGGGTTACATCGAACTGGATCTCAACAGCGGTAAGATCCTTGAGAGTTTT

CGCCCCGAAGAACGTTTTCCAATGATGAGCACTTTTAAAGTTCTGCTATGTGGCGCGGTA

TTATCCCGTATTGACGCCGGGCAAGAGCAACTCGGTCGCCGCATACACTATTCTCAGAAT

GACTTGGTTGAGTACTCACCAGTCACAGAAAAGCATCTTACGGATGGCATGACAGTAGAG

AATTATGCAGTGCTGCCATACATGAGTGATACACTGCGGCACTACTTCTGACACGATCGG

AGGAACGAGAGCTTACCGCTTTTTTGCACAACATGGGGGATCATGTACTCGGCTGGATCG

TGGACGAACCTGAATGAAGCCAACCACGACAGCGTGAACCCGATGCTGTAGCATGGCCAC

AACGTTGGCCACCATTACCTGGGCGACTACGTTAC

>20 fad2-1f

ACGAAGCTCTTTCAGGGTTCACATTCAACCCTCCATTCAGTGTTGGCCAACTCAAGAAAG

CAATTCCACCACATTGCTTTGAACGTTCTCTTTTCATATCATTCTCATATGTTGTCTATG

ATCTCTTAATGGCCTACTTACTCTTCTACATTGCCACCACTTATTTCCACAAGCTTCCAT

ACCCATTTTCCTTCCTTGCTTGGCCAATCTATTGGGCCATCCAAGGCTGCATTCTCACCG

GTGTTTGGGTGATTGCTCATGAGTGTGGCCACCATGCCTTCAGCAAGTACCAACTTGTTG

ATGACATGGTTGGTTTGACCCTTCACTCTTGTCTATTAGTTCCTTATTTCTCATGGAAAA

TCAGCCACCGCCGCCACCACTCCAACACAGGTTCCCTCGACCGCGACGAAGTGTTTGTCC

CGAAACCAAAATCAAAGGTATCATGGTATAACAAGTACATGAACAATCCACCAGGGAGGG

CTATTTCCCTTTTCATCACACTCACACTAGGATGGCCCTGATAATAATGACGTCAGAATT

CTCGAGTCGGGGAAATGTGCGCGGAACCCCTATTTGTTTATTTTTCTAAATACATTCAAA

TATGTATCCGCTCATGAGACAATAACCCTGATAAATGCTTCAATAATATTGAAAAAGGAA

GAGTATGAGTATTCAACATTTCCGTGTCGCCCTTATTCCCTTTTTTGCGGCATTTTGCCT

TCCTGTTTTTGCTCACCCAGAAACGCTGGTGAAAGTAAAAGATGCTGAAGATCAGTTGGG

TGCACGAGTGGGTTACATCGAACTGGATCTCAACAGCGGTAAGATCCTTGAGAGTTTTCG

CCCCGAAGAACGTTTTCCAATGATGAGCACTTTTAAAGTTCTGCTATGTGGCGCGGTATT

ATCCCGTATTGACGCCGGGCAAGAGCAACTCGGTCGCCGCATACACTATTCTCAGAATGA

CTTGGTTGAGTACTCACCAGTCACAGAAAAGCATCTTACGGATGGCATGACAGTAAGAGA

ATTATGCAGTGCTGCCATACCATGAGTGATACACTGCGGCACTTACTTCTGACACGATCG

GAGGACCGAGGAGCTACCGCTTTTTGCACAACATGGGGGATCATGTAACCTCGCTGATCG

TGGGACCGGAGCTGATGAAGCATACCAAACGACGAGCGGACCCACGATGCCTGTAGCCAA

TGCACCACGTTGCGCTAACTATAAACTGTGGCGAAACATACTTTTA

>21 fad2-1f

AAGAAGCTCTTTCAGGGTTCACATTCAAACCCTCCATTCAGTGTTGGCCAACTCAAGAAA

GCAATTCCACCACATTGCTTTGAACGTTCTCTTTTCATATCATTCTCCTATGTTGTCTAT

GATCTCTTAGTGGCCTACTTACTCTTCTACATTGCCACCACTTATTTCCACAAGCTTCCA

TACCCATTTTCCTTCCTTGCTTGGCCAATCTATTGGGCCATCCAAGGCTGCATTCTCACT

GGTGTTTGGGTGATTGCTCATGAGTGTGGCCACCATGCCTTCAGCAAGTACCAACTTGTT

GATGACATGGTTGGTTTGACCCTTCACTCTTGTCTATTAGTTCCTTATTTCTCATGGAAA

ATCAGCCACCGCCGCCACCACTCCAACACCGGTTCCCTCGACCGCAACGAAGTGTTTGTC

CCAAAACCAAAATCAAAGGTATCATGGTATAACAAGTACATGAACAATCCACTAGAGAGG

GCTATTTCCCTTTTCATCACACTCACACTAGGATGGCCCTGATAATAATGACGTCAGAAT

TCTCGAGTCGGGGAAATGTGCGCGGAACCCCTATTTGTTTATTTTTCTAAATACATTCAA

ATATGTATCCGCTCATGAGACAATAACCCTGATAAATGCTTCAATAATATTGAAAAAGGA

AGAGTATGAGTATTCAACATTTCCGTGTCGCCCTTATTCCCTTTTTTGCGGCATTTTGCC

TTCCTGTTTTTGCTCACCCAGAAACGCTGGTGAAAGTAAAAGATGCTGAAGATCAGTTGG

GTGCACGAGTGGGTTACATCGAACTGGATCTCAACAGCGGTAAGATCCTTGAGAGTTTTC

GCCCCGAAGAACGTTTTCCAATGATGAGCACTTTTAAAGTTCTGCTATGTGGCGCGGTAT

TATCCCGTATTGACGCCGGGCAAGAGCAACTCGGTCGCCGCATACACTATTCTCAGAATG

ACTTGGTTGAGTACTCACCAGTCACAGAAAAGCATCTTACGGATGGCATGACAGTAGAGA

TTATGCAGTGCTGCCATACCATGAGTGATACACTGCGGCCAACTTACTTCTGACACGATC

GGAGGACGAGGAGCTAACCGCTTTTTTGCACACATGGGGGATCATGTAACTCGCTGAATC

GTGGAACGGAGCTGGATGAGCCATACGACCGACGAGGCGGTGAAACCCAGATGCTGTAGC

ATGGCACTCACGGTGCCCAAAACTATTTACTGGGCCGACCTATC

>22 fad2-1f

GACGGGAGCTCTTTCAGGGTTCACATTCAACCCTCCATTCAGTGTTGGCCAACTCAAGAA

AGCAATTCCACCACATTGCTTTGAACGTTCTCTTTTCATATCATTCTCCTATGTTGTCTA

TGATCTCTTAATGGCCTACTTACTCTTCTACATTGCCACCACTTATTTCCACCAGCTTCC

ATACCCATTTTCCTTCCTTGCTTGGCCAATCTATTGGGCCATCCAAGGCTGCATTCTCAC

TGGTGTTTGGGTGATTGCTCATGAGTGTGGCCACCATGCCTTCAGCAAGTATCAACTTGT

TGATGACATGGTTGGTTTGATCCTTCACTCTTGTCTATTAGTCCCTTATTTCTCATGGAA

AATCAGCCACCGCCACCTCCACTCCAACACCGGTTCCCTCGACCGCGACTAAGTGTTTGT

CCTGAAACCAAAATCAAAGGTATCACGGTATAACAAGTACATGAACAATCCACTAGAGAG

GGCTATTTCCCTTTTCATCACACTCACACTAGGATGGCCCTGATAATAATGACGTCAGAA

TTCTCGAGTCGGGGAAATGTGCGCGGAACCCCTATTTGTTTATTTTTCTAAATACATTCA

AATATGTATCCGCTCATGAGACAATAACCCTGATAAATGCTTCAATAATATTGAAAAAGG

AAGAGTATGAGTATTCAACATTTCCGTGTCGCCCTTATTCCCTTTTTTGCGGCATTTTGC

CTTCCTGTTTTTGCTCACCCAGAAACGCTGGTGAAAGTAAAAGATGCTGAAGATCAGTTG

GGTGCACGAGTGGGTTACATCGAACTGGATCTCAACAGCGGTAAGATCCTTGAGAGTTTT

CGCCCCGAAGAACGTTTTCCAATGATGAGCACTTTTAAAGTTCTGCTATGTGGCGCGGTA

TTATCCCGTATTGACGCCCGGCAAGAGCAACTCGGTCGCCGCATACACTATTCTCAGAAT

GACTTGGTTGAGTACTCACCAGTCACAGAAAAGCATCTTACGGATGGCATGACAGTAGAG

ATTATGCAGTGCTGCCATAACCATGAGTGATACACTGCGGCACTTACTCTGACACGATCG

GAGGACGAGGAGCTACCGCTTTTTTGCACACATGGGGGATCATGTACTCGCTGATCGGTG

GACGGGCTGAATGAGCCATACTACGACGAGCCGTGAACACCCGATGCCTGTAGCATTGCC

ACACGGTGGGTAACCATATAAACTGGGGGGAACATCAAC

>23 fad2-1f

ACGGAGCTCTTTCAGGGTTCACATTCAAACCCTCCATTCAGTGTTGGCCAACTCAAGAAA

GCAATTCCACCACATTGCTTTGAACGTTCTCTTTTCATATCATTCTCATATGTTGTCTAT

GATCTCTTAATGGCCTACTTACTCTTCTACATTGCCACCACTTATTTCCACAAGCTTCCA

TACCCATTTTCCTTCCTTGCTTGGCCAATCTATTGGGCCATCCAAGGCTGCATTCTCACC

GGTGTTTGGGTGATTGCTCATGAGTGTGGCCACCATGCCTTCAGCAAGTACCAACTTGTT

GATGACATGGTTGGTTTGACCCTTCACTCTTGTCTATTAGTTCCTTATTTCTCATGGAAA

ATCAGCCACCGCCGCCACCACTCCAACACAGGTTCCCTCGACCGCGACGAAGTGTTTGTC

CCGAAACCAAAATCAAAGGTATCATGGTATAACAAGTACATGAACAATCCACCAGGGAGG

GCTATTTCCCTTTTCATCACACTCACACTAGGATGGCCCTGATAATAATGACGTCAGAAT

TCTCGAGTCGGGGAAATGTGCGCGGAACCCCTATTTGTTTATTTTTCTAAATACATTCAA

ATATGTATCCGCTCATGAGACAATAACCCTGATAAATGCTTCAATAATATTGAAAAAGGA

AGAGTATGAGTATTCAACATTTCCGTGTCGCCCTTATTCCCTTTTTTGCGGCATTTTGCC

TTCCTGTTTTTGCTCAATGACACCCACTGGTGAAAAGAGTAGATGGTGAAGATCATGTGC

GAGCACTAGGGGGTGCCATCCCACTGAATCTCAGCAGCACAGAGACTTCCGACAGAGCCG

TGCCGGATGATGCCTCAGAATGATGAGCACTTTCTAAGTTCTCTGATTTATCGCGTGCTG

ATCCCGTGTGACTCCCGCTTAGAGCGACATTGATACAGCGTACACTATTCTCAGATTGAT

ATCACTGAGTCAAAGCTGACACAAGAAGATAGTACGCGCCAGCATGACATAATGCGATTA

CGCAATGCTGCGTACACGGTCTACAGCAGTGCGGTCTACTACTTACGACACGATCTGGCG

GCCGTAAGACCTGAACTACTCTCTCCGACTACATCGAGTTTATCATGTACTATGCTGCAT

GGTCGGACTCGGTGAGCCGATTACGATTCGAGCTAGAGTCTGTTAACTTGCAGTACTGTA

GCAAGCAACGCTGTCATGACGGATTTGCTTGGCGGACTATCATACCCCC

>24 fad2-1f

ACATAGCTCTTTCATGGTTCACATTCAAACCCTCCATTCAGTGTTGGCCAACTCAAGAAA

GCAATTCCACCACATTGCTTTGAACGTTCTCTTTTCATATCATTCTCATATGTTGTCTAT

GATCTCTTAATGGCCTACTTACTCTTCTACATTGCCACCACTTATTTCCACAAGCTTCCA

TACCCATTTTCCTTCCTTGCTTGGCCAATCTATTGGGCCATCCAAGGCTGCATTCTCACC

GGTGTTTGGGTGATTGCTCATGAGTGTGGCCACCATGCCTTCAGCAAGTATCAACTTGTT

GATGACATGGTTGGTTTGATCCTTCACTCTTGTCTATTAGTCCCTTATTTCTCATGGAAA

ATCAGCCACCGCCACCTCCACTCCAACACCGGTTCCCTCGACCGCGACTAAGTGTTTGTC

CTGAAACCAAAATCAAAGGTATCACGGTATAACAAGTACATGAACAATCCACTAGAGAGG

GCTATTTCCCTTTTCATCACACTCACACTAGGATGGCCCTGATAATAATGACGTCAGAAT

TCTCGAGTCGGGGAAATGTGCGCGGAACCCCTATTTGTTTATTTTTCTAAATACATTCAA

ATATGTATCCGCTCATGAGACAATAACCCTGATAAATGCTTCAATAATATTGAAAAAGGA

AGAGTATGAGTATTCAACATTTCCGTGTCGCCCTTATTCCCTTTTTTGCGGCATTTTGCC

TTCCTGTTTTTGCTCACCCAGAAACGCTGGTGAAAGTAAAAGATGCTGAAGATCAGTTGG

GTGCACGAGTGGGTTACATCGAACTGGATCTCAACAGCGGTAAGATCCTTGAGAGTTTTC

GCCCCGAAGAACGTTTTCCAATGATGAGCACTTTTAAAGTTCTGCTATGTGGCGCGGTAT

TATCCCGTATTGACGCCGGGCAAGAGCAACTCGGTCGCCGCATACACTATTCTCAGAATG

ACTTGGTTGAGTACTCACCAGTCACAGAAAAGCATCTTACGGATGGCATGACAGTAGAGA

TTATGCAGTGCTGCCATACCATGAGTGATACACTGCGGCAACTTACTTCTGACAACGATC

GGAGACCGAGGAGCTACCGCTTTTTGCACACATGGGGGGATCATGTACTCCGCCTGATCG

GTGGACGGAGCTGATGAGCATACTACGACGAGCGTGACACACCGATGCTGTAGCATGGCA

CACGTGGCCACCTATTAACTTGGCGAACATCACCTTAACCTCTTAGGCCTCG

>25 fad2-1f

GAACGAGGCTCATTATGGTTCACATTCAACCCTCCATTCAGTGTTGGCCAACTCAAGAAA

GCAATTCCACCACATTGCTTTGAACGTTCTCTTTTCATATCATTCTCCTATGTTGTCTAT

GATCTCTTAATGGCCTACTTACTCTTCTACATTGCCACCACTTATTTCCACCAGCTTCCA

TACCCATTTTCCTTCCTTGCTTGGCCAATCTATTGGGCCATCCAAGGCTGCATTCTCACT

GGTGTTTGGGTGATTGCTCATGAGTGTGGCCACCATGCCTTCAGCAAGTATCAACTTGTT

GATGACATGGTTGGTTTGATCCTTCACTCTTGTCTATTAGTCCCTTATTTCTCATGGAAA

ATCAGCCACCGCCACCTCCACTCCAACACCGGTTCCCTCGACCGCGACTAAGTGTTTGTC

CTGAAACCAAAATCAAAGGTATCACGGTATAACAAGTACATGAACAATCCACTAGAGAGG

GCTATTTCCCTTTTCATCACACTCACACTAGGATGGCCCTGATAATAATGACGTCAGAAT

TCTCGAGTCGGGGAAATGTGCGCGGAACCCCTATTTGTTTATTTTTCTAAATACATTCAA

ATATGTATCCGCTCATGAGACAATAACCCTGATAAATGCTTCAATAATATTGAAAAAGGA

AGAGTATGAGTATTCAACATTTCCGTGTCGCCCTTATTCCCTTTTTTGCGGCATTTTGCC

TTCCTGTTTTTGCTCACCCAGAAACGCTGGTGAAAGTAAAAGATGCTGAAGATCAGTTGG

GTGCACGAGTGGGTTACATCGAACTGGATCTCAACAGCGGTAAGATCCTTGAGAGTTTTC

GCCCCGAAGAACGTTTTCCAATGATGAGCACTTTTAAAGTTCTGCTATGTGGCGCGGTAT

TATCCCGTATTGACGCCGGGCAAGAGCAACTCGGTCGCCGCATACACTATTCTCAGAATG

ACTTGGTTGAGTACTCACCAGTCACAGAAAAGCATCTTACGGATGGCATGACAGTAAGAG

AATTATGCAGTGCTGCCATAACCATGAGTGATAACACTGCGGCCAACTTACTTCTGACAC

GATCGGAGGACCGAAGGAGCTACGCTTTTTGCACACATGGGGGGATCATGTAACTCGCCT

GATCGTGGACGGAGCTGAATGAGGCATACAACGACGAGCGGAACCCACGATGCCTGTAGC

ATTGCACAACGGTTGCCGCCAAACCTATTAACCGTGGGCTGAAACTAC

>26 fad2-1f

AGCTAAGCTCATTCAGGGTTCACATTCAACCCTCCATTCAGTGTTGGCCAACTCAAGAAA

GCAATTCCACCACATTGCTTTGAACGTTCTCTTTTCATATCATTCTCCTATGTTGTCTAT

GATCTCTTAGTGGCCTACTTACTCTTCTACATTGCCACCACTTATTTCCACAAGCTTCCA

TACCCATTTTCCTTCCTTGCTTGGCCAATCTATTGGGCCATCCAAGGCTGCATTCTCACT

GGTGTTTGGGTGATTGCTCATGAGTGTGGCCACCATGCCTTCAGCAAGTACCAACTTGTT

GATGACATGGTTGGTTTGACCCTTCACTCTTGTCTATTAGTTCCTTATTTCTCATGGAAA

ATCAGCCACCGCCGCCACCACTCCAACACCGGTTCCCTCGACCGCAACGAAGTGTTTGTC

CCAAAACCAAAATCAAAGGTATCATGGTATAACAAGTACATGAACAATCCACCAGGGAGG

GCTATCTCCCTCTTCATCACACTCACACTAGGATGGCCCTGATAATAATGACGTCAGAAT

TCTCGAGTCGGGGAAATGTGCGCGGAACCCCTATTTGTTTATTTTTCTAAATACATTCAA

ATATGTATCCGCTCATGAGACAATAACCCTGATAAATGCTTCAATAATATTGAAAAAGGA

AGAGTATGAGTATTCAACATTTCCGTGTCGCCCTTATTCCCTTTTTTGCGGCATTTTGCC

TTCCTGTTTTTGCTCACCCAGAAACGCTGGTGAAAGTAAAAGATGCTGAAGATCAGTTGG

GTGCACGAGTGGGTTACATCGAACTGGATCTCAACAGCGGTAAGATCCTTGAGAGTTTTC

GCCCCGAAGAACGTTTTCCAATGATGAGCACTTTTAAAGTTCTGCTATGTGGCGCGGTAT

TATCCCGTATTGACGCCGGGCAAGAGCAACTCGGTCGCCGCATACACTATTCTCAGAATG

ACTTGGGTTGAGTACTCACCAGTCACAGAAAAGCATCTTACGGATGGCATGACAGTAGAG

ATTATGCAGTGCTGCATACCATGAGTGATAACACTGCGGCAACTTACTCTGACAACGATC

GAGACCGAGAGCTACGCTTTTTGCCAACATGGGGATCATGTACTCCGCTGATCGTGGGAC

GGACTGATGAAGCCTACCAACGACGGCGTGAACCCCGATGCTGTAGCATTGACAACACGG

TGCGCATACCATATATACTGTGGCGAACCTACCTCTAC

>27 fad2-1f

ACAGAGCTCTTTCACGGTTCACATTCAAACCCTCCATTCAGTGTTGGCCAACTCAAGAAA

GCAATTCCACCACATTGCTTTGAACGTTCTCTTTTCATATCATTCTCCTATGTTGTCTAT

GATCTCTTAATGGCCTACTTACTCTTCTACATTGCCACCACTTATTTCCACCAGCTTCCA

TACCCATTTTCCTTCCTTGCTTGGCCAATCTATTGGGCCATCCAAGGCTGCATTCTCACT

GGTGTTTGGGTGATTGCTCATGAGTGTGGCCACCATGCCTTCAGCAAGTATCAACTTGTT

GATGACATGGTTGGTTTGATCCTTCACTCTTGTCTATTAGTCCCTTATTTCTCATGGAAA

ATCAGCCACCGCCACCTCCACTCCAACACCGGTTCCCTCGACCGCGACTAAGTGTTTGTC

CTGAAACCAAAATCAAAGGTATCACGGTATAACAAGTACATGAACAATCCACTAGAGAGG

GCTATTTCCCTTTTCATCACACTCACACTAGGATGGCCCTGATAATAATGACGTCAGAAT

TCTCGAGTCGGGGAAATGTGCGCGGAACCCCTATTTGTTTATTTTTCTAAATACATTCAA

ATATGTATCCGCTCATGAGACAATAACCCTGATAAATGCTTCAATAATATTGAAAAAGGA

AGAGTATGAGTATTCAACATTTCCGTGTCGCCCTTATTCCCTTTTTTGCGGCATTTTGCC

TTCCTGTTTTTGCTCACCCAGAAACGCTGGTGAAAGTAAAAGATGCTGAAGATCAGTTGG

GTGCACGAGTGGGTTACATCGAACTGGATCTCAACAGCGGTAAGATCCTTGAGAGTTTTC

GCCCCGAAGAACGTTTTCCAATGATGAGCACTTTTAAAGTTCTGCTATGTGGCGCGGTAT

TATCCCGTATTGACGCCGGGCAAGAGCAACTCGGTCGCCGCATACACTATTCTCAGAATG

ACTTGGTTGAGTACTCACCAGTCACAGAAAAGCATCTTACGGATGGCATGACAGTAGAGA

ATTATGCAGTGCTGCCATACCATGAGTGATACACTGCGCCACTTACTTCTGACACGATCG

GAGACCGAGGAGCTAACCGCTTTTTGCACACATGGGGGATCATGTACTCGCTGGATCGTG

GGACGGAGCTTGAATGAAGCATACCAACGACGAGGCGTGACACCCGACTGCTGTAGCATT

GGTCACACGTTGGCCTACATATTACTGGCCGACTACTTATCCTCTAGAGC

>28 fad2-1f

GACAAAGCTCTTTCAGGGTTCACATTCAACCCTCCATTCAGTGTTGGCCAACTCAAGAAA

GCAATTCCACCACATTGCTTTGAACGTTCTCTTTTCATATCATTCTCCTATGTTGTCTAT

GATCTCTTAATGGCCTACTTACTCTTCTACATTGCCACCACTTATTTCCACCAGCTTCCA

TACCCATTTTCCTTCCTTGCTTGGCCAATCTATTGGGCCATCCAAGGCTGCATTCTCACT

GGTGTTTGGGTGATTGCTCATGAGTGTGGCCACCATGCCTTCAGCAAGTATCAACTTGTT

GATGACATGGTTGGTTTGATCCTTCACTCTTGTCTATTAGTCCCTTATTTCTCATGGAAA

ATCAGCCACCGCCACCTCCACTCCAACACCGGTTCCCTCGACCGCGACTAAGTGTTTGTC

CTGAAACCAAAATCAAAGGTATCACGGTATAACAAGTACATGAACAATCCACTAGAGAGG

GCTATTTCCCTTTTCATCACACTCACACTAGGATGGCCCTGATAATAATGACGTCAGAAT

TCTCGAGTCGGGGAAATGTGCGCGGAACCCCTATTTGTTTATTTTTCTAAATACATTCAA

ATATGTATCCGCTCATGAGACAATAACCCTGATAAATGCTTCAATAATATTGAAAAAGGA

AGAGTATGAGTATTCAACATTTCCGTGTCGCCCTTATTCCCTTTTTTGCGGCATTTTGCC

TTCCTGTTTTTGCTCACCCAGAAACGCTGGTGAAAGTAAAAGATGCTGAAGATCAGTTGG

GTGCACGAGTGGGTTACATCGAACTGGATCTCAACAGCGGTAAGATCCTTGAGAGTTTTC

GCCCCGAAGAACGTTTTCCAATGATGAGCACTTTTAAAGTTCTGCTATGTGGCGCGGTAT

TATCCCGTATTGACGCCGGGCAAGAGCAACTCGGTCGCCGCATACACTATTCTCAGAATG

ACTTGGTTGAGTACTCACCAGTCACAGAAAAGCATCTTACGGATGGCATGACAGTAGAGA

ATTATGCAGTGCTGCCATACCATGAGTGATACACTGCGGCACTTACTTCTGACACGATCG

GAGGACGAGAGCTAACCGCTTTTTTGCACACATGGGGGATCATGTACCTCGCCTGATCGT

GGACGACTGAATGAGCATACCAACGACCGGCGTGACACCCAGATCTGTAGCATGGCACAC

GTTGGCGCAACTATTACCTTGGCCGAACATACACTAACCTCTAAGGCTC

>29 fad2-1f

GCAGGAGCTCTTTCAGGGTTCACATTCAACCCTCCATTCAGTGTTGGCCAACTCAAGAAA

GCAATTCCACCACATTGCTTTGAACGTTCTCTTTTCATATCATTCTCCTATGTTGTCTAT

GATCTCTTAGTGGCCTACTTACTCTTCTACATTGCCACCACTTATTTCCACAAGCTTCCA

TACCCATTTTCCTTCCTTGCTTGGCCAATCTATTGGGCCATCCAAGGCTGCATTCTCACT

GGTGTTTGGGTGATTGCTCATGAGTGTGGCCACCATGCCTTCAGCAAGTACCAACTTGTT

GATGACATGGTTGGTTTGACCCTTCACTCTTGTCTATTAGTTCCTTATTTCTCATGGAAA

ATCAGCCACCGCCGCCACCACTCCAACACCGGTTCCCTCGACCGCAACGAAGTGTTTGTC

CCAAAACCAAAATCAAAGGTATCATGGTATAACAAGTACATGAACAATCCACCAGGGAGG

GCTATCTCCCTCTTCATCACACTCACACTAGGATGGCCCTGATAATAATGACGTCAGAAT

TCTCGAGTCGGGGAAATGTGCGCGGAACCCCTATTTGTTTATTTTTCTAAATACATTCAA

ATATGTATCCGCTCATGAGACAATAACCCTGATAAATGCTTCAATAATATTGAAAAAGGA

AGAGTATGAGTATTCAACATTTCCGTGTCGCCCTTATTCCCTTTTTTGCGGCATTTTGCC

TTCCTGTTTTTGCTCACCCAGAAACGCTGGTGAAAGTAAAAGATGCTGAAGATCAGTTGG

GTGCACGAGTGGGTTACATCGAACTGGATCTCAACAGCGGTAAGATCCTTGAGAGTTTTC

GCCCCGAAGAACGTTTTCCAATGATGAGCACTTTTAAAGTTCTGCTATGTGGCGCGGTAT

TATCCCGTATTGACGCCGGGCAAGAGCAACTCGGTCGCCGCATACACTATTCTCAGAATG

ACTTGGTTGAGTACTCACCAGTCACAGAAAAGCATCTTACGGATGGCATGACAGTAGAGA

TTATGCAGTGCTGCCATACCATGAGTGATACACTGCGGCCAACTACTTCTGACACGATCG

GAGGACCGAAGGAGCTACGCTTTTTGCACACATGGGGGATCATGTAACTCGCCTGATCGT

GGGCACCGGAGCCTGAATGAAGCATACAAACGACGAGCCGGACCCACGATGCTGTAGCTA

ATGCACACGATGGCGCCACTATTAACCTGTGGGCGAAC

>31 fad2-1f

ACGAGGCTCTTTCAGGGTTCACATTCAACCCTCCATTCAGTGTTGGCCAACTCAAGAAAG

CAATTCCACCACATTGCTTTGAACGTTCTCTTTTCATATCATTCTCCTATGTTGTCTATG

ATCTCTTAGTGGCCTACTTACTCTTCTACATTGCCACCACTTATTTCCACAAGCTTCCAT

ACCCATTTTCCTTCCTTGCTTGGCCAATCTATTGGGCCATCCAAGGCTGCATTCTCACTG

GTGTTTGGGTGATTGCTCATGAGTGTGGCCACCATGCCTTCAGCAAGTACCAACTTGTTG

ATGACATGGTTGGTTTGACCCTTCACTCTTGTCTATTAGTTCCTTATTTCTCATGGAAAA

TCAGCCACCGCCGCCACCACTCCAACACCGGTTCCCTCGACCGCAACGAAGTGTTTGTCC

CAAAACCAAAATCAAAGGTATCATGGTATAACAAGTACATGAACAATCCACCAGGGAGGG

CTATCTCCCTCTTCATCACACTCACACTAGGATGGCCCTGATAATAATGACGTCAGAATT

CTCGAGTCGGGGAAATGTGCGCGGAACCCCTATTTGTTTATTTTTCTAAATACATTCAAA

TATGTATCCGCTCATGAGACAATAACCCTGATAAATGCTTCAATAATATTGAAAAAGGAA

GAGTATGAGTATTCAACATTTCCGTGTCGCCCTTATTCCCTTTTTTGCGGCATTTTGCCT

TCCTGTTTTTGCTCACCCAGAAACGCTGGTGAAAGTAAAAGATGCTGAAGATCAGTTGGG

TGCACGAGTGGGTTACATCGAACTGGATCTCAACAGCGGTAAGATCCTTGAGAGTTTTCG

CCCCGAAGAACGTTTTCCAATGATGAGCACTTTTAAAGTTCTGCTATGTGGCGCGGTATT

ATCCCGTATTGACGCCGGGCAAGAGCAACTCGGTCGCCGCATACACTATTCTCAGAATGA

CTTGGTTGAGTACTCACCAGTCACAGAAAAGCATCTTACGGATGGCATGACAGTAGAGAA

TTATGCAGTGCTGCCATAACCATGAGTGATACACTGCGGCCACTTACTCTGACACGATCG

GAGGACGAGGAGCTACCGCTTTTTGCACACATGGGGGATCATGTACCTCGCTTGATCGTG

GCACGGAGCTGATGAGCACACCACGACGAGCGTGAACCCCCGATGCCTGTAGCATTGCCA

CACGGTGGGCAACATATTAACTGGGCGACATACGTTACCTT

>32 fad2-1f

ACGAAGCTCTTTCAGGGTTCACATTCAAACCCTCCATTCAGTGTTGGCCAACTCAAGAAA

GCAATTCCACCACATTGCTTTGAACGTTCTCTTTTCATATCATTCTCATATGTTGTCTAT

GATCTCTTAATGGCCTACTTACTCTTCTACATTGCCACCACTTATTTCCACAAGCTTCCA

TACCCATTTTCCTTCCTTGCTTGGCCAATCTATTGGGCCATCCAAGGCTGCATTCTCACC

GGTGTTTGGGTGATTGCTCATGAGTGTGGCCACCATGCCTTCAGCAAGTACCAACTTGTT

GATGACATGGTTGGTTTGACCCTTCACTCTTGTCTATTAGTTCCTTATTTCTCATGGAAA

ATCAGCCACCGCCGCCACCACTCCAACACAGGTTCCCTCGACCGCGACGAAGTGTTTGTC

CCGAAACCAAAATCAAAGGTATCATGGTATAACAAGTACATGAACAATCCACCAGGGAGG

GCTATTTCCCTTTTCATCACACTCACACTAGGATGGCCCTGATAATAATGACGTCAGAAT

TCTCGAGTCGGGGAAATGTGCGCGGAACCCCTATTTGTTTATTTTTCTAAATACATTCAA

ATATGTATCCGCTCATGAGACAATAACCCTGATAAATGCTTCAATAATATTGAAAAAGGA

AGAGTATGAGTATTCAACATTTCCGTGTCGCCCTTATTCCCTTTTTTGCGGCATTTTGCC

TTCCTGTTTTTGCTCACCCAGAAACGCTGGTGAAAGTAAAAGATGCTGAAGATCAGTTGG

GTGCACGAGTGGGTTACATCGAACTGGATCTCAACAGCGGTAAGATCCTTGAGAGTTTTC

GCCCCGAAGAACGTTTTCCAATGATGAGCACTTTTAAAGTTCTGCTATGTGGCGCGGTAT

TATCCCGTATTGACGCCGGCAAGAGCAACTCGGTCGCCGCATACACTATTCTCAGAATGA

CTTGGTTGAGTACTCACCAGTCACAGAAAAGCATCTTACGATGGCATGACAGTAGAGAAT

TATGCAGTGCTGCCATACCATGAGTGATACACTGCGGCAACTTACTTCTGACACGATCGG

AGACGAGAGCTACCGCTTTTTGCCCAACATGGGGGATCATGTACTCGCTGGATCGTGGGA

ACGGAGCTGATGAAGCCTTCAACGGATACAGGCGTGAACCGAGTGCGTGACATGCCACGG

TTGGGCAACATTTACTGGGCGACCATCTTAACCC

>33 fad2-1f

ACGGAGCTCTTTCAGGGTTCACATTCAACCCTCCATTCAGTGTTGGCCAACTCAAGAAAG

CAATTCCACCACATTGCTTTGAACGTTCTCTTTTCATATCATTCTCCTATGTTGTCTATG

ATCTCTTAGTGGCCTACTTACTCTTCTACATTGCCACCACTTATTTCCACAAGCTTCCAT

ACCCATTTTCCTTCCTTGCTTGGCCAATCTATTGGGCCATCCAAGGCTGCATTCTCACCG

GTGTTTGGGTGATTGCTCATGAGTGTGGCCACCATGCCTTCAGCAAGTACCAACTTGTTG

ATGACATGGTTGGTTTGACCCTTCACTCTTGTCTATTAGTTCCTTATTTCTCATGGAAAA

TCAGCCACCGCCGCCACCACTCCAACACAGGTTCCCTCGACCGCGACGAAGTGTTTGTCC

CGAAACCAAAATCAAAGGTATCATGGTATAACAAGTACATGAACAATCCACCAGGGAGGG

CTATTTCCCTTTTCATCACACTCACACTAGGATGGCCCTGATAATAATGACGTCAGAATT

CTCGAGTCGGGGAAATGTGCGCGGAACCCCTATTTGTTTATTTTTCTAAATACATTCAAA

TATGTATCCGCTCATGAGACAATAACCCTGATAAATGCTTCAATAATATTGAAAAAGGAA

GAGTATGAGTATTCAACATTTCCGTGTCGCCCTTATTCCCTTTTTTGCGGCATTTTGCCT

TCCTGTTTTTGCTCACCCAGAAACGCTGGTGAAAGTAAAAGATGCTGAAGATCAGTTGGG

TGCACGAGTGGGTTACATCGAACTGGATCTCAACAGCGGTAAGATCCTTGAGAGTTTTCG

CCCCGAAGAACGTTTTCCAATGATGAGCACTTTTAAAGTTCTGCTATGTGGCGCGGTATT

ATCCCGTATTGACGCCGGGCAAGAGCAACTCGGTCGCCGCATACACTATTCTCAGAATGA

CTTGGTTGAGTACTCACCAGTCACAGAAAAGCATCTTACGGATGGCATGACAGTAGAGAA

TTATGCAGTGCTGCCATAACCATGAGTGATACACTGCGGCCAACTTACTTCTGACACGAT

CGGAGGACCGAAGGAGCTAACCGCTTTTTGCACACATGGGGGATCATGTACTCGCCTGAT

CGTGGACCGGAGCCTGAATGGAGCAACAACGACGAAGGCGTGACACTCACGAATGCTGTT

AGCATGGCCACACACCGTGTGGCGCGCAACCATATATTTACCCTCGGCCGACT

>34 fad2-1f

GGGTGAGCTCTTTCAGGTTCACATTCAAACCCTCCATTCAGTGTTGGCCAACTCAAGAAA

GCAATTCCACCACATTGCTTTGAACGTTCTCTTTTCATATCATTCTCCTATGTTGTCTAT

GATCTCTTAATGGCCTACTTACTCTTCTACATTGCCACCACTTATTTCCACCAGCTTCCA

TACCCATTTTCCTTCCTTGCTTGGCCAATCTATTGGGCCATCCAAGGCTGCATTCTCACT

GGTGTTTGGGTGATTGCTCATGAGTGTGGCCACCATGCCTTCAGCAAGTATCAACTTGTT

GATGACATGGTTGGTTTGATCCTTCACTCTTGTCTATTAGTCCCTTATTTCTCATGGAAA

ATCAGCCACCGCCACCTCCACTCCAACACCGGTTCCCTCGACCGCGACTAAGTGTTTGTC

CTGAAACCAAAATCAAAGGTATCACGGTATAACAAGTACATGAACAATCCACTAGAGAGG

GCTATTTCCCTTTTCATCACACTCACACTAGGATGGCCCTGATAATAATGACGTCAGAAT

TCTCGAGTCGGGGAAATGTGCGCGGAACCCCTATTTGTTTATTTTTCTAAATACATTCAA

ATATGTATCCGCTCATGAGACAATAACCCTGATAAATGCTTCAATAATATTGAAAAAGGA

AGAGTATGAGTATTCAACATTTCCGTGTCGCCCTTATTCCCTTTTTTGCGGCATTTTGCC

TTCCTGTTTTTGCTCACCCAGAAACGCTGGTGAAAGTAAAAGATGCTGAAGATCAGTTGG

GTGCACGAGTGGGTTACATCGAACTGGATCTCAACAGCGGTAAGATCCTTGAGAGTTTTC

GCCCCGAAGAACGTTTTCCAATGATGAGCACTTTTAAAGTTCTGCTATGTGGCGCGGTAT

TATCCCGTATTGACGCCCGGGCAAGAGCAACTCGGTCGCCGCATACACTATTCTCAGAAT

GACTTGGTTGAGTACTCACCAGTCACAGAAAAGCATCTTACGGATGGCATGACAGTAAGA

GAATTATGCAGTGCTGCCATAACCATGAGTGATACACTGCGGCCAACTTACTTCTGACAC

GATCGGAGGACGAGGAGCTAACCGCTTTTTGCACAACATGGGGATCATGTACTCGCCTTG

ATCGGTGGAACGGAGCTGATGGAAGCATACCAACGAACGAGCGTGACACCACGATGCTGT

AGCATGCACACGGTGGCCACTATAACTGGCGAACAACTACTCTAGCTTCCCGGCCACAT

>35 fad2-1f

ACAGAGCTCTTTCAGGGTTCACATTCAACCCTCCATTCAGTGTTGGCCAACTCAAGAAAG

CAATTCCACCACATTGCTTTGAACGTTCTCTTTTCATATCATTCTCCTATGTTGTCTATG

ATCTCTTAGTGGCCTACTTACTCTTCTACATTGCCACCACTTATTTCCACAAGCTTCCAT

ACCCATTTTCCTTCCTTGCTTGGCCAATCTATTGGGCCATCCAAGGCTGCATTCTCACTG

GTGTTTGGGTGATTGCTCATGAGTGTGGCCACCATGCCTTCAGCAAGTACCAACTTGTTG

ATGACATGGTTGGTTTGACCCTTCACTCTTGTCTATTAGTTCCTTATTTCTCATGGAAAA

TCAGCCACCGCCGCCACCACTCCAACACCGGTTCCCTCGACCGCAACGAAGTGTTTGTCC

CAAAACCAAAATCAAAGGTATCATGGTATAACAAGTACATGAACAATCCACCAGGGAGGG

CTATCTCCCTCTTCATCACACTCACACTAGGATGGCCCTGATAATAATGACGTCAGAATT

CTCGAGTCGGGGAAATGTGCGCGGAACCCCTATTTGTTTATTTTTCTAAATACATTCAAA

TATGTATCCGCTCATGAGACAATAACCCTGATAAATGCTTCAATAATATTGAAAAAGGAA

GAGTATGAGTATTCAACATTTCCGTGTCGCCCTTATTCCCTTTTTTGCGGCATTTTGCCT

TCCTGTTTTTGCTCACCCAGAAACGCTGGTGAAAGTAAAAGATGCTGAAGATCAGTTGGG

TGCACGAGTGGGTTACATCGAACTGGATCTCAACAGCGGTAAGATCCTTGAGAGTTTTCG

CCCCGAAGAACGTTTTCCAATGATGAGCACTTTTAAAGTTCTGCTATGTGGCGCGGTATT

ATCCCGTATTGACGCCCGGGCAAGAGCAACTCGGTCGCCGCATACACTATTCTCAGAATG

ACTTGGGTTGAGTACTCACCAGTCACAGAAAAGCATCTTACGGATGGCATGACAGTAGAG

AATTATGCAGTGCTGCCATAACCATGAGTGATACACTGCGGCGACTTACTTCTGACACGA

TCGGAGGACCGAAGGAGCTAACCGCTTTTTTGCACACATGGGGGGATCATGTAACTCGCC

TGATCGTGGACCGGAGCTGAATGAAGCCATACCGACGACGAGCGTGAACCACGATGCCGT

AGCAATGACAACAACGGTGGCCTAACTATTAACCTGGGCGAACACTACTCAAC

>36 fad2-1f

GGGGAGCTCTTTCATGGTTCACATTCAACCCTCCATTCAGTGTTGGCCAACTCAAGAAAG

CAATTCCACCACATTGCTTTGAACGTTCTCTTTTCATATCATTCTCCTATGTTGTCTATG

ATCTCTTAATGGCCTACTTACTCTTCTACATTGCCACCACTTATTTCCACCAGCTTCCAT

ACCCATTTTCCTTCCTTGCTTGGCCAATCTATTGGGCCATCCAAGGCTGCATTCTCACTG

GTGTTTGGGTGATTGCTCATGAGTGTGGCCACCATGCCTTCAGCAAGTATCAACTTGTTG

ATGACATGGTTGGTTTGATCCTTCACTCTTGTCTATTAGTCCCTTATTTCTCATGGAAAA

TCAGCCACCGCCACCTCCACTCCAACACCGGTTCCCTCGACCGCGACTAAGTGTTTGTCC

TGAAACCAAAATCAAAGGTATCACGGTATAACAAGTACATGAACAATCCACTAGAGAGGG

CTATTTCCCTTTTCATCACACTCACACTAGGATGGCCCTGATAATAATGACGTCAGAATT

CTCGAGTCGGGGAAATGTGCGCGGAACCCCTATTTGTTTATTTTTCTAAATACATTCAAA

TATGTATCCGCTCATGAGACAATAACCCTGATAAATGCTTCAATAATATTGAAAAAGGAA

GAGTATGAGTATTCAACATTTCCGTGTCGCCCTTATTCCCTTTTTTGCGGCATTTTGCCT

TCCTGTTTTTGCTCACCCAGAAACGCTGGTGAAAGTAAAAGATGCTGAAGATCAGTTGGG

TGCACGAGTGGGTTACATCGAACTGGATCTCAACAGCGGTAAGATCCTTGAGAGTTTTCG

CCCCGAAGAACGTTTTCCAATGATGAGCACTTTTAAAGTTCTGCTATGTGGCGCGGTATT

ATCCCGTATTGACGCCGGGCAAGAGCAACTCGGTCGCCGCATACACTATTCTCAGAATGA

CTTGGTTGAGTACTCACCAGTCACAGAAAAGCATCTTACGGATGGCATGACAGTAGAGAA

TTATGCAGTGCTGCATAACCATGAGTGATACACTGCGGCCACTTACTTCTGACACGATCG

GAGGACCGAGGAGCTAACCGCTTTTTTGCACAACATGGGGGGATCATGTAACTTCGCTGA

TCGTGGACCGGAGCTGAAATGAGCATACAACGACGGGCGGGACACACGATGCCTGTAGCA

TTGACCACACACGTTGCCCCAAAATATATATATACCTGGGCGCGAAACACACCTAAATC

>38 fad2-1f

AAGAAGCTCTTTCACGGTTCACATTCAACCCTCCATTCAGTGTTGGCCAACTCAAGAAAG

CAATTCCACCACATTGCTTTGAACGTTCTCTTTTCATATCATTCTCATATGTTGTCTATG

ATCTCTTAATGGCCTACTTACTCTTCTACATTGCCACCACTTATTTCCACAAGCTTCCAT

ACCCATTTTCCTTCCTTGCTTGGCCAATCTATTGGGCCATCCAAGGCTGCATTCTCACCG

GTGTTTGGGTGATTGCTCATGAGTGTGGCCACCATGCCTTCAGCAAGTACCAACTTGTTG

ATGACATGGTTGGTTTGACCCTTCACTCTTGTCTATTAGTTCCTTATTTCTCATGGAAAA

TCAGCCACCGCCGCCACCACTCCAACACAGGTTCCCTCGACCGCGACGAAGTGTTTGTCC

CGAAACCAAAATCAAAGGTATCATGGTATAACAAGTACATGAACAATCCACCAGGGAGGG

CTATTTCCCTTTTCATCACACTCACACTAGGATGGCCCTGATAATAATGACGTCAGAATT

CTCGAGTCGGGGAAATGTGCGCGGAACCCCTATTTGTTTATTTTTCTAAATACATTCAAA

TATGTATCCGCTCATGAGACAATAACCCTGATAAATGCTTCAATAATATTGAAAAAGGAA

GAGTATGAGTATTCAACATTTCCGTGTCGCCCTTATTCCCTTTTTTGCGGCATTTTGCCT

TCCTGTTTTTGCTCACCCAGAAACGCTGGTGAAAGTAAAAGATGCTGAAGATCAGTTGGG

TGCACGAGTGGGTTACATCGAACTGGATCTCAACAGCGGTAAGATCCTTGAGAGTTTTCG

CCCCGAAGAACGTTTTCCAATGATGAGCACTTTTAAAGTTCTGCTATGTGGCGCGGTATT

ATCCCGTATTGACGCCCGGCAAGAGCAACTCGGTCGCCGCATACACTATTCTCAGAATGA

CTTGGTTGAGTACTCACCAGTCACAGAAAAGCATCTTACGGATGGCATGACAGTAGAGAT

TATGCAGTGCTGCATACCATGAGTGATACACTGCGGCACTTACTCTGACACGATCGGAGG

ACCGAGAGCTAACCGCTTTTTGCACAACATGGGGGATCATGTAACTCGCTGATCGGTGGA

CGGAGCTGATGAGCATACAACGACGAAGCGTGACACCCCCGATGCGTAGCATTGACAACA

CGTGGCCCAACCTATATTATATCTGGGCCGGACACACTCCCTTATC

>39 fad2-1f

ACAAAGCTCTTTCAGGGTTCACATTCAACCCTCCATTCAGTGTTGGCCAACTCAAGAAAG

CAATTCCACCACATTGCTTTGAACGTTCTCTTTTCATATCATTCTCATATGTTGTCTATG

ATCTCTTAATGGCCTACTTACTCTTCTACATTGCCACCACTTATTTCCACAAGCTTCCAT

ACCCATTTTCCTTCCTTGCTTGGCCAATCTATTGGGCCATCCAAGGCTGCATTCTCACCG

GTGTTTGGGTGATTGCTCATGAGTGTGGCCACCATGCCTTCAGCAAGTACCAACTTGTTG

ATGACATGGTTGGTTTGACCCTTCACTCTTGTCTATTAGTTCCTTATTTCTCATGGAAAA

TCAGCCACCGCCGCCACCACTCCAACACAGGTTCCCTCGACCGCGACGAAGTGTTTGTCC

CGAAACCAAAATCAAAGGTATCATGGTATAACAAGTACATGAACAATCCACCAGGGAGGG

CTATTTCCCTTTTCATCACACTCACACTAGGATGGCCCTGATAATAATGACGTCAGAATT

CTCGAGTCGGGGAAATGTGCGCGGAACCCCTATTTGTTTATTTTTCTAAATACATTCAAA

TATGTATCCGCTCATGAGACAATAACCCTGATAAATGCTTCAATAATATTGAAAAAGGAA

GAGTATGAGTATTCAACATTTCCGTGTCGCCCTTATTCCCTTTTTTGCGGCATTTTGCCT

TCCTGTTTTTGCTCACCCAGAAACGCTGGTGAAAGTAAAAGATGCTGAAGATCAGTTGGG

TGCACGAGTGGGTTACATCGAACTGGATCTCAACAGCGGTAAGATCCTTGAGAGTTTTCG

CCCCGAAGAACGTTTTCCAATGATGAGCACTTTTAAAGTTCTGCTATGTGGCGCGGTATT

ATCCCGTATTGACGCCGGGCAAGAGCAACTCGGTCGCCGCATACACTATTCTCAGAATGA

CTTGGTTGAGTACTCACCAGTCACAGAAAAGCATCTTACGGATGGCATGACAGTAGAGAA

TTATGCAGTGCTGCCATAACCATGAGTGATACACTGCGCAACTTACTCTGACACGATCGG

AGACCGAGGAGCTAACCGCTTTGCACACATGGGGATCATGTACTCGCTGATCGTGGACGG

GAGCTGAATGAGCATACAACGACAGAGCGTGACACCGATGCTTAGCATGCAACACGTGCG

CGACTATATACTGGCGGAAACACCCTA

>40 fad2-1f

GCGGAAGCTCTTTCAGGGTTCACATTCAACCCTCCATTCAGTGTTGGCCAACTCAAGAAA

GCAATTCCACCACATTGCTTTGAACGTTCTCTTTTCATATCATTCTCCTATGTTGTCTAT

GATCTCTTAATGGCCTACTTACTCTTCTACATTGCCACCACTTATTTCCACCAGCTTCCA

TACCCATTTTCCTTCCTTGCTTGGCCAATCTATTGGGCCATCCAAGGCTGCATTCTCACT

GGTGTTTGGGTGATTGCTCATGAGTGTGGCCACCATGCCTTCAGCAAGTATCAACTTGTT

GATGACATGGTTGGTTTGATCCTTCACTCTTGTCTATTAGTCCCTTATTTCTCATGGAAA

ATCAGCCACCGCCACCTCCACTCCAACACCGGTTCCCTCGACCGCGACTAAGTGTTTGTC

CTGAAACCAAAATCAAAGGTATCACGGTATAACAAGTACATGAACAATCCACTAGAGAGG

GCTATTTCCCTTTTCATCACACTCACACTAGGATGGCCCTGATAATAATGACGTCAGAAT

TCTCGAGTCGGGGAAATGTGCGCGGAACCCCTATTTGTTTATTTTTCTAAATACATTCAA

ATATGTATCCGCTCATGAGACAATAACCCTGATAAATGCTTCAATAATATTGAAAAAGGA

AGAGTATGAGTATTCAACATTTCCGTGTCGCCCTTATTCCCTTTTTTGCGGCATTTTGCC

TTCCTGTTTTTGCTCACCCAGAAACGCTGGTGAAAGTAAAAGATGCTGAAGATCAGTTGG

GTGCACGAGTGGGTTACATCGAACTGGATCTCAACAGCGGTAAGATCCTTGAGAGTTTTC

GCCCCGAAGAACGTTTTCCAATGATGAGCACTTTTAAAGTTCTGCTATGTGGCGCGGTAT

TATCCCGTATTGACGCCGGGCAAGAGCAACTCGGTCGCCGCATACACTATTCTCAGAATG

ACTTGGGTTGAGTACTCACCAGTCACAGAAAAGCATCTTACGGATGGCATGACAGTAGAG

AATTATGCAGTGCTGCATACCATGAGTGATACACTGCGGCACTTACTCTGACACGATCGG

AGGACCGAGAGCTACCGCTTTTTGCACAACATGGGGGATCATGTACTCGCCTGATCGTGG

ACCGGAGCTGATTGAAGCATACAACGACGAGGCGTGAACCCACCGATGCCTGTAGCATTG

CACACACGTGGCCCAACACATATAAACTGGGCGGAACACATACTTTAA

>41 fad2-1f

GGGGGGAGCCTCTTTTCAGGGTTCACATTCAAACCCTCCATTCAGTGTTGGCCAACTCAA

GAAAGCAATTCCACCACATTGCTTTGAACGTTCTCTTTTCATATCATTCTCCTATGTTGT

CTATGATCTCTTAATGGCCTACTTACTCTTCTACATTGCCACCACTTATTTCCACCAGCT

TCCATACCCATTTTCCTTCCTTGCTTGGCCAATCTATTGGGCCATCCAAGGCTGCATTCT

CACTGGTGTTTGGGTGATTGCTCATGAGTGTGGCCACCATGCCTTCAGCAAGTATCAACT

TGTTGATGACATGGTTGGTTTGATCCTTCACTCTTGTCTATTAGTCCCTTATTTCTCATG

GAAAATCAGCCACCGCCACCTCCACTCCAACACCGGTTCCCTCGACCGCGACTAAGTGTT

TGTCCTGAAACCAAAATCAAAGGTATCACGGTATAACAAGTACATGAACAATCCACTAGA

GAGGGCTATTTCCCTTTTCATCACACTCACACTAGGATGGCCCTGATAATAATGACGTCA

GAATTCTCGAGTCGGGGAAATGTGCGCGGAACCCCTATTTGTTTATTTTTCTAAATACAT

TCAAATATGTATCCGCTCATGAGACAATAACCCTGATAAATGCTTCAATAATATTGAAAA

AGGAAGAGTATGAGTATTCAACATTTCCGTGTCGCCCTTATTCCCTTTTTTGCGGCATTT

TGCCTTCCTGTTTTTGCTCACCCAGAAACGCTGGTGAAAGTAAAAGATGCTGAAGATCAG

TTGGGTGCACGAGTGGGTTACATCGAACTGGATCTCAACAGCGGTAAGATCCTTGAGAGT

TTTCGCCCGAAGAACGTTTTCCAATGATGAGCACTTTTAAAGTTCTGCTATGTGGCGCGT

ATATCCCGTATTGACGCCGGGCAAGAGCACTCGGTCGCCGCATACACTATTCTCAGAATG

ACTTTGTGAGTACCTCACCAGTCACAGAAAAGCATTCTTACGATGCATGACAGTAGAAAA

TTATGCAGTGCTGCCATACATGAGGTGATAACACTGGCGGCCAACTACCTTCTGACAACG

ATCCGAGAGACCGATGACCTACCGCTTTTGCACAACATGGGAATCAGGTACTCGCCTGAT

CGTTGGCAACGGACCTGAATGGACACCCATAATCCAAACGTCCGT

>42 fad2-1f

CGGGGAGCTCGTTACGGTTCACATTCAACCCTCCATTCAGTGTTGGCCAACTCAAGAAAG

CAATTCCACCACATTGCTTTGAACGTTCTCTTTTCATATCATTCTCCTATGTTGTCTATG

ATCTCTTAATGGCCTACTTACTCTTCTACATTGCCACCACTTATTTCCACCAGCTTCCAT

ACCCATTTTCCTTCCTTGCTTGGCCAATCTATTGGGCCATCCAAGGCTGCATTCTCACTG

GTGTTTGGGTGATTGCTCATGAGTGTGGCCACCATGCCTTCAGCAAGTATCAACTTGTTG

ATGACATGGTTGGTTTGATCCTTCACTCTTGTCTATTAGTCCCTTATTTCTCATGGAAAA

TCAGCCACCGCCACCTCCACTCCAACACCGGTTCCCTCGACCGCGACTAAGTGTTTGTCC

TGAAACCAAAATCAAAGGTATCACGGTATAACAAGTACATGAACAATCCACTAGAGAGGG

CTATTTCCCTTTTCATCACACTCACACTAGGATGGCCCTGATAATAATGACGTCAGAATT

CTCGAGTCGGGGAAATGTGCGCGGAACCCCTATTTGTTTATTTTTCTAAATACATTCAAA

TATGTATCCGCTCATGAGACAATAACCCTGATAAATGCTTCAATAATATTGAAAAAGGAA

GAGTATGAGTATTCAACATTTCCGTGTCGCCCTTATTCCCTTTTTTGCGGCATTTTGCCT

TCCTGTTTTTGCTCACCCAGAAACGCTGGTGAAAGTAAAAGATGCTGAAGATCAGTTGGG

TGCACGAGTGGGTTACATCGAACTGGATCTCAACAGCGGTAAGATCCTTGAGAGTTTTCG

CCCCGAAGAACGTTTTCCAATGATGAGCACTTTTAAAGTTCTGCTATGTGGCGCGGTATT

ATCCCGTATTGACGCCCGGGCAAGAGCAACTCGGTCGCCGCATACACTATTCTCAGAATG

ACTTGGTTGAGTACTCACAGTCACAGAAAAGCATCTTACGGATGGCATGACAGTAAGAGA

ATTATGCAGTGCTGCCATACATGAGTGATACACTGCGGCACTTACTTCTGACACGATCGG

AGGACGAGAGCTACGCTTTTTGCACAACATGGGGATCATGTACTCGCCTTGATCGGTGGA

CGAGCTGATGAGCCATACACGACGAGCGTGACACACCGATGCTGTAGCATGGCACACGTT

GGCCTACCATTGACCTTGGCGAACATACTACTCCTGAGGCTCTTTTC

>43 fad2-1f

ACGAAGCTCTTTCAGGGTTCACATTCAAACCCTCCATTCAGTGTTGGCCAACTCAAGAAA

GCAATTCCACCACATTGCTTTGAACGTTCTCTTTTCATATCATTCTCCTATGTTGTCTAT

GATCTCTTAGTGGCCTACTTACTCTTCTACATTGCCACCACTTATTTCCACAAGCTTCCA

TACCCATTTTCCTTCCTTGCTTGGCCAATCTATTGGGCCATCCAAGGCTGCATTCTCACT

GGTGTTTGGGTGATTGCTCATGAGTGTGGCCACCATGCCTTCAGCAAGTACCAACTTGTT

GATGACATGGTTGGTTTGACCCTTCACTCTTGTCTATTAGTTCCTTATTTCTCATGGAAA

ATCAGCCACCGCCGCCACCACTCCAACACCGGTTCCCTCGACCGCAACGAAGTGTTTGTC

CCAAAACCAAAATCAAAGGTATCATGGTATAACAAGTACATGAACAATCCACCAGGGAGG

GCTATCTCCCTCTTCATCACACTCACACTAGGATGGCCCTGATAATAATGACGTCAGAAT

TCTCGAGTCGGGGAAATGTGCGCGGAACCCCTATTTGTTTATTTTTCTAAATACATTCAA

ATATGTATCCGCTCATGAGACAATAACCCTGATAAATGCTTCAATAATATTGAAAAAGGA

AGAGTATGAGTATTCAACATTTCCGTGTCGCCCTTATTCCCTTTTTTGCGGCATTTTGCC

TTCCTGTTTTTGCTCACCCAGAAACGCTGGTGAAAGTAAAAGATGCTGAAGATCAGTTGG

GTGCACGAGTGGGTTACATCGAACTGGATCTCAACAGCGGTAAGATCCTTGAGAGTTTTC

GCCCCGAAGAACGTTTTCCAATGATGAGCACTTTTAAAGTTCTGCTATGTGGCGCGGTAT

TATCCCGTATTGACGCCCGGGCAAGAGCAACTCGGTCGCCGCATACACTATTCTCAGAAT

GACTTGGTTGAGTACTCACCAGTCACAGAAAAGCATCTTACGGATGGCATGACAGTAGAG

AATTATGCAGTGCTGCCATACCATGAGTGATACACTGCGGCAACTTACTTCTGACACGAT

CGGAGGACGAGGAGCTACCGCTTTTTGCACAACATGGGGGATCATGTACTCGCCTGATCG

TGGACCGGAGCTGAATGAGCATACTAACGACGAGCGTGACACCACGATGCTGTACATGCA

CACGTTGCCACTATTTACTGGGGAAAACACTTATCTCAGCCTTCCC

>44 fad2-1f

AGAAAGAAGGCTCTTTCAGGGTTCACATTCAAACCCTCCATTCAGTGTTGGCCAACTCAA

GAAAGCAATTCCACCACATTGCTTTGAACGTTCTCTTTTCATATCATTCTCCTATGTTGT

CTATGATCTCTTAATGGCCTACTTACTCTTCTACATTGCCACCACTTATTTCCACCAGCT

TCCATACCCATTTTCCTTCCTTGCTTGGCCAATCTATTGGGCCATCCAAGGCTGCATTCT

CACTGGTGTTTGGGTGATTGCTCATGAGTGTGGCCACCATGCCTTCAGCAAGTATCAACT

TGTTGATGACATGGTTGGTTTGATCCTTCACTCTTGTCTATTAGTCCCTTATTTCTCATG

GAAAATCAGCCACCGCCACCTCCACTCCAACACCGGTTCCCTCGACCGCGACTAAGTGTT

TGTCCTGAAACCAAAATCAAAGGTATCACGGTATAACAAGTACATGAACAATCCACTAGA

GAGGGCTATTTCCCTTTTCATCACACTCACACTAGGATGGCCCTGATAATAATGACGTCA

GAATTCTCGAGTCGGGGAAATGTGCGCGGAACCCCTATTTGTTTATTTTTCTAAATACAT

TCAAATATGTATCCGCTCATGAGACAATAACCCTGATAAATGCTTCAATAATATTGAAAA

AGGAAGAGTATGAGTATTCAACATTTCCGTGTCGCCCTTATTCCCTTTTTTGCGGCATTT

TGCCTTCCTGTTTTTGCTCACCCAGAAACGCTGGTGAAAGTAAAAGATGCTGAAGATCAG

TTGGGTGCACGAGTGGGTTACATCGAACTGGATCTCAACAGCGGTAAGATCCTTGAGAGT

TTTCGCCCCGAAGAACGTTTTCCAATGATGAGCACTTTTAAAGTTCTGCTATGTGGCGCG

GTATTATCCCGTATTGACGCCCGGGCAAGAGCAACTCGGTCGCCGCATACACTATTCTCA

GATGACTTGGGTTGAGTACTCACCAGTCACAGAAAAGCATCTTACGGATGGCATGACAGT

AGAGATTATGCAGTGCTGCCATAACCATGAGTGATACACTGCGCAACTTACTTCTGACAC

GATCGGAGGACGAAGAGCTACCGCTTTTTGCACAACATGGGGGATCATGTACTCGCCTGG

ATCGTGGACGAGCTGATGAAGCAACCTAACGACGAGCGTGAACCACGATGCCTGTAGCAT

GCCACACGTTTGCCACCTATTACTGGGCAACACTCTTAATCTCCTTAGCTTTTCG

>45 fad2-1f

GGCCTGAGCTCTTTCAGGGTTCACATTCAAACCCTCCATTCAGTGTTGGCCAACTCAAGA

AAGCAATTCCACCACATTGCTTTGAACGTTCTCTTTTCATATCATTCTCCTATGTTGTCT

ATGATCTCTTAATGGCCTACTTACTCTTCTACATTGCCACCACTTATTTCCACCAGCTTC

CATACCCATTTTCCTTCCTTGCTTGGCCAATCTATTGGGCCATCCAAGGCTGCATTCTCA

CTGGTGTTTGGGTGATTGCTCATGAGTGTGGCCACCATGCCTTCAGCAAGTATCAACTTG

TTGATGACATGGTTGGTTTGATCCTTCACTCTTGTCTATTAGTCCCTTATTTCTCATGGA

AAATCAGCCACCGCCACCTCCACTCCAACACCGGTTCCCTCGACCGCGACTAAGTGTTTG

TCCTGAAACCAAAATCAAAGGTATCACGGTATAACAAGTACATGAACAATCCACTAGAGA

GGGCTATTTCCCTTTTCATCACACTCACACTAGGATGGCCCTGATAATAATGACGTCAGA

ATTCTCGAGTCGGGGAAATGTGCGCGGAACCCCTATTTGTTTATTTTTCTAAATACATTC

AAATATGTATCCGCTCATGAGACAATAACCCTGATAAATGCTTCAATAATATTGAAAAAG

GAAGAGTATGAGTATTCAACATTTCCGTGTCGCCCTTATTCCCTTTTTTGCGGCATTTTG

CCTTCCTGTTTTTGCTCACCCAGAAACGCTGGTGAAAGTAAAAGATGCTGAAGATCAGTT

GGGTGCACGAGTGGGTTACATCGAACTGGATCTCAACAGCGGTAAGATCCTTGAGAGTTT

TCGCCCCGAAGAACGTTTTCCAATGATGAGCACTTTTAAAGTTCTGCTATGTGGCGCGGT

ATTATCCCGTATTGACGCCGGGCAAGAGCAACTCGGTCGCCGCATACACTATTCTCAGAA

TGACTTGGTTGAGTACTCACCAGTCACAGAAAAGCATCTTACGGATGGCATGACAGTAGA

GATTATGCAGTGCTGCCATACCATGAGTGATACACTGCGGCCACTTACTTCTGACACGAT

CGGAGGACCGAGGAGCTACCGCTTTTTGCACACATGGGGATCATGTACCTCGCCTTGATC

GTGGACGGGGCTGATGAGCCTTCACGACGAGCGTGACACCACGATGCCTGTAGCATTGCA

CCACGGGTGCCTAACATATTAACTGGGCGAACTACCTTTATCCTCTCAGGCCTTCCCG

>46 fad2-1f

AAGGGAGCTCTTTCAGGGTTCACATTCAACCCTCCATTCAGTGTTGGCCAACTCAAGAAA

GCAATTCCACCACATTGCTTTGAACGTTCTCTTTTCATATCATTCTCCTATGTTGTCTAT

GATCTCTTAATGGCCTACTTACTCTTCTACATTGCCACCACTTATTTCCACCAGCTTCCA

TACCCATTTTCCTTCCTTGCTTGGCCAATCTATTGGGCCATCCAAGGCTGCATTCTCACT

GGTGTTTGGGTGATTGCTCATGAGTGTGGCCACCATGCCTTCAGCAAGTATCAACTTGTT

GATGACATGGTTGGTTTGATCCTTCACTCTTGTCTATTAGTCCCTTATTTCTCATGGAAA

ATCAGCCACCGCCACCTCCACTCCAACACCGGTTCCCTCGACCGCGACTAAGTGTTTGTC

CTGAAACCAAAATCAAAGGTATCACGGTATAACAAGTACATGAACAATCCACTAGAGAGG

GCTATTTCCCTTTTCATCACACTCACACTAGGATGGCCCTGATAATAATGACGTCAGAAT

TCTCGAGTCGGGGAAATGTGCGCGGAACCCCTATTTGTTTATTTTTCTAAATACATTCAA

ATATGTATCCGCTCATGAGACAATAACCCTGATAAATGCTTCAATAATATTGAAAAAGGA

AGAGTATGAGTATTCAACATTTCCGTGTCGCCCTTATTCCCTTTTTTGCGGCATTTTGCC

TTCCTGTTTTTGCTCACCCAGAAACGCTGGTGAAAGTAAAAGATGCTGAAGATCAGTTGG

GTGCACGAGTGGGTTACATCGAACTGGATCTCAACAGCGGTAAGATCCTTGAGAGTTTTC

GCCCCGAAGAACGTTTTCCAATGATGAGCACTTTTAAAGTTCTGCTATGTGGCGCGGTAT

TATCCCGTATTGACGCCCGGGCAAGAGCAACTCGGTCGCCGCATACACTATTCTCAGAAT

GACTTGGTTGAGTACTCACCAGTCACAGAAAAGCATCTTACGGATGGCATGACAGTAGAG

ATTATGCAGTGCTGCCATACCATGAGTGATACACTGCGGCCAACTTACTTCTGACACGAT

CGGAGGACCGAAGGAGCTACGCTTTTTTGCACAACATGGGGGATCATGTACTCGCCTGAT

CGTGGACGGAGCTGATGAGCATACTCGACGAGCGGTGACACCACGATGCCTGTACATTGG

CACACGTGGCCACCTTTACCTGGCCAAACTACTTAACTCTAAGACTTTCCGGGATCCA

>47 fad2-1f

GGGGAAGCTCGTTCAGGGTTCACATTCAACCCTCCATTCAGTGTTGGCCAACTCAAGAAA

GCAATTCCACCACATTGCTTTGAACGTTCTCTTTTCATATCATTCTCCTATGTTGTCTAT

GATCTCTTAATGGCCTACTTACTCTTCTACATTGCCACCACTTATTTCCACCAGCTTCCA

TACCCATTTTCCTTCCTTGCTTGGCCAATCTATTGGGCCATCCAAGGCTGCATTCTCACT

GGTGTTTGGGTGATTGCTCATGAGTGTGGCCACCATGCCTTCAGCAAGTATCAACTTGTT

GATGACATGGTTGGTTTGATCCTTCACTCTTGTCTATTAGTCCCTTATTTCTCATGGAAA

ATCAGCCACCGCCACCTCCACTCCAACACCGGTTCCCTCGACCGCGACTAAGTGTTTGTC

CTGAAACCAAAATCAAAGGTATCACGGTATAACAAGTACATGAACAATCCACTAGAGAGG

GCTATTTCCCTTTTCATCACACTCACACTAGGATGGCCCTGATAATAATGACGTCAGAAT

TCTCGAGTCGGGGAAATGTGCGCGGAACCCCTATTTGTTTATTTTTCTAAATACATTCAA

ATATGTATCCGCTCATGAGACAATAACCCTGATAAATGCTTCAATAATATTGAAAAAGGA

AGAGTATGAGTATTCAACATTTCCGTGTCGCCCTTATTCCCTTTTTTGCGGCATTTTGCC

TTCCTGTTTTTGCTCACCCAGAAACGCTGGTGAAAGTAAAAGATGCTGAAGATCAGTTGG

GTGCACGAGTGGGTTACATCGAACTGGATCTCAACAGCGGTAAGATCCTTGAGAGTTTTC

GCCCCGAAGAACGTTTTCCAATGATGAGCACTTTTAAAGTTCTGCTATGTGGCGCGGTAT

TATCCCGTATTGACGCCCGGGCAAGAGCAACTCGGTCGCCGCATACACTATTCTCAGAAT

GACTTGGGTTGAGTACTCACCAGTCACAGAAAAGCATCTTACGGATGGCATGACAGTAGA

GAATTATGCAGTGCTGCCATAACCATGAGTGATACACTGCGCCAACTTACTTCTGACACG

ATCGGAGGACCGAGGAGCTACCGCTTTTTGCACAACATGGGGGATCATGTACTCGCCTTG

ATCGTGGACGGGGCTGATGAGCATACTACGACGAGCGGACCCACGAATGCTTAGCATTGC

ACACGTGCGCCAACTATTTAACTGGGCGACCTACTAACCCTAAGCTCTCTCCGCGA

>48 fad2-1f

ACGGGAGCTCTTTCAGGGTTCACATTCAACCCTCCATTCAGTGTTGGCCAACTCAAGAAA

GCAATTCCACCACATTGCTTTGAACGTTCTCTTTTCATATCATTCTCCTATGTTGTCTAT

GATCTCTTAGTGGCCTACTTACTCTTCTACATTGCCACCACTTATTTCCACAAGCTTCCA

TACCCATTTTCCTTCCTTGCTTGGCCAATCTATTGGGCCATCCAAGGCTGCATTCTCACT

GGTGTTTGGGTGATTGCTCATGAGTGTGGCCACCATGCCTTCAGCAAGTACCAACTTGTT

GATGACATGGTTGGTTTGACCCTTCACTCTTGTCTATTAGTTCCTTATTTCTCATGGAAA

ATCAGCCACCGCCGCCACCACTCCAACACCGGTTCCCTCGACCGCAACGAAGTGTTTGTC

CCAAAACCAAAATCAAAGGTATCATGGTATAACAAGTACATGAACAATCCACCAGGGAGG

GCTATCTCCCTCTTCATCACACTCACACTAGGATGGCCCTGATAATAATGACGTCAGAAT

TCTCGAGTCGGGGAAATGTGCGCGGAACCCCTATTTGTTTATTTTTCTAAATACATTCAA

ATATGTATCCGCTCATGAGACAATAACCCTGATAAATGCTTCAATAATATTGAAAAAGGA

AGAGTATGAGTATTCAACATTTCCGTGTCGCCCTTATTCCCTTTTTTGCGGCATTTTGCC

TTCCTGTTTTTGCTCACCCAGAAACGCTGGTGAAAGTAAAAGATGCTGAAGATCAGTTGG

GTGCACGAGTGGGTTACATCGAACTGGATCTCAACAGCGGTAAGATCCTTGAGAGTTTTC

GCCCCGAAGAACGTTTTCCAATGATGAGCACTTTTAAAGTTCTGCTATGTGGCGCGGTAT

TATCCCGTATTGACGCCCGGGCAAGAGCAACTCGGTCGCCGCATACACTATTCTCAGAAT

GACTTGGTTGAGTACTCACCAGTCACAGAAAAGCATCTTACGGATGGCATGACAGTAAGA

GAATTATGCAGTGCTGCCATAACCATGAGTGATACACTGCGGCCAACTTACTCTGACACG

ATCGGAGGACCGAGGAGCTAACCGCTTTTTTGCACAACATGGGGATCATGTAACTCGCCT

GATCGTGGACGGGCTGATGAAGCCAACAAACGACGGCCGTGACATCGATGCCTGTAGCAT

TGACACACACGTTTGCGCAACTATTATACTTGGGCGAAACCACCCTAATC
