# Supplementary material for: Mutagenesis of FAD2 genes in peanut with CRISPR/Cas9 based gene editing
Source: BMC Biotechnol. 2019 Apr 29;19:24. doi: 10.1186/s12896-019-0516-8 (PMC6489235; doi:10.1186/s12896-019-0516-8)
Supplement: Supplementary file 2 — 100 FAD2 gene sequences isolated from hairy root DNAs transformed with gRNA6 in Exp-1516. (DOCX 14 kb) [file 12896_2019_516_MOESM2_ESM.docx]

Additional file 2

100 FAD2 gene sequences isolated from hairy root DNAs transformed with gRNA6 in Exp-1516.

>1 1_FAD2_Plate_Plate 01_A01

TTCAAACCCTCCATTCAGTGTTGGCCAACTCAAGAAAGCAATTCCNCCACATTGCTTTGAACGTTCTCTTTTCATATCATTCTCATATGTTGTCTATGATCTCTTAATGGCCTACTTACTCTTCTACATTGCCACCACTTATTTCCACAAGCTTCCATACCCATTTTCCTTCCTTGCTTGGCCAATCTATTGGGCCATCCAAGGCTGCATTCTCACCGGTGTTTGGGTGATTGCTCATGAGTGTGGCCACCATGCCTTCAGCAAGTACCAACTTGTTGATGACATGGTTGGTTTGACCCTTCACTCTTGTCTATTAGTTCCTTATTTCTCATGGAAAATCAGCCACCGCCGCCACCACTCCAACACAGGTTCCCTCGACCGCGACGAAGTGTTTGTCCCGAAACCAAAATCAAAGGTATCATGGTATAACAAGTACATGAACAATCCACCAGGGAGGGCTATTTCCCTTTTCATCACACTCACACTAGGATGGCCAAT

>2_FAD2_Plate_Plate 01_A02

TNCNACCCTCCATTCAGTGTTGGCCAACTCAAGAAAGCAATTCCACCACATTGCTTTGAACGTTCTCTTTTCATATCATTCTCCTATGTTGTCTATGATCTCTTAGTGGCCTACTTACTCTTCTACATTGCCACCACTTATTTCCACAAGCTTCCATACCCATTTTCCTTCCTTGCTTGGCCAATCTATTGGGCCATCCAAGGCTGCATTCTCACTGGTGTTTGGGTGATTGCTCATGAGTGTGGCCACCATGCCTTCAGCAAGTACCAACTTGTTGATGACATGGTTGGTTTGACCCTTCACTCTTGTCTATTAGTTCCTTATTTCTCATGGAAAATCAGCCACCGCCGCCACCACTCCAACACCGGTTCCCTCGACCGCAACGAAGTGTTTGTCCCAAAACCAAAATCAAAGGTATCATGGTATAACAAGTACATGAACAATCCACCAGGGAGGGCTATCTCCCTCTTCATCACACTCACNCTAGGANGGNCAA-

>3_FAD2_Plate_Plate 01_A03

TTCNNNNNNTCCATTCAGTGTTGGCCAACTCAAGAAAGCAATTCCACCCCATTGCTTTGAACGTTCTCTTTTCATATCATTCTCATATGTTGTCTATGATCTCTTAATGGCCTACTTACTCTTCTACATTGCCACCACTTATTTCCACAAGCTTCCATACCCATTTTCCTTCCTTGCTTGGCCAATCTATTGGGCCATCCAAGGCTGCATTCTCGCCGGTGTTTGGGTGATTGCTCATGAGTGTGGCCACCATGCCTTCAGCAAGTACCAACTTGTTGATGACATGGTTGGTTTGACCCTTCACTCTTGTCTATTAGTTCCTTATTTCTCATGGAAAATCAGCCACCGCCGCCACCACTCCAACACAGGTTCCCTCAGACCGCGACAAAGTGTTTGTCCCGAAACCAAAATCAAAGGTATCATGGTATAACAAGTACATGAACAATCCACCAGGGAGGGCTATTTCCCTTTTCATCACACTCACACTAGGATGGCCAAT

>8_FAD2_Plate_Plate 01_A08

TTCAACCCTCCATTCAGTGTTGNNCAACTCAAGAAAGCAATTCCACCACATTGCTTTGAACGTTCTCTTTTCATATCATTCTCATATGTTGTCTATGATCTCTTAATGGCCTACTTACTCTTCTACATTGCCACCACTTATTTCCACAAGCTTCCATACCCATTTTCCTTCCTTGCTTGGCCAATCTATTGGGCCATCCAAGGCTGCATTCTCACCGGTGTTTGGGTGATTGCTCATGAGTGTGGCCACCATGCCTTCAGCAAGTACCAACTTGTTGATGACATGGTTGGTTTGACCCTTCACTCTTGTCTATTAGTTCCTTATTTCTCATGGAAAATCAGCCACCGCCGCCACCACTCCAACACAGGTTCCCTCGACCGCGACGAAGTGTTTGTCCCGAAACCAAAATCAAAGGTATCATGGTATAACAAGTACATGAACAATCCACCAGGGAGGGCTATTTCCCTTTTCATCACACTCACACTAGGATGGCCAAT

>10_FAD2_Plate_Plate 01_A10

TTCNAACCCTCCATTCAGTGTTGGCCAACTCAAGAAAGCAATTCCACCACATTGCTTTGAACGTTCTCTTTTCATATCATTCTCATATGTTGTCTATGATCTCTTAATGGCCTACTTACTCTTCTACATTGCCACCACTTATTTCCACAAGCTTCCATACCCATTTTCCTTCCTTGCTTGGCCAATCTATTGGGCCATCCAAGGCTGCATTCTCACCGGTGTTTGGGTGATTGCTCATGAGTGTGGCCACCATGCCTTCAGCAAGTACCAACTTGTTGATGACATGGTTGGTTTGACCCTTCACTCTTGTCTATTAGTTCCTTATTTCTCATGGAAAATCAGCCACCGCCGCCACCACTCCAACACAGGTTCCCTCGACCGCGACGAGGTGTTCGTCCCGAAACCAAAATCAAAGGTATCATGGTATAACAAGTACATGAACAATCCACCAGGGAGGGCTATTTCCCTTTTCATCACACTCACACTAGGATGGCCAAT

>11_FAD2_Plate_Plate 01_A11

TTCAAGCCCTCCATTCAGTGTTGGCCAACTCAAGAAAGCAATTCCACCACATTGCTTTAAACGTTCTCTTTTCATATCATTCTCCTATGTTGTCTATGATCTCTTAATGGCCTACTTACTCTTCTACATTGCCACCACTTATTTCCACCAGCTTCCATACCCATTTTCCTTCCTTGCTTGGCCAATCTATTGGGCCATCCAAGGCTGCATTCTCACTGGTGTTTGGGTGATTGCTCATGAGTGTGGCCACCATGCCTTCAGCAAGTATCAACTTGTTGATGACATGGTTGGTTTGATCCTTCACTCTTGTCTATTAGTCCCTTATTTCTCATGGAAAATCAGCCACCGCCACCTCCACTCCAACACCGGTTCCCTCGACCGCGACTAAGTGTTTGTCCTGAAACCAAAATCAAAGGTATCACGGTATAACAAGTACATGAACAATCCACTAGAGAGGGCTATTTCCCTTTTCATCACACTCACACTAGGATGGCCAAT

>12_FAD2_Plate_Plate 01_A12

TTCNAACCCTCCATTCAGTGTTGGCCAACTCAAGAAAGCAATTCCACCACATTGCTTTGAACGTTCTCTTTTCATACCATTCTCCTATGTTGTCTATGATCTCTTAGTGGCCTACTTACTCTTCTACATTGCCACCACTTATTTCCACAAGCTTCCATACCCATTTTCCTTCCTTGCTTGGCCAATCTATTGGGCCATCCAAGGCTGCATTCTCACTGGTGTTTGGGTGATTGCTCATGGGTGTGGCCACCATGCCTTCAGCAAGTACCAACTTGTTGATGACATGGTTGGTTTGACCCTTCACTCTTGTCTATTAGTTCCTTATTTCTCATGGAAAATCAGCCACCGCCGCCACCACTCCAACACCGGTTCCCTCGACCGCAACGAAGTGTTTGTCCCAAAACCAAAATCAAAGGTATCATGGTATAACAAGTACATGAACAATCCACCAGGGAGGGCTATCTCCCTCTTCATCACACTCACACTAGGATGGCCAAT

>16_FAD2_Plate_Plate 01_B04

TTNNAACCCTCCATTCAGTGTTGGCCAACTCAAGAAAGCAATTCCACCACATTGCTTTGAACGTTCTCTTTTCATATCATTCTCATATGTTGTCTATGATCTCTTAATGGCCTACTTACTCTTCTACATTGCCACCACTTATTTCCACAAGCTTCCATACCCATTTTCCTTCCTTGCTTGGCCAATCTATTGGGCCATCCAAGGCTGCATTCTCACCGGTGTTTGGGTGATTGCTCATGAGTGTGGCCACCATGCCTTCAGCAAGTACCAACTTGTTGATGACATGGTTGGTTTGACCCTTCACTCTTGTCTATTAGTTCCTTATTTCTCATGGAAAATCAGCCACCGCCGCCACCACTCCAACACAGGTTCCCTCAGACCGCGACGAAGTGTTTGTCCCGAAACCAAAATCAAAGGTATCATGGTATAACAAGTACATGAACAATCCACCAGGGAGGGCTATTTCCCTTTTCATCACACTCACACTAGGATGGCCAAT

>17_FAD2_Plate_Plate 01_B05

TTNNAACCCTCCATTCAGTGTTGGCCAACTCAAGAAAGCAATTCCGCCGCATTGCTTTGAACGTTCTCTTTTCATATCATTCTCCTATGTTGTCTATGATCTCTTAATGGCCTACTTACTCTTCTACATTGCCACCACTTATTTCCACCAGCTTCCATACCCATTTTCCTTCCTTGCTTGGCCAATCTATTGGGCCATCCAAGGCTGCATTCTCACTGGTGTTTGGGTGATTGCTCATGAGTGTGGCCACCATGCCTTCAGCAAGTATCAACTTGTTGATGACATGGTTGGTTTGATCCTTCACTCTTGTCTATTAGTCCCTTATTTCTCATGGAAAATCAGCCACCGCCACCTCCACTCCAACACCGGTTCCCTCGACCGCGACTAAGTGTTTGTCCTGAAACCAAAATCAAAGGTATCACGGTATAACAAGTACATGAACAATCCACTAGAGAGGGCNATTTCCCTTTTCATCACACTCACACTAGGATGGCCAAT

>28_FAD2_Plate_Plate 01_C04

TNCNNNNNCTATTNNNNNNNGCCCCNGAANAAAGCCACACNANNNNCNNGNNTTGAACANNNCCTTTTCATATNACTCNCATATGTNGCCTATGATCTCTTAATGGCCTACTTACCCTTCTACATTGCCACCACTTATTTCCACAAGCTTCCATACCCATTTTCCTTCCTTGGTTGGCCAATCTATTGGGCCATCCCAGGCTGCATTTTCACCGGTGTTTGGGTGATTGCTCATGANTGTGGCCACCATGCCTTCAGCAAGTACCAACTTGNTGATGACATGGTTGGTTTGACCCTTCACTCTTGTCTATTAGTNCCTTATTTCTCATGGAAAATCAGCCCCCNNCGCCCCCACTCCCACACAGGTTCCCTCGACCGCGACGAAGCGTTTGTCCCGAAACCAAAATCCAAGGNATCATGGNATAACNAGTATATGAACAATCCACCAGGGAGGCCTATTTCCTGATTCATCACANTC-CCNTTNTATGGTCAN

>36_FAD2_Plate_Plate 01_C12

TTCAAACCCTCCATTCAGTGTTGGCCAACTCAAGAAAGCAATTCCACCACATTGCTTTGAACGTTCTCTTTTCATATCATTCTCATATGTCGTCTATGATCTCTTAATGGCCTACTTACTCTTCTACATTGCCACCACTTATTTCCACAAGCTTCCATACCCATTTTCCTTCCTTGCTTGGCCAATCTATTGGGCCATCCAAGGCTGCATTCTCACCGGTGTTTGGGTGATTGCTCATGAGTGTGGCCACCATGCCTTCAGCAAGTACCAACTTGTTGATGACATGGTTGGTTTGACCCTTCACTCTTGTCTATTAGTTCCTTATTTCTCATGGAAAATCAGCCACCGCCGCCACCACTCCAACACAGGTTCCCTCAGACCGCGACGAAGTGTTTGTCCCGAAACCAAAATCAAAGGTATCATGGTATAACAAGTACATGAACAATCCACCAGGGAGGGCTATTTCCCTTTTCATCACACTCACACTAGGATGGCCAAT

>37_FAD2_Plate_Plate 01_D01

TCCNAACCCTCCATTCAGTGTTGGCCAACTCAAGAAAGCAATTCCACCACATTGCTTTGAACGTTCTCTTCTCATATCATTCTCATATGTTGTCTATGATCTCTTAATGGCCTACTTACTCTTCTACATTGCCACCACTTATTTCCACAAGCTTCCATACCCATTTTCCTTCCTTGCTTGGCCAATCTATTGGGCCATCCAAGGCTGCATTCTCACCGGTGTTTGGGTGATTGCTCATGAGTGTGGCCACCATGCCTTCAGCAAGTGCCAACTTGTTGATGACATGGTTGGTTTGACCCTTCACTCTTGTCTATTAGTTCCTTATTTCTCATGGAAAATCAGCCACCGCCGCCACCACTCCAACACAGGTTCCCTCAGACCGCGACGAAGTGTTTGTCCCGAAACCAAAATCAAAGGTATCATGGTATAACAAGTACATGAACAATCCACCAGGGAGGGCTATTTCCCTTTTCATCACACTCACACTAGGATGGCCAAT

>38_FAD2_Plate_Plate 01_D02

TTCAAACCCTCCATTCAGTGTTGGCCAACTCAAGAAAGCAATTCCACCACATTGCTTTGAACGTTCTCTTTTCATATCATTCTCCTATGTTGTCTATGATCTCTTAATGGCCTACTTACTCTTCTACATTGCCACCACTTATTTCCACCAGCTTCCATACCCATTTTCCTTCCTTGCTTGGCCAATCTATTGGGCCATCCAAGGCTGCATTCTCACTGGTGTTTGGGTGATTGCTCATGAGTGTGGCCACCATGCCTTCAGCAAGTATCAACTTGTTGATGACATGGTTGGTTTGATCCTTCACTCTTGTCTATTAGTCCCTTATTTCTCATGGAAAATCAGCCACCGCCACCTCCACTCCAACACCGGTTCCCTCGACCGCGACTAAATGTTTGTCCTGNAACCAAAAACAAAGGNAN

>39_FAD2_Plate_Plate 01_D03

TTNAAACCCTCCATTCAGTGTTGGCCAACTCAAGAAAGCAATTCCACCACATTGCTTTGAACGTTCTCTTTTCATATCATTCTCATATGTTGTCTATGATCTCTTAATGGCCTACTTACTCTTCTACATTGCCACCACCTATTTCCACAAGCTTCCATACCCATTTTCCTTCCTTGCTTGGCCAATCTATTGGGCCATCCAAGGCTGCATTCTCACCGGTGTTTGGGTGATTGCTCATGAGTGTGGCCACCATGCCTTCAGCAAGTACCAACTTGTTGATGACATGGTTGGTTTGACCCTTCACTCTTGTCTATTAGTTCCTTATTTCTCATGGAAAATCAGCCACCGCCGCCACCACTCCAACACAGGTTCCCTCAGACCGCGACGAAGTGTTTGTCCCGAAACCAAAATCAAAGGTATCATGGTATAACAAGTACATGAACAATCCACCAGGGAGGGCTATTTCCCTTTTCATCACACTCACACTAGGATGGCCAAT

>40_FAD2_Plate_Plate 01_D04

TTNNAACCCTCCATTCAGTGTTGGCCAACTCAAGAAAGCAATTCCACCACATTGCTTTGAACGTTCTCTTTTCATATCATTCTCCTATGTTGTCTATGATCTCTTGGTGGCCTACTTACTCTTCTACATTGCCACCACTTATTTCCACAAGCTTCCATACCCATTTTCCTTCCTTGCTTGGCCAATCTATTGGGCCATCCAAGGCTGCATTCTCACTGGTGTTTGGGTGATTGCTCATGAGTGTGGCCACCATGCCTTCAGCAAGTACCAACTTGTTGATGACATGGTTGGTCTGACCCTTCACTCTTGTCTATTAGTTCCTTATTTCTCATGGAAAATCAGCCACCGCCGCCACCACTCCAACACCGGTTCCCTCGACCGCAACGAAGTGTTTGTCCCAAAACCAAGATCAAAGGTATCATGGTATAACAAGTACATGAACAATCCACCAGGGAGGGCTATCTCCCTCTTCATCACACTCACACTAGGATGGCCAAT

>41_FAD2_Plate_Plate 01_D05

TTCNAACCCTCCATTCAGTGTTGGCCAACTCAAGAAAGCAATTCCACCACATTGCTTTGAACGTTCTCTTTTCATATCATTCTCATATGTTGTCTATGATCTCTTAATGGCCTACTTACTCTTCTACATTGCCACCACTTATTTCCACAAGCTTCCATACCCATTTTCCTTCCTTGCTTGGCCAATCTATTGGGCCATCCAAGGCTGCATTCTCACCGGTGTTTGGGTGATTGCTCATGAGTGTGGCCACCATGCCTTCAGCAAGTACCAACTTGTTGATGACATGGTTGGTTTGACCCTTCACTCTTGTCTATTAGTTCCTTATTTCTCATGGAAAATCAGCCACCGCCGCCACCACTCCAACACAGGTTCCCTCAGACCGCGACGAAGTGTTTGTCCCGAAACCAAAATCAAAGGTATCATGGTATAACAAGTACATGAACAATCCACCAGGGAGGGCTATTTCCCTTTTCATCACACTCACACTAGGATGGCCAAT

>42_FAD2_Plate_Plate 01_D06

TTNAAACCCTCCATTCAGTGTTGGCCAACTCAAGAAAGCAATTCCACCACATTGCTTTGAACGTTCTCTTTTCATATCATTCTCCTATGTTGTCTATGATCTCTTAATGGCCTACTTACTCTTCTACATTGCCACCACTTATTTCCACCAGCTTCCATACCCATTTTCCTTCCTTGCTTGGCCAATCTATTGGGCCATCCAAGGCTGCATTCTCACTGGTGCTTGGGTGATTGCTCATGAGTGTGGCCACCATGCCTTCAGCAAGTATCAACTTGTTGATGACATGGTTGGTTTGATCCTTCACTCTTGTCTATTAGTCCCTTATTTCTCATGGAAAATCAGCCACCGCCACCTCCACTCCAACACCGGTTCCCTCGACCGCGACTAAGTGTTTGTCCCGAAACCAAAATCAAAGGTATCACGGTATAACAAGNACATGAACAATCCACTAAAGAGGGCTATTTCCCTTTTCATCANACTCCCCTANAATGNNCANT

>43_FAD2_Plate_Plate 01_D07

TTNNAACCCTCCATTCAGTGTTGGCCAACTCAAGAAAGCAATTCCACCACATTGCTTTGAACGTTCTCTTTTCATATCATTCTCATATGTTGTCTATGATCTCTTAGTGGCCTACTTACTCTTCTACATTGCCACCACTTATTTCCACAAGCTTCCATACCCATTTTCCTTCCTTGCTTGGCCAATCTATTGGGCCATCCAAGGCTGCATTCTCACCGGTGTTTGGGTGATTGCTCATGAGTGTGGCCACCATGCCTTCAGCAAGTACCAACTTGTTGATGACATGGTTGGTTTGACCCTTCACTCTTGTCTATTAGTTCCTTATTTCTCATGGAAAATCAGCCACCGCCGCCACCACTCCAACACAGGTTCCCTCGACCGCGACGAAGTGTTTGTCCCGAAACCAAAATCAAAGGTATCATGGTATAACAAGTACATGAACAATCCACCAGGGAGGGCTATTTCCCTTTTCATCACACTCACACTAGGATGGCCAAT

>44_FAD2_Plate_Plate 01_D08

TTCNAACCCTCCATTCAGTGTTGGCCAACTCAAGAAAGCAATTCCACCACATTGCTTTGAACGTTCTCTTTTCATATCATTCTCCTATGTTGTCTATGATCTCTTAGTGGCCTACTTACTCTTCTACATTGCCACCACTTATTTCCACAAGCTTCCATACCCATTTTCCTTCCTTGCTTGGCCAATCTATTGGGCCATCCAAGGCTGCATTCTCACTGGTGTTTGGGTGATTGCTCATGAGTGTGGCCACCATGCCTTCAGCAAGTACCAACTTGTTGATGACATGGTTGGTTTGACCCTTCACTCTTGTCTATTAGTTCCTTATTTCTCATGGAAAATCAGCCACCGCCGCCACCACTCCAACACCGGTTCCCTCGACCGCAACGAAGTGTTTGTCCCAAGACCAAAATCAAAGGTATCATGGTATAACAAGTACATGAACAATCCACCAGGGAGGGCTATCTCCCTCTTCATCACACTCACACTAGGATGGCCAAT

>45_FAD2_Plate_Plate 01_D09

NTTCNNCCCTCCATTCAGTGTTGGCCAACTCAAGAAAGCAATTCCACCCCNTTGCTTTGAACGTTCTCTTTTCATATCATTCTCCTATGTTGTCTATGATCTCTTAATGGCCTACTTACTCTTCTACATTGCCACCATTTATTTCCACCAGCTTCCATACCCATTTTCCTTCCTTGCTTGGCCAATCTATTGGGCCATCCAAGGCTGCATTCTCACTGGTGTTTGGGTGATTGCTCATGAGTGTGGCCACCATGCCTTCAGCAAGTATCAACTTGTTGATGACATGGTTGGTTTGATCCTTCACTCTTGTCTATTAGTCCCTTATTTCTCATGGAAAATCAGCCACCGCCACCTCCACTCCAACACCGGTTCCCTCGACCGCGACTAAGTGTTTGTCCTGAAACCAAAATCAAAGGTATCACGGTATAACAAGTACATGAACAATCCACCAGGGAGGGCTATCTCCCTCTTCATCACACTCACACTAGGATGGCCAAT

>47_FAD2_Plate_Plate 01_D11

TTCAAACCCTCCATTCAGTGTTGGCCAACTCAAGAAAGCAATTCCACCACNTTGCTTTGAACGTTCTCTTTTCATATCATTCTCCTATGTTGTCTATGATCTCTTAATGGCCTACTTACTCTTCTACATTGCCACCACTTGTTTCCACCATCTTCCATACCCATTTTCCTTCCTTGCTTGGCCAATCTATTGGGCCATCCAAGGCTGCATTCTCACTGGTGTTTGGGTGATTGCTCATGAGTGTGGCCACCATGCCTTCAGCAAGTATCAACTTGTTGATGACATGGTTGGTTTGATCCTTCACTCTTGTCTATTAGTCCCTTATTTCTCATGGAAAATCAGCCACCGCCACCTCCACTCCAACACCGGTTCCCTCGACCGCGACTAAGTGTTTGTCCTGAAACCAAAATCAAAGGTATCACGGTATAACAAGTACATGAACAATCCACTANAAAGGGCTATTTCCCTTTTCATCACNCTCACACTANGATGGNCAAT

>52_FAD2_Plate_Plate 01_E04

NNNNNNCCCNCCNTNCAGTGTTGGCCAACTCAAGAAACCAATTCCACCACNTTGCTTTGAACGTTCTCTTTCCNTATCATTCTCATATGTTGTCTATGATCTCTTAATGGCCTACTTACTTTTCTACATTGCCACCACTTATTTCCACAAGCTTCCATACCCATTTTCCTTCCTTGCTTGGCCAATCTATTGGGCCATCCAAGGCTGCATTCTCACCGGTGTTTGGGTGATTGCTCATGAGTGTGGCCACCATGCCTTCAGCAAGTACCAACTTGTTGATGACATGGTTGGTTTGACCCTTCACTCTTGTCTATTAGTTCCTTATTTCTCATGGAAAATCAGCCACCGCCGCCACCACTCCAACACAGGTTCCCCCGACCGCGACGAAGTGTTTGTCCCGAAACCAAAATCAAAGGTATCATGGTATAACAAGTACATGAACAATCCACCAGGGAGGGCTATTTCCCTTTTCATCACACTCACACTANGATGGCCAT

>53_FAD2_Plate_Plate 01_E05

TTCAAACCCTCCATTCAGTGTTGGCCAACTCAAGAAAGCAATTCCACCACATTGCTTTGAACGTTCTCTTTTCATATCATTCTCCTATGCTGTCTATGATCTCTTAATGGCCTACTTACTCTTCTACATTGCCACCACTTATTTCCACCAGCTTCCATACCCATTTTCCTTCCTTGCTTGGCCAATCTATTGGGCCATCCAAGGCTGCATTCTCACTGGTGTTTGGGTGATTGCTCATGAGTGTGGCCACCATGCCTTCAGCAAGTATCAACTTGTTGATGACATGGTTGGGTTGATCCTTCACTCTTGTCTATTAGTCCCTTATTTCTCATGGAAAATCAGCCACCGCCACCTCCACTCCAACACCGGTTCCCTCGACCGCGACTAAGTGTTTGTCCTGAAACCAAAATCAAAGGTATCACGGTATAACAAGTACATGAACAATCCACTAAAGAGGGCTATTTCCCTTTTCATCANANTCNCACTANGATGGCCAAT

>54_FAD2_Plate_Plate 01_E06

TTCNAACCCTCCATTCAGTGTTGGCCAACTCAAGAAAGCAATTCCACCACATTGCTTTGAACGTTCTCTTTTCATATCATTCTCCTATGTTGTCTATGATCTCTTAATGGCCTACTTACTCTTCTACATTGCCACCACTTATTTCCACCAGCTTCCATACCCATTTTCCTTCCTTGCTTGGCCAATCTATTGGGCCATCCAAGGCTGCATTCTCACTGGTGTTTGGGTGATTGCTCATGAGTGTGGCCACCATGCCTTCAGCAAGTATCAACTTGTTGATGACATGGTTGGTTTGATCCTTCACTCTTGTCTATCAGTCCCTTATTTCTCATGGAAAATCAGCCACCGCCACCTCCACTCCAACACCGGTTCCCTCGACCGCGACTAAGTGTTTGTCCTGAAACCAAAATCAAAGGTATCACGGTATAACAGGTACATGAACAATCCACTAGAGAGGGCTATTTCCCTTTTCATCACACTCACACTAGGATGGCCAAT

>55_FAD2_Plate_Plate 01_E07

TTCNAACCCTCCATTCAGTGTTGGCCAACTCAAGAAAGCAATTCCACCACATTGCTTTGAACGTTCTCTTTTCATATCATTCTCCTATGTTGTCTATGATCTCTTAGTGGCCTACTTACTCTTCTACATTGCCACCACTTATTTCCACAAGCTTCCATACCCATTTTCCTTCCTTGCTTGGCCAATCTATTGGGCCATCCAAGGCTGCATTCTCACTGGTGTTTGGGTGATTGCTCATGAGTGTGGCCACCATGCCTTCAGCAAGTACCAACTTGTTGATGACATGGTTGGTTTGACCCTTCACTCTTGTCTATTAGTTCCTTATTTCTCATGGAAAATCAGCCACCGCCGCCACCACTCCAACACCGGTTCCCTCGACCGCAACGAAGTGTTTGTCCCAAAACCAAAATCAAAGGTATCATGGTATAACAAGTACATGAACAATCCACCAGGGAGGGCTATCTCCCTCTTCATCACACTCACACTANGATGGCCAAT

>56_FAD2_Plate_Plate 01_E08

TTCAAACCCTCCATTCANTGTTGGCCAACTCAAGAAAGCAATTCCNCCACATTGCTTTGAACGTTCTCTTTTCATATCATTCTCATATGTTGTCTATGATCTCTTAATGGCCTACTTACTCTTCTACATTGCCACCACTTATTTCCACAAGCTTCCATACCCATTTTCCTTCCTTGCTTGGCCAATCTATTGGGCCATCCAAGGCTGCATTCTCACCGGTGTTTGGGTGATTGCTCATGAGTGTGGCCACCATGCCTTCAGCAAGTACCAACTTGTTGATGACATGGTTGGTTTGACCCTTCACTCTTGTCTATTAGTTCCTTATTTCTCATGGAAAATCAGCCACCGCCGCCACCACTCCAACACAGGTTCCCTCGACCGCGACGAAGTGTTTGTCCCGAAACCAAAATCAAAGGTATCATGGTATAACAAGTACATGAACAATCCACCAGGGAGGGCTATTTCCCTTTTCATCACACTCACACTAGGATGGCCAAT

>57_FAD2_Plate_Plate 01_E09

TTCNAACCCTCCATTCAGTGTTGGCCAACTCAAGAAAGCAATTCCACCACATTGCCTTGAACGTTCTCTTTTCATATCATTCTCCTATGTTGTCTATGATCTCTTAGTGGCCTACTTACTCTTCTACATTGCCACCACTTATTTCCACAAGCTTCCATACCCATTTTCCTTCCTTGCTTGGCCAATCTATTGGGCCATCCAAGGCTGCATTCCCACTGGTGTTTGGGTGATTGCTCATGAGTGTGGCCACCATGCCTTCAGCAAGTACCAACTTGTTGATGACATGGTTGGTTTGACCCTTCACTCTTGTCTATTAGTTCCTTATTTCTCATGGAAAATCAGCCACCGCCGCCACCACTCCAACACCGGTTCCCTCGACCGCAACGAAGTGTTTGTCCCAAAACCAAAATCAAAGGTATCATGGNATAACAAGTACATGAACNATCCACCANGGAGGGCTATCTCCCTCTTCATCACACTCACACTAGGATGGCCAAC

>1_FAD2_Plate_Plate 01_A01

TTCAANCCCTCCATTCAGTGTTGGCCAACTCAAGAAAGCAATTCCACCACATTGCTTTGAACGTTCTCTTTTCATATCATTCTCATATGTTGTCTATGATCTCTTAATGGCCTACTTACTCTTCTACATTGCCACCACTTATTTCCACAAGCTTCCATACCCATTTTCCTTCCTTGCTTGGCCAATCTATTGGGCCATCCAAGGCTGCATTCTCACCGGTGTTTGGGTGATTGCTCATGAGTGTGGCCACCATGCCTTCAGCAAGTACCAACTTGTTGATGACATGGTTGGTTTGACCCTTCACTCTTGTCTATTAGTTCCTTATTTCTCATGGAAAATCAGCCACCGCCGCCACCACTCCAACACAGGTTCCCTCGACCGCGGCGAAGTGTTTGTCCCGAAACCAAAATCAAAGGTATCATGGTATAACAAGTACATGAACAATCCACCAGGGAGGGCTATTTCCCTTTTCATCACACTCACACTAGGATGGCCAAT

>2_FAD2_Plate_Plate 01_A02

TTCNNACCCTCCATTCAGTGTTGGCCAACTCAAGAAAGCAATTCCACCACATTGCTTTGAACGTTCTCTTTTCATATCATTCTCCTATGTTGTCTATGATCTCTTAGTGGCCTACTTACTCTTCTACATTGCCACCACTTATTTCCACAAGCTTCCATACCCATTTTCCTTCCTTGCTTGGCCAATCTATTGGGCCATCCAAGGCTGCATTCTCACTGGTGTTTGGGTGATTGCTCATGAGTGTGGCCACCATGCCTTCAGCAAGTACCAACTTGTTGATGACATGGTTGGTTTGACCCTTCACTCTTGTCTATTAGTTCCTTATTTCTCATGGAAAATCAGCCACCGCCGCCACCACTCCAACACCGGTTCCCTCGACCGCAACGAAGTGTTTGTCCCAAAACCAAAATCAAAGGTATCATGGTATAACAAGTACATGAACAATCCACCAGGGAGGGCTATCTCCCTCTTCATCACACTCACACTAGGATGGCCAAT

>3_FAD2_Plate_Plate 01_A03

TTCAANCCCTCCATTCAGTGTTGGCCAACTCAAGAAAGCAATTCCACCACATTGCTTTGAACGTTCTCTTTTCATATCATTCTCATATGTTGTCTATGATCTCTTAATGGCCTACTTACTCTTCTACATTGCCACCACTTATTTCCACAAGCTTCCATACCCATTTTCCTTCCTTGCTTGGCCAATCTATTGGGCCATCCAAGGCTGCATTCTCACCGGTGTTTGGGTGATTGCTCATGAGTGTGGCCACCATGCCTTCAGCAAGTACCAACTTGTTGATGACATGGTTGGTTTGACCCTTCACTCTTGTCTATTAGTTCCTTATTTCTCATGGAAAATCAGCCACCGCCGCCACCACTCCAACACAGGTTCCCTCGACCGCGACGAAGTGTTTGTCCCGAAACCAAAATCAAAGGTATCATGGTATAACAAGTACATGAACAATCCACCAGGGAGGGCTATTTCCCTTTTCATCACACTCACACTAGGATGGCCAAT

>4_FAD2_Plate_Plate 01_A04

TTCAAACCCTCCATTCAGTGTTGGCCAACTCAAGAAAGCAATTCCACCACATTGCTTTGAACGTTCTCTTTTCATATCATTCTCCTATGTTGTCTATGATCTCTTAATGGCCTACTTACTCTTCTACATTGCCACCACTTATTTCCACCAGCTTCCATACCCATTTTCCTTCCTTGCTTGGCCAATCTATTGGGCCATCCAAGGCTGCATTCTCACTGGTGTTTGGGTGATTGCTCATGAGTGTGGCCACCATGCCTTCAGCAAGTATCAACTTGTTGATGACATGGTTGGTTTGATCCTTCACTCTTGTCTATTANTCCCTTATTTCTCATGGAAAATCAGCCACCGCCACCTCCACTCCAACACCGGTTCCCTCGACCGCGACTAAGTGNTTGTCCTGAAACCAAAATCNAAGGTAT

>5_FAD2_Plate_Plate 01_A05

TTCAANCCCTCCATTCAGTGTTGGCCAACTCAAGAAAGCAATTCCACCACATTGCTTTGAACGTTCTCTTTTCATATCATTCTCATATGTTGTCTATGATCTCTTAATGGCCTACTTACTCTTCTACATTGCCACCACTTATTTCCACAAGCTTCCATACCCATTTTCCTTCCTTGCTTGGCCAATCTATTGGGCCATCCAAGGCTGCATTCTCACCGGTGTTTGGGTGATTGCACATGAGTGTGGCCACCATGCCTTCAGCAAGTACCAACTTGTTGATGACATGGTTGGTTTGACCCTTCACTCTTGTCTATTAGTTCCTTATTTCTCATGGAAAATCAGCCACCGCCGCCACCACTCCAACACAGGTTCCCTCGACCGCGACGAAGTGTTTGTCCCGAAACCAAAATCAAAGGTATCATGGTATAACAAGTACATGAACAATCCACCAGGAAGGGCTATTTCCCTTTTCATCACACTCACACTAGGATGGCCAAT

>6_FAD2_Plate_Plate 01_A06

TTNNAACCCTCCATTCAGTGTTGGCCATCTCAAGAAAGCAATTCCACCACATTGCTTTGAACGTTCTCTTTTCATATCATTCTCCTATGTTGTCTATGATCTCTTAGTGGCCTACTTACTCTTCTACATTGCCACCACTTATTTCCACAAGCTTCCACACCCATTTTCCTTCCTTGCTTGGCCAATCTATTGAGCCATCCAAGGCTGCATTCTCACTGGTGTTTGGGTGATTGCTCATGAGTGTGGCCACCATGCCTTCAGCAAGTACCAACTTGTTGATGACATGGTTGGTTTGACCCTTCACTCTTGTCTATTAGTTCCTTATTTCTCATGGAAAATCAGCCACCGCCGCCACCACTCCAACACCGGTTCCCTCGACCGCAACGAAGTGTTTGTCCCAAAACCAAAATCAAAGGTATCATGGTATAACAAGTACATGAACAATCCACCAGGGAGGGCTATCTCCCTCTTCATCACACTCACACTAGGATGGCCAAT

>7_FAD2_Plate_Plate 01_A07

TTCAAACCCTCCATTCAGTGTTGGCCAACTCAAGAAAGCAATTCCACCACATTGCTTTGAACGTTCTCTTTTCATATCATTCTCATATGTTGTCTATGATCTCTTAATGGCCTACTTACTCTTCTACATTGCCACCACTTATTTCCACAAGCTTCCATACCCATTTTCCTTCCTTGCTTGGCCAATCTATTGGGCCACCCAAGGCTGCATTCTCACCGGTGTTTGGGTGATTGCTCATGAGTGTGGCCACCATGCCTTCAGCAAGTACCAACTTGTTGATGACATGGTTGGTTTGACCCTTCACTCTTGTCTATTAGTTCCTTATTTCTCATGGAAAATCAGCCACCGCCGCCACCACTCCAACACAGGTTCCCTCGACCGCGACGAAGTGTTTGTCCCGAAACCAAAATCAAAGGTATCATGGTATAACAAGTACATGAACAATCCACCAGGGAGGGCTATTTCCCTTTTCATCACACTCACACTAGGATGGCCAAT

>8_FAD2_Plate_Plate 01_A08

TTCAAACCCTCCATTCAGTGTTGGCCAACTCAAGAAAGCAATTCCACCACATTGCTTTGAACGTTCTCTTTCCATATCATTCTCCTATGTTGTCTATGATCTCTTAGTGGCCTACTTACTCTTCTACATTGCCACCACTTATTTCCACAAGCTTCCATACCCATTTTCCTTCCTTGCTTGGCCAATCTATTGGGCCATCCAAGGCTGCATTCTCACTGGTGTTTGGGTGATTGCTCATGAGTGTGGCCACCATGCCTTCAGCAAGTACCAACTTGTTGATGACATGGTTGGTTTGACCCTTCACTCTTGTCTATTAGTTCCTTATTTCTCATGGAAAATCAGCCACCGCCGCCACCACTCCAACACCGGTTCCCTCGACCGCAACGAAGTGTTTGTCCCAAAACCGAAATCAAAGGTATCATGGTATAACAAGTACATGAACAATCCACCAGGGAGGGCTATCTCCCTCTTCATCACACTCACACTAGGATGGCCAAT

>9_FAD2_Plate_Plate 01_A09

TTCNAACCCTCCATTCAGTGTTGGCCAACTCAAGAAAGCAATTCCACCACATTGCTTTGAACGTTCTCTTTTCATATCATTCTCCTATGTTGTCTATGATCTCTTAATGGCCTACTTACTCTTCTACATTGCCACCACTTATTTCCACCAGCTTCCATACCCATTTTCCTTCCTTGCTTGGCCAATCTATTGGGCCATCCAAGGCTGCATTCTCACTGGTGTTTGGGTGATTGCTCATGAGTGTGGCCACCATGCCTTCAGCAAGTATCAACTTGTTGATGACATGGTTGGTTTGATCCTTCACTCTTGTCTATTAGTCCCTTATTTCTCATGGAAAATCAGCCACCGCCACCTCCACTCCAACACCGGTTCCCTCGACCGCGACTAAGTGTTTGTCCTGAAACCAAAATCAAAGGTATCACGGTATAACAAGTACATGAACAATCCACTAGAGAGGGCTATTTCCCTTTTCATCACACTCACACTAGGATGGCCAAT

>10_FAD2_Plate_Plate 01_A10

TTCNAACCCTCCATTCAGTGTTGGCCAACTCAAGAAAGCAATTCCACCACATTGCTTTGAACGTTCTCTTTTCATATCATTCTCCTATGTTGTCTATGATCTCTTAGTGGCCTACTTACTCTTCTACATTGCCACCACTTATTTCCACAAGCTTCCATACCCATTTTCCTTCCTTGCTTGGCCAATCTATTGGGCCATCCAAGGCTGCATCTCACTGGTGTTTGGGTGATTGCTCATGAGTGTGGCCACCATGCCTTCAGCAAGTACCAACTTGTTGATGACATGGTTGGTTTGACCCTTCACTCTTGTCTATTAGTTCCTTACTTCTCATGGAAAATCAGCCACCGCCGCCACCACTCCAACACCGGTTCCCTCGACCGCAACGAAGTGTTTGTCCCAAAACCAAAATCAAAGGTATCATGGTATAACAAGTACATGAACAATCCACCAGGGAGGGCTATCTCCCTCTTCATCACACTCACACTAGGATGGCCAAT

>13_FAD2_Plate_Plate 01_B01

TTCNAACCCTCCATTCAGTGTTGGCCAACTCAAGAAAGCAATTCCACCACATTGCTTTGAACGTTCTCTTTTCATATCATTCTCATATGTTGTCTATGATCTCTTAATGGCCTACTTACTCTTCTACATTGCCACCACTTATTTCCACAAGCTTCCATACCCATTTTCCTTCCTTGCTTGGCCAATCTATTGGGCCATCCAAGGCTGCATTCTCACCGGTGTTTGGGTGATTGCTCATGAGTGTGGCCACCATGCCTTCAGCAAGTACCAACTTGTTGATGACATGGTTGGTTTGACCCTTCACTCTTGTCTATTAGTTCCTTATTTCTCATGGAAAATCAGCCACCGCCGCCACCACTCCAACACAGGTTCCCTCAGACCGCGACGAAGTGTTTGTCCCGAAACCAAAATCAAAGGTATCATGGTATAACAAGTACATGAACAATCCACCAGGGAGGGCTACTTNCCTTTTCATCACACTCACACTAGGATGGCCAAT

>15_FAD2_Plate_Plate 01_B03

TTCAAACCCTCCATTCAGTGTTGGCCAACTCAAGAAAGCAATTCCACCACATTGCTTTGAACGTTCTCTTTTCACATCATTCTCATATGTTGTCTATGATCTCTTAATGGCCTACTTACTCTTCTACATTGCCACCACTTATTTCCACAAGCTTCCATACCCATTTTCCTTCCTTGCTTGGCCAATCTATTGGGCCATCCAAGGCTGCATTCTCACCGGTGTTTGGGTGATTGCTCATGAGTGTGGCCACCATGCCTTCAGCAAGTACCAACTTGTTGATGACATGGTTGGTTTGACCCTTCACTCTTGTCTATTAGTTCCTTATTTCTCATGGAAAATCAGCCACCGCCGCCACCACTCCAACACAGGTTCCCTCAGACCGCGACGAAGTGTTTGTCCCGAAACCAAAATCAAAGGTATCATGGTATAACAAGTACATGAACAATCCACCAGGGGGGGCTATTTCCCTTTTCATCACACTCACACTAGGATGGCCAAT

>16_FAD2_Plate_Plate 01_B04

NTNNANCCCTCCATTCAGTGTTGGCCAACTCAAGAAAGCAATTCCACCACATTGCTTTGAACGTTCTCTTTTCATATCATTCTCCTATGTTGTCTATGATCTCTTAATGGCCTACTTACTCTTCTACATTGCCACCACTTATTTCCACCAGCTTCCATACCCATTTTCCTTCCTTGCTTGGCCAATCTATTGGGCCATCCAAGGCTGCATTCTCACTGGTGTTTGGGTGATTGCTCATGAGTGTGGCCACCATGCCTTCAGCAAGTATCAACTTGTTGATGACATGGTTGGTTTGATCCTTCACTCTTGTCTATTAGTCCCTTATTTCTCATGGAAAATCAGCCACCGCCACCTCCACTCCAACACCGGTTCCCTCGACCGCGACTAAGTGTTTGTCCTGAAACCAAAATCAAAGGTATCACGGTATAACAAGTACATGAACAATCCACTAGAGAGGGCTATTGCCCTTTTCATCACACTCACACTAGGATGGCCAAT

>17_FAD2_Plate_Plate 01_B05

TTCNAACCCTCCATTCAGTGTTGGCCAACTCAAGAAAGCAATTCCACCACATTGCTTTGAACGTTCTCTTTTCATATCATTCTCCTATGTTGTCTATGATCTCTTAATGGCCTACTTACTCTTCTACATTGCCACCACTTATTTCCACCAGCTTCCATACCCATTTTCCTTCCTTGCTTGGCCAATCTATTGGGCCATCCAAGGCTGCATTCTCACTGGTGTTTGGGTGATTGCTCATGAGTGTGGCCACCATGCCTTCAGCAAGTATCAACTTGTTGATGACATGGTTGGTTTGATCCTTCACTCTTGTCTATTAGTCCCTTATTTCTCATGGAAAATCAGCCACCGCCACCTCCACTCCAACACCGGTTCCCTCGACCGCGACTAAGTGTTTGTCCTGAAACCAAAATCAAAGGTATCACGGTATAACAAGTACATGAACAATCCACTAGAGAGGGCTATTTCCCTTTTCATCACACTCACACTAGGATGGCCAAT

>19_FAD2_Plate_Plate 01_B07

TTCAAACCCTCCATTCAGTGTTGGCCAACTCAAGAAAGCAATTCCACCACATTGCTTTGAACGTTCTCTTTTCATATCATTCTCATATGTTGTCTATGATCTCTTAATGGCCTACTTACTCTTCTACATTGCCACCACTTATTTCCACAAGCTTCCATACCCATTTTCCTTCCTTGCTTGGCCAATCTATTGGGCCATCCAAGGCTGCATTCTCACCGGTGTTTGGGTGATTGCTCATGAGTGTGGCCACCATGCCTTCAGCAAGTACCAACTTGTTGATGACATGGTTGGTTTGACCCTTCACTCTTGTCTATTAGTTCCTTATTTCTCATGGAAAATCAGCCACTGCCGCCACCACTCCAACACAGGTTCCCTCAGACCGCGACGAAGTGTTTGTCCCGAAACCAAAATCAAAGGTATCATAGTATAACAAGTACATGAACAATCCACCAGGGAGGGCTATTCCCCTTTTCATCACACTCACACTANGATGGGCCAA

>21_FAD2_Plate_Plate 01_B09

TTCNAACCCTCCATTCAGTGTTGGCCAACTCAAGAAAGCAATTCCACCACATTGCTTTGAACGTTCTCTTTTCATATCATTCTCCTATGTTGTCTATGATCTCTTAATGGCCTACTTACTCTTCTACATTGCCACCACTTATTTCCACCAGCTTCCAAACCCATTTTCCTTCCTTGCTTGGCCAATCTATTGGGCCATCCAAGGCTGCATTCTCACTGGTGTTTGGGTGATTGCTCATGAGTGTGGCCACCATGCCTTCAGCAAGTATCAACTTGTTGATGACATGGTTGGTTTGATCCTTCACTCTTGTCTATTAGTCCCTTATTTCTCATGGAAAATCAGCCACCGCCACCTCCACTCCGACACCGGTTCCCTCGACCGCGACTAAGTGTTTGTCCTGAAACCAAAATCAAAGGTATCACGGTATAACAAGTACATGGACAATCCACTAGAGAGGGCTATTTCCCTTTTCATCACACTCACACTAGGATGGCCAAT

>22_FAD2_Plate_Plate 01_B10

NTTCAACCCTCCATTCAGTGTTGGCCAACTCAAGAAAGCAATTCCACCACATTGCTTTGAACGTTCTCTTTTCATATCATTCTCCTATGTTGTCTATGATCTCTTAGTGGCCTACTTACTCTTCTACATTGCCACCACTTATTTCCACAAGCTTCCATACCCATTTTCCTTCCTTGCTTGGCCAATCTATTGGGCCATCCAAGGCTGCATTCTCACTGGTGTTTGGGTGATTGCTCATGAGTGTGGCCACCATGCCTTCAGCAAGTACCAACTTGTTGATGACATGGTTGGTTTGACCCTTCACTCTTGTCTATTAGTTCCTTATTTCTCATGGAAAATCAGCCACCGCCGCCACCACTCCAACACCGGTTCCCTCGACCGCAACGAAGTGTTTGTCCCAAAACCAAAATCAAAGGTATCATGGTATAACAAGTACATGAACAATCCACCAGGGAGGGCTATCTCCCTCTTCATCACACTCACACTAGGATGGCCAAT

>23_FAD2_Plate_Plate 01_B11

TTCNAACCCTCCATTCAGTGTTGGCCAACTCAAGAAAGCAATTCCACCACATTGCTTTGAACGTTCTCTTTTCATATCATTCTCATATGTTGTCTATGATCTCTTAATGGCCTACTTACTCTTCTACATTGCCACCACTTATTTCCACAAGCTTCCATACCCATTTTCCTTCCTTGCTTGGCCAATCTATTGGGCCATCCAAGGCTGCATTCTCACCGGTGTTTGGGTGATTGCTCATGAGTGTGGCCACCATGCCTTCAGCAAGTACCAACTTGTTGATGACATGGTTGGTTTGACCCTTCGCTCTTGTCTATTAGTTCCTTATTTCTCATGGAAAATCAGCCACCGCCGCCACCACTCCAACACAGGTTCCCTCGACCGCGACGAAGTGTTTGTCCCGAAACCAAAATCAAAGGTATCATGGTATAACAAGTACATGAACAATCCACCAGGGAGGGCTATTTCCCTTTTCATCACACTCACACTAGGATGGCCAAT

>24_FAD2_Plate_Plate 01_B12

TTCNAACCCTCCATTCAGTGTTGGCCAACTCAAGAAAGCAATTCCACCACATTGCTTTGAACGTTCTCTTTTCATATCATTCTCATATGTTGTCTATGATCTCTTAATGGCCTACTTACTCTTCTACATTGCCACCACTTATTTCCACAAGCTTCCATACCCATTTTCCTTCCTTGCTTGGCCAATCTATTGGGCCATCCAAGGCTGCATTCTCACCGGTGTTTGGGTGATTGCTCATGAGTGTGGCCACCATGCCTTCAGCAAGTACCAACTTGTTGATGACATGGTTGGTTTGACCCTTCACTCTTGTCTATTAGTTCCTTATTTCTCATGGAAAATCAGCCACCGCCGCCACCACTCCAACACAGGTTCCCTCGACCGCGACGAAGTGTTTGTCCCGAAACCAAAATCAAAGGTATCATGGTATAACAAGTACATGAACAATCCACCAGGGAGGGCTATTTCCCTTTTCATCACACTCACACTAGGATGGCCAAT

>25_FAD2_Plate_Plate 01_C01

TTCNAACCCTCCATTCAGTGTTGGCCAACTCAAGAAAGCAATTCCACCACATTGCTTTGAACGTTCTCTTTTCATATCATTCTCCTATGTTGTCTATGATCTCTTAATGGCCTACTTACTCTTCTACATTGCCACCACTTATTTCCACCAGCTTCCATACCCATTTTCCTTCCTTGCTTGGCCAATCTATTGGGCCATCCAAGGCTGCATTCTCACTGGTGTTTGGGTGATTGCTCATGAGTGTGGCCACCATGCCTTCAGCAAGTATCAACTTGTTGATGACATGGTTGGTTTGATCCTTCACTCTTGTCTATTAGTCCCTTATTTCTCATGGAAAATCAGCCACCGCCACCTCCACTCCAACACCGGTTCCCTCGACCGCGACTAAGTGTTTGTCCTGAAACCAAAATCAAAGGTATCACGGTATAACAAGTACATGAACAATCCACTAGAGAGGGCTATTTCCCTTTTCATCACACTCACACTAGGATGGCCAAT

>27_FAD2_Plate_Plate 01_C03

TTCNAACCCTCCATTCAGTGTTGGCCAACTCAAGAAAGCAATTCCACCACATTGCTTTGAACGTTCTCTTTTCATATCATTCTCCTATGTTGTCTATGATCTCCTAGTGGCCTACTTACTCTTCTACATTGCCACCACTTATTTCCACAAGCTTCCATACCCATTTTCCTTCCTTGCTTGGCCAATCTATTGGGCCATCCAAGGCTGCATTCTCACTGGTGTTTGGGTGATTGCTCATGAGTGTGGCCACCATGCCTTCAGCAAGTACCAACTTGTTGATGACATGGTTGGTTTGACCCTTCACCCTTGTCTATTAGTTCCTTATTTCTCATGGAAAATCAGCCACCGCCGCCACCACTCCAACACCGGTTCCCTCGACCGCAACGAAGTGTTTGTCCCAAAACCAAAATCAAAGGTATCATGGTATAACAAGTACATGAACAATCCACCAGGGAGGGCTATCTCCCTCTTCATCACACTCACACTAGGATGGCCAT

>28_FAD2_Plate_Plate 01_C04

TTCNAACCCTCCATTCAGTGTTGGCCAACTCAAGAAAGCAATTCCACCACATTGCTTTGAACGTTCTCTTTTCATATCATTCTCCTATGTTGTCTATGATCTCTTAGTGGCCTACTTACCCTTCTACATTGCCACCACTTATTTCCACAAGCTTCCATACCCATTTTCCTTCCTTGCTTGGCCAATCTATTGGGCCATCCAAGGCTGCATTCTCACTGGTGTTTGGGTGATTGCTCATGAGTGTGGCCACCATGCCTTCAGCAAGTACCAACTTGTTGATGACATGGTTGGTTTGACCCTTCACTCTTGTCTATTAGTTCCTTATTACTCATGGAAGATCAGCCACCGCCGCCACCACTCCAACACCGGTTCCCTCGACCGCAACGAAGTGTTTGTCCCAAAACCAAAATCAAAGGTATCATGGTATAACAAGTACATGAACAATCCACCAGGGAGGGCTATCTCCCTCTTCATCACACTCACACTAGGATGGCCAAT

>29_FAD2_Plate_Plate 01_C05

TTNNAACCCTCCATTCAGTGTTGGCCAACTCAAGAAAGCAATTCCACCACATTGCTTTGAACGTTCTCTTTTCATATCATTCTCCTATGTTGTCTATGATCTCTTAATGGCCTACTTACTCTTCTACATTGCCACCACTTATTTCCACCAGCTTCCATACCCATTTTCCTTCCTTGCTTGGCCAATCTATTGGGCCATCCAAGGCTGCATTCTCACTGGTGTTTGGGTGATTGCTCATGAGTGTGGCCACCATGCCTTCAGCAAGTATCAACTTGTTGATGACATGGTTGGTTTGACCCTTCACTCTTGTCTATTAGTCCCTTATTTCTCATGGAAAATCAGCCACCGCCACCTCCACTCCAACACCGGTTCCCTCGACCGCGACTAAGTGTTTGTCCTGAAACCAAAATCAAAGGTATCACGGTATAACAAGTACATGAACAATCCACTAGAGAGGGCTATTTCCCTTTTCATCACACTCACACTAGGATGGCCAAT

>30_FAD2_Plate_Plate 01_C06

TTCAAACCCTCCATTCAGTGTTGGCCAACTCAAGAAAGCAATTCCACCACATTGCTTTGAACGTTCTCTTTTCATATCATTCTCCTATGTTGTCTATGATCTCTTAATGGCCTACTTACTCTTCTACATTGCCACCACTTATTTCCACCAGCTTCCATACCCATTTTCCTTCCTTGCTTGGCCAATCTATTGGGCCATCCAAGGCTGCATTCTCACTGGTGTTTGGGTGATTGCTCATGAGTGTGGCCACCATGCCTTCAGCAAGTATCAACTTGTTGATGACATGGTTGGTTTGATCCTTCACTCTTGTCTATTAGTCCCTTATTTCTCATGGAAAATCAGCCACCGCCACCTCCACTCCAACACCGGTTCCCTCGACCGCGACTAAGTGTTTGTCCTGAAACCAAAATCAAAGGTATCACGGTATAACAAGTACATGAACAATCCACTAGAGAGGGCTATTTCCCTTTTCATCACACTCACACTAGGATGGCCAAT

>31_FAD2_Plate_Plate 01_C07

TTCAANCCCTCCATTCAGTGTTGGCCAACTCAAGAAAGCAATTCCACCACATTGCTTTGAACGTTCTCTTTTCATATCATTCTCATATGTTGTCTATGATCTCTTAATGGCCTACTTACTCTTCTACATTGCCGCCACTTATTTCCACAAGCTTCCATACCCATTTTCCTTCCTTGCTTGGCCAATCTATTGGGCCATCCAAGGCTGCATTCTCACCGGTGTTTGGGTGATTGCTCATGAGTGTGGCCACCATGCCTTCAGCAAGTACCAACTTGTTGATGACATGGTTGGTTTGACCCTTCACTCTTGTCTATTAGTTCCTTATTTCTCATGGAAAATCAGCCACCGCCGCCACCACTCCAACACAGGTTCCCTCGACCGCGACGAAGTGTTTGTCCCGAAACCAAAATCAAAGGTATCATGGTATAACAAGTACATGAACAATCCACCAGGGAGGGCTATTTCCCTTTTCATCACACTCACACTAGGATGGCCAAT

>33_FAD2_Plate_Plate 01_C09

TTNNAACCCTCCATTCAGTGTTGGCCAACTCAAGAAAGCAATTCCACCACATTGCTTTGAACGTTCTCTTTTCATATCATTCTCCTATGTTGTCTATGATCTCTTAGTGGCCTACTTACTCTTCTACATTGCCACCACTTATTTCCACAAGCTTCCATACCCATTTTCCTTCCTTGCTTGGCCAATCTATTGGGCCATCCAAGGCTGCATTCTCACTGGTGTTTGGGTGATTGCTCATGAGTGTGGCCACCATGCCTTCAGCAAGTACCAACTTGTTGATGACATGGTTGGTTTGACCCTTCACTCTTGTCTATTAGTTCCTTATTTCTCATGGAAAATCAGCCACCGCCGCCACCACTCCAACACCGGTTCCCTCGACCGCAACGAAGTGTTTGTCCCAAAACCAAAATCAAAGGTATCATGGTATAACAAGTACATGAACAATCCACCAGGGAGGGCTATCTCCCTCTTCATCACACTCACACTAGGATGGCCAAT

>34_FAD2_Plate_Plate 01_C10

TTCAANCCCTCCATTCAGTGTTGGCCAACTCAAGAAAGCAATTCCACCACATTGCTTTGAACGTTCTCTTTTCATATCATTCTCATATGTTGTCTATGATCTCTTAATGGCCTACTTACTCTTCTACATTGCCACCACTTATTTCCACAAGCTTCCATACCCATTTTCCTTCCTTGCTTGGCCAATCTATTGGGCCATCCAAGGCTGCATTCTCACCGGTGTTTGGGTGATTGCTCATGAGTGTGGCCATCATGCCTTCAGCAAGTACCAACTTGTTGATGACATGGTTGGTTTGACCCTTCACTCTTGTCTATTAGTTCCTTATTTCTCATGGAAAATCAGCCACCGCCGCCACCACTCCAACACAGGTTCCCTCGACCGCGACGAAGTGTTTGTCCCGAAACCAAAATCAAAGGTATCATGGTATAACAAGTACATGAACAATCCACCAGGGAGGGCTATTTCCCTTTTCATCACACTCACACTAGGATGGCCAAT

>35_FAD2_Plate_Plate 01_C11

TTCNAACCCTCCATTCAGTGTTGGCCAACTCAAGAAAGCAATTCCACCACATTGCTTTGAACGTTCTCTTTTCATATCATTCTCCTATGTTGTCTATGATCTCTTAATGGCCTACTTACTCTTCTACATTGCCACCACTTATTTCCACCAGCTTCCATACCCATTTTCCTTCCTTGCTTGGCCAATCTATTGGGCCATCCAAGGCTGCATTCTCACTGGTGTTTGGGTGATTGCTCATGAGTGTGGCCACCATGCCTTCAGCAAGTATCAACTTGTTGATGACATGGTTGGTTTGATCCTTCACTCTTGTCTATTAGTCCCTTATTTCTCATGGAAAATCAGCCACCGCCACCTCCACTCCAACACCGGTTCCCTCGACCGCGACTAAGTGTTTGTCCTGAAACCAAAATCAAAGGTATCACGGTATAACAAGTACATGAACAATCCACTAGAGAGGGCTATTTCCCTTTTCATCACACTCACACTAGGATGGCCAAT

>36_FAD2_Plate_Plate 01_C12

NNNNANCCCTCCATTCAGTGTTGGCCAACTCAAGAAAGCAATTCCACCACATTGCTTTGAACGTTCTCTTTTCATATCATTCTCATATGTTGTCTATGATCTCTTAATGGCCTACTTACTCTTCTACATTGCCACCACTTATTTCCACAAGCTTCCATACCCATTTTCCTTCCTTGCTTGGCCAATCTATTGGGCCATCCAAGGCTGCATTCTCACCGGTGTTTGGGTGATTGCTCATGAGTGTGGCCACCATGCCTTCAGCAAGTACCAACTTGTTGATGACATGGTTGGTTTGACCCTTCACTCTTGTCTATTAGTTCCTTATTTCTCATGGAAAATCAGCCACCGCCGCCACCACTCCAACACAGGTTCCCTCGACCGCGACGAAGTGTTTGTCCCGAAACCAAAATCAAAGGTATCATGGTATAACAAGTACATGAACAATCCACCAGGGAGGGCTATTTCCCTTTTCATCACACTCACACTAGGATGGCCAAT

>37_FAD2_Plate_Plate 01_D01

TTCNAACCCTCCATTCGGTGTTGGCCAACCCAAGAAAGCAATTCCACCACATTGCTTTGAACGTTCTCTTTTCATATCATTCTCATATGTTGTCTATGATCTCTTAATGGCCTACTTACTCTTCTACATTGCCACCACTTATTTCCACAAGCTTCCATACCCATTTTCCTTCCTTGCTTGGCCAATCTATTGGGCCATCCAAGGCTGCATTCTCACCGGTGTTTGGGTGATTGCTCATGAGTGTGGCCACCATGCCTTCAGCAAGTACCAACTTGTTGATGACATGGTTGGTTTGACCCTTCACTCTTGTCTATTAGTTCCTTATTTCTCATGGAAAATCAGCCACCGCCGCCACCACTCCAACACAGGTTCCCTCGACCGCGACGAAGTGTTTGTCCCGAAACCAAAATCAAAGGTATCATGGTATAACAAGTACATGAACAATCCACCAGGGAGGGCTATTTCCCTTTTCATCACACTCACACTAGGATGGCCAAT

>38_FAD2_Plate_Plate 01_D02

TTCNAACCCTCCATTCAGTGTTGGCCAACTCAAGAAAGCAATTCCACCACATTGCTTTGAACGTTCTCTTTTCATATCATTCTCATATGTTGTCTATGATCTCTTAATGGCCTACTTACTCTTCTACATTGCCACCACTTATTTCCACAAGCTTCCATACCCATTTTCCTTCCTTGCTTGGCCAATCTATTGGGCCATCCAAGGCTGCATTCTCACCGGTGTTTGGGTGATTGCTCATGAGTGTGGCCACCATGCCTTCAGCAAGTACCAACTTGTTGATGACATGGTTGGTTTGACCCTTCACTCTTGTCTATTAGTTCCTTATTTCTCATGGAAAATCAGCCACCGCCGCCACCACTCCAACACAGGTTCCCTCGACCGCGACGAAGTGTTTGTCCCGAAACCAAAATCAAAGGTATCATGGTATAACAAGTACATGAACAATCCACCAGGGAGGGCTATTTCCCTTTTCATCACACTCACACTAGGATGGCCAAT

>39_FAD2_Plate_Plate 01_D03

TTCNAACCCTCCATTCAGTGTTGGCCAACTCAAGAAAGCAATTCCACNACATTGCTTTGAACGTTCTCTTTTCATATCATTCTCATATGTTGTCTATGATCTCTTAATGGCCTACTTACTCTTCTACATTGCCACCACTTATTTCCACAAGCTTCCATACCCATTTTCCTTCCTTGCTTGGCCAATCTATTGGGCCATCCAAGGCTGCATTCTCACCGGTGTTTGGGTGATTGCTCATGAGTGTGGCCACCATGCCTTCAGCAAGTACCAACTTGTTGATGACATGGTTGGTTTGACCCTTCACTCTTGTCTATTAGTTCCTTATTTCTCATGGAAAATCAGCCACCGCCGCCACCACTCCAACACAGGTTCCCTCGACCGCGACGAAGTGTTTGTCCCGAAACCAAAATCAAAGGTATCATGGTATAACAAGTACATGAACAATCCACCAGGGAGGGCTATTTCCCTTTTCATCACACTCACACTAGGATGGCCAAT

>40_FAD2_Plate_Plate 01_D04

TTCNAACCCTCCATTCAGTGTTGGCCAACTCAAGAAAGCAATTCTACCACATTGCTTTGAACGTTCTCTTTTCATATCATTCTCCTATGTTGTCTATGATCTCTTAGTGGCCTACTTACCCTTCTACATTGCCACCACTTATTTCCACAAGCTTCCATACCCATTTTCCTTCCTTGCTTGGCCAGTCTATTGGGCCATCCAAGGCTGCATTCTCACTGGTGTTTGGGTGATTGCTCATGAGTGTGGCCACCATGCCTTCAGCAAGTACCAACTTGTTGATGACATGGTTGGTTTGACCCTTCACTCTTGTCTATTAGTTCCTTATTTCTCATGGAAAATCAGCCACCGCCGCCACCACTCCAACACCGGTTCCCTCGACCGCAACGAAGTGTTTGTCCCAAAACCAAAATCAAAGGTATCATGGTATAACAAGTACATGAACAATCCACCAGGGAGGGCTATCTCCCTCTTCATCACACTCACACTAGGATGGCCAAT

>41_FAD2_Plate_Plate 01_D05

TTNAAACCCTCCATTCAGTGTTGGCCAACTCAAGAAAGCAATCCCACCACATTGCTTTGAACGTTCTCTTTTCATATCATTCTCCTATGTTGTCTATGATCTCTTAGTGGCCTACTTACTCTTCTACATTGCCACCACTTATTTCCACAAGCTTCCATACCCATTTTCCTTCCTTGCTTGGCCAATCTATTGGGCCATCCAAGGCTGCATTCTCACTGGTGTTTGGGTGATTGCTCATGAGTGTGGCCACCATGCCTTCAGCAAGTACCAACTTGTTGATGACATGGTTGGTTTGACCCTTCACTCTTGTCTATTAGTTCCTTATTTCTCATGGAAAATCAGCCACCGCCGCCACCACTCCAACACCGGTTCCCTCGACCGCAACGAAGTGTTTGTCCCAAAACCAAAATCAAAGGTATCATGGTATAACAAGTACATGAACAATCCACCAGGGAGGGCTATCTCCCTCTTCATCACACTCACACTAGGATGGNCAAT

>42_FAD2_Plate_Plate 01_D06

TTCNAACCCTCCATTCAGTGTTGGCCAACTCAAGAAAGCAATTCCACCACATTGCTTTGAACGTTCTCTTTTCATATCATTCTCCTATGTTGTCTATGATCTCTTAGTGGCCTACTTACTCTTCTACATTGCCACCACTTATTTCCACAAGCTTCCATACCCATTTTCCTTCCTTGCTTGGCCAATCTATTGGGCCATCCAAGGCTGCATTCTCACTGGTGTTTGGGTGATTGCTCATGAGTGTGGCCACCATGCCTTCAGCAAGTACCAACTTGTTGATGACATGGTTGGTTTGACCCTTCACTCTTGTCTATTAGTTCCTTATTTCTCATGGAAAATCAGCCACCGCCGCCACCACTCCAACACCGGTTCCCTCGACCGCAACGAAGTGTTTGTCCCAAAACCAAAATCAAAGGTATCATGGTATAACAAGTACATGAACAATCCACCAGGGAGGGCTATCTCCCTCTTCATCACACTCACACTAGGATGGCCAAT

>43_FAD2_Plate_Plate 01_D07

NNNNAACCCTCCATTCAGTGTTGGCCAACTCAAGAAAGCAATTCCACCNCATTGCTTTGAACGTTCTCTTTTCATATCATTCTTATATGTTGTCTATGATCTCTTAATGGCCTACTTACTCTTCTACATTGCCACCACTTATTTCCACAAGCTTCCATACCCATTTTCCTTCCTTGCTTGGCCAATCTATCGGGCCATCCAAGGCTGCATTCTCACCGGTGTTTGGGTGATTGCTCATGAGTGTGGCCACCACGCCTTCAGCAAGTACCAACTTGTTGATGACATGGTTGGTTTGACCCTTCACTCTTGTCTATTAGTTCCTTATTTCTCATGGAAAATCANCCACCGCCGCCACCACTCCAACACAGGTTCCCTCGACCGCGACGAAGTGTTTGTCCCGAAACCAAAATCAAAGGTATCATGGTATAACANNNNCTNAATCAATNNNCNAAGNNGNNNTAN

>44_FAD2_Plate_Plate 01_D08

TTCAANCCCTCCATTCAGTGTTGGCCAACTCAAGAAAGCAATTCCACCACATTGCTTTGAACGTTCTCTTTTCATATCATTCTCCTATGTTGTCTATGATCTCTTAATGGCCTACTTACTCTTCTACATTGCCACCACTTATTTCCACCAGCTTCCATACCCATTTTCCTTCCTTGCTTGGCCAATCTATTGGGCCATCCAAGGCTGCATTCTCACTGGTGTTTGGGTGATTGCTCATGAGTGTGGCCACCATGCCTTCAGCAAGTATCAACTTGTTGATGACATGGTTGGTTTGATCCTTCACTCTTGTCTATTAGTCCCTTATTTCTCATGGAAAATCAGCCACCGCCACCTCCACTCCAACACCGGTTCCCTCGACCGCGACTAAGTGTTTGTCCTGAAACCAAAATCAAAGGTATCACGGTATAACAAGTACATGAACAATCCACTAGAGAGGGCTATTTCCCTTTTCATCACACTCACACTAGGATGGCCAAT

>45_FAD2_Plate_Plate 01_D09

TTCAAACCCTCCATTCAGTGTTGGCCAACTCAAGAAAGCAATTCCACCACATTGCTTTGAACGTTCTCTTTCCATATCATTCTTCTATGTTGTCTATGATCTCTTAATGGCCTACTTACTCTTCTACATTGCCACCACTTATTTCCACCAGCTTCCATACCCATTTTCCTTCCTTGCTTGGCCAATCTATTGGGCCATCCAAGGCTGCATTCTCACTGGTGTTTGGGTGATTGCTCATGAGTGTGGCCACCATGCCTTCAGCAAGTATCAACTTGCTGATGACATGGTTGGTTTGATCCTTCACTCTTGTCTATTAGTCCCTTATTTCTCATGGAAAATCAGCCACCGCCACCTCCACTCCAACACCGGTTCCCTCGACCGCGACTAAGTGTTTGTCCTGAAACCAAAATCAAAGGTATCACGGTATAACAAGTACATGAACAATCCACTAGAGAGGGCTATTTCCCTTTTCATCACACTCACACTAGGATGGCCAAT

>47_FAD2_Plate_Plate 01_D11

TNNNNACCCTCCNTTCAGTGTTGGCCAACTCAAGAAAGCAATTCCACCACATTGCTTTGAACGTTCTCTTTTCATATCATTCTCCTATGTTGTCTATGATCTCTTAGTGGCCTACTTACTCTTCTACATTGCCACCACTTATTTCCACAAGCTTCCATACCCATTTTCCTTCCTTGCTTGGCCAATCTATTGGGCCATCCAAGGCTGCATTCTCACTGGTGTTTGGGTGATTGCTCATGAGTGTGGCCACCATGCCTTCAGCAAGTACCAACTTGTTGATGACATGGTTGGTTTGACCCTTCACTCTTGTCTATTAGTTCCTTATTTCTCATGGAAAATCAGCCACCGCCGCCACCACTCCAACACCGGTTCCCTCGACCGCAACGAAGTGTTTGTCCCAAAACCAAAATCAAAGGTATCATGGTATAACAAGTACATGAACAATCCACCAGGGAGGGCTATCTCCCTCTTCATCACACTCACACTAGGATGGCCAAT

>48_FAD2_Plate_Plate 01_D12

TTCAAACCCTCCATTCAGTGTTGGCCAACTCAAGAAAGCAATTCCACCACATTGCTTTGGACGTTCTCTTTTCATATCATTCTCCTATGTTGTCTATGATCTCTTAATGGCCTACTTACTCTTCTACATTGCCACCACTTATTTCCACCAGCTTCCATACCCATTTTCCTTCCTTGCTTGGCCAATCTATTGGGCCATCCAAGGCTGCATTCTCACTGGTGTTTGGGTGATTGCTCATGAGTGTGGCCACCATGCCTTCAGCAAGTATCAACTTGTTGATGACATGGTTGGTTTGATCCTTCACTCTTGTCTATTAGTCCCTTATTTCTCATGGAAAATCAGCCACCGCCACCTCCACTCCAACACCGGTTCCCTCGACCGCGACTAAGTGTTTGTCCTGGAACCAAAATCAAAGGTATCACGGTATAACAAGTACATGAACAATCCACTAGAGAGGGCTATTTCCCTTTTCATCACACTCACACTAGGATGGCCAAT

>50_FAD2_Plate_Plate 01_E02

TTCAAACCCTCCATTCAGTGTTGGCCAACTCAAGAAAGCAATTCCACCACATTGCTTTGAACGTTCTCTTTTCATATCATTCTCCTATGTTGTCTATGATCTCTTAGTGGCCTACTTACTCTTCTACATTGCCACCACTTATTTCCACAAGCTTCCATACCCATTTTCCTTCTTTGCTTGGCCAATCTATTGGGCCATCCAAGGCTGCATTCTCACTGGTGTTTGGGTGATTGCTCATGAGTGTGGCCACCATGCCTTCAGCAAGTACCAACTTGTTGATGACATGGTTGGTTTGACCCTTCACTCTTGTCTATTAGTTCCTTATTTCTCATGGAAAATCAGCCACCGCCGCCACCACTCCAACACCGGTTCCCTCGACCGCAACGAAGTGTTTGTCCCAAAACCAAAANCAAAGGTATCATGGTATAACAAGTACATGAACAATCCACCAGGGAGGGCTATCTCCCTCTTCATCACANTCACACTAGGATGGCCAAT

>51_FAD2_Plate_Plate 01_E03

TTCAAACCCTCCATTCAGTGTTGGCCAACTCAAGAAAGCAATTCCACCACATTGCTTTGAACGTTCTCTTTTCATATCATTCTCATATGTTGCCTATGATCTCTTAATGGCCTACTTACTCTTCTACATTGCCACCACTTATTTCCACAAGCTTCCATACCCATTTTCCTTCCTTGCTTGGCCAATCTATTGGGCCATCCAAGGCTGCATTCTCACCGGTGTTTGGGTGATTGCTCATGAGTGTGGCCACCATGCCTTCAGCAAGTACCAACTTGTTGATGACATGGTTGGTTTGACCCTTCACTCTTGTCTATTAGTTCCTTATTTCCCATGGAAAATCAGCCACCGCCGCCACCACTCCAACACAGGTTCCCTCGACCGCGACGAAGTGTTTGTCCCGAAACCAAAATCAAAGGTATCATGGTATAACAAGTACATGAACAATCCACCAGGGAGGGCTATTTCCCTTTTCATCACACTCACACTAGGATGGCCAAT

>53_FAD2_Plate_Plate 01_E05

TTCNAACCCTCCATTCAGTGTTGGCCAACTCAAGAAAGCAATTCCACCACATTGCTTTGAACGTTCTCTTTTCATATCATTCTCCTATGTTGTCTATGATCTCTTAGTGGCCTACTTACTCTTCTACATTGCCACCACTTATTTCCACAAGCTTCCATACCCATTTTCCTTCCTTGCTTGGCCAATCTATTGGGCCATCCAAGGCTGCATTCTCACTGGTGTTTGGGTGATTGCTCATGAGTGTGGCCACCATGCCTTCAGCAAGTACCAACTTGTTGATGACATGGTTGGTTTGACCCTTCACTCTTGTCTATTAGTTCCTTATTTCTCATGGAAAATCAGCCACCGCCGCCACCACTCCAACACCGGTTCCCTCGACCGCAACGAAGTGTTTGTCCCAAAACCAAAATCAAAGGTATCATGGTATAACAAGTACATGAACAATCCACCAGGGAGGGCTATCTCCCTCTTCATCACACTCACACTAGGATGGCCAAT

>54_FAD2_Plate_Plate 01_E06

TTCNAACCCTCCATTCAGTGTTGGCCAACTCAAGAAAGCAATTCCACCACATTGCTTTGAACGTTCTCTTTTCATATCATTCTCATATGTTGTCTATGATCTCTTAATGGCCTACTTACTCTTCTACATTGCCACCACTTATTTCCACAAGCTTCCATACCCATTTTCCTTCCTTGCTTGGCCAATCTATTGGGCCATCCAAGGCTGCATTCTCACCGGTGTTTGGGTGATTGCTCATGAGTGTGGCCACCATGCCTTCAGCAAGTACCAACTTGTTGATGACATGGTTGGTTTGACCCTTCACTCTTGTCTATTAGTTCCTTATTTCTCATGGAAAATCAGCCACCGCCGCCACCACTCCAACACAGGTTCCCTCGACCGCGACGAAGTGTTTGTCCCGAAACCAAAATCAAAGGTATCATGGTATAACAAGTACATGAACAATCCACCAGGGAGGGCTATTTCCCTTTTCATCACACTCACACTAGGATGGNCAAN

>56_FAD2_Plate_Plate 01_E08

TTCNAACCCTCCATTCAGTGTTGGCCAACTCAAGAAAGCAATTCCACCACATTGCTTTGAACGTTCTCTTTTCATATCATTCTCCTATGTTGTCTATGATCTCTTAGTGGCCTACTTACTCTTCTACATTGCCACCACTTATTTCCACAAGCTTCCATACCCATTTTCCTTCCTTGCTTGGCCAATCTATTGGGCCATCCAAGGCTGCATTCTCACTGGTGTTTGGGTGATTGCTCATGAGTGTGGCCACCATGCCTTCAGCAAGTACCAACTTGTTGATGACATGGTTGGTTTGACCCTTCACTCTTGTCTATTAGTTCCTTATTTCTCATGGAAAATCAGCCACCGCCGCCACCACTCCAACACCGGTTCCCTCGACCGCAACGAAGTGTTTGTCCCAAAACCAAAATCAAAGGTATCATGGTATAACAAGTACATGAACAATCCACCAGGGAGGGCTATCTCCCTCTTCATCACACTCACACTAGGATGGCCAAT

>57_FAD2_Plate_Plate 01_E09

TTCNAACCCTCCATTCAGTGTTGGCCAACTCAAGAAAGCAATTCCACCACATTGCCTTGAACGTTCTCTTTTCATATCATTCTCATATGTTGTCTATGATCTCTTAATGGCCTACTTACTCTTCTACATTGCCACCACTTATTTCCACAAGCTTCCATACCCATTTTCCTTCCTTGCTTGGCCAATCTATTGGGCCATCCAAGGCTGCATTCTCACCGGTGTTTGGGTGATTGCTCATGAGTGTGGCCACCATGCCTTCAGCAAGTACCAACTTGTTGATGACATGGTTGGTTTGACCCTTCACTCTTGTCTATTAGTTCCTTATTTCTCATGGAAAATCAGCCACCGCCGCCACCACTCCAACACAGGTTCCCTCGACCGCGACGAAGTGTTTGTCCCGAAACCAAAATCAAAGGTATCATGGTATAACAAGTACATGAACAATCCACCAGGGAGGGCTATTTCCCTTTTCATCACACTCACACTAGGATGGCCAAT

>59_FAD2_Plate_Plate 01_E11

TTNNNNCCCTCCATTCAGTGTTGGCCAACTCAAGAAAGCAATTCCACCACATTGCTTTGAACGTTCTCTTTTCATATCATTCTCCTATGTTGTCTATGATCTCTTAATGGCCTACTTACTCTTCTACATTGCCACCACTTATTCCCACCAGCTTCCATACCCATTTTCCTTCCTTGCTTGGCCAATCTATTGGGCCATCCAAGGCTGCATTCTCACTGGTGTTTGGGTGATTGCTCATGAGTGTGGCCACCATGCCTTCAGCAAGTATCAACTTGTTGATGACATGGTTGGTTTGATCCTTCACTCTTGTCTATTAGTCCCTTATTTCTCATGGAAAATCAGCCACCGCCACCTCCACTCCAACACCGGTTCCCTCGACCGCGACTAAGTGTTTGTCCTGAAACCAAAATCAAAGGTATCACGGTATAACAAGTACATGAACAATCCACTAGAGAGGGCTATTTCCCTTTTCATCACACTCACACTAGGATGGCCAAT

>60_FAD2_Plate_Plate 01_E12

TTCNAACCCTCCATTCAGTGTTGGCCAACTCAAGAAAGCAATTCCACCACATTGCTTTGAACGTTCTCTTTTCATATCATTCTCATATGTTGTCTATGATCTCTTAATGGCCTACTTACTCTTCTACATTGCCACCACTTATTTCCACCAGCTTCCATACCCATTTTCCTTCCTTGCTTGGCCAATCTATTGGGCCATCCAAGGCTGCATTCTCACTGGTGTTTGGGTGATTGCTCATGAGTGTGGCCACCATGCCTTCAGCAAGTATCAACTTGTTGATGACATGGTTGGTTTGATCCTTCACTCTTGTCTATTAGTCCCTTATTTCTCATGGAAAATCAGCCACCGCCACCTCCACTCCAACACCGGTTCCCTCGACCGCGACTAAGTGTTTGTCCTGAAACCAAAATCAAAGGTATCACGGTATAACAAGTACATGAACNATCCATTANAGAGGGAAANTC

>61_FAD2_Plate_Plate 01_F01

TTCNAACCCTCCATTCAGTGTTGGCCAACTCAAGAAAGCAATTCCACCACATTGCTTTGAACGTTCTCTTTTCATATCATTCTCATATGTTGTCTATGATCTCTTAATGGCCTACTTACTCTTCTACATTGCCACCACTTATTTCCACAAGCTTCCATACCCATTTTCCTTCCTTGCTTGGCCAATCTATTGGGCCATCCAAGGCTGCATTCTCACCGGTGTTTGGGTGATTGCTCATGAGTGTGGCCACCATGCCTTCAGCAAGTACCAACTTGTTGATGACATGGTTGGTTTGACCCTTCACTCTTGTCTATTAGTTCCTTATTTCTCATGGAAAATCAGCCACCGCCGCCACCACTCCAACACAGGTTCCCTCAGACCGCGACGAAGTGTTTGTCCCGAAACCAAAATCAAAGGTATCATGGTATAACAAGTACATGAACAATCCACCAGGGAGGGCTATTTCCCTTTTCATCACACTCACACTAGGATGGCCAAT

>62_FAD2_Plate_Plate 01_F02

TTCNAACCCTCCATTCAGTGTTGGCCAACTCAAGAAAGCAATTCCACCACATTGCTTTGAACGTTCTCTTTTCATATCATTCTCCTATGTTGTCTATGATCTCTTAGTGGCCTACTTACTCTTCTACATTGCCACCACTTATTTCCACAAGCTTCCATACCCATTTTCCTTCCTTGCTTGGCCAATCTATTGGGCCATCCAAGGCTGCATTCTCACTGGTGTTTGGGTGATTGCTCATGAGTGTGGCCACCATGCCTTCAGCAAGTACCAACTTGTTGATGACATGGTTGGTTTGACCCTTCACTCTTGTCTATTAGTTCCTTATTTCTCATGGAAAATCAGCCACCGCCGCCACCACTCCAACACCGGTTCCCTCGACCGCAACGAAGTGTTTGTCCCAAAACCAAAATCAAAGGTATCATGGTATAACAAGTACATGAACAATCCACCAGGGAGGGCTATCTCCCTCTTCATCACACTCACACTAGGATGGCCAAT

>63_FAD2_Plate_Plate 01_F03

TTCNAACCCTCCATTCAGTGTTGGCCAACTCAAGAAAGCAATTCCACCACATTGCTTTGAACGTTCTCTTTTCATATCATTCTCCTATGTTGTCTATGATCTCTTAATGGCCTACTTACTCTTCTACATTGCCACCACTTATTTCCACCAGCTTCCATACCCATTTTCCTTCCTTGCTTGGCCAATCTATTGGGCCATCCAAGGCTGCATTCTCACTGGTGTTTGGGTGATTGCTCATGAGTGTGGCCACCATGCCTTCAGCAAGTATCAACTTGTTGATGACATGGTTGGTTTGATCCTTCACTCTTGTCTATTAGTCCCTTATTTCTCATGGAAAATCAGCCACCGCCACCTCCACTCCAACACCGGTTCCCTCGACCGCGACTAAGTGTTTGTCCTGAAACCAAAATCAAAGGTATCACGGTATAACAAGTACATGAACAATCCACTAGAGAGGGCTATTTCCCTTTTCATCACACTCACACTAGGATGGCCAAT

>66_FAD2_Plate_Plate 01_F06

TTCNAACCCTCCATTCAGTGTTGGCCAACTCAAGAAAGCAATTCCACCACATTGCTTTGAACGTTCTCTTTTCATATCATTCTCCTATGTTGTCTATGATCTCTTAGTGGCCTACTTACTCTTCTACATTGCCACCACTTATTTCCACAAGCTTCCATACCCATTTTCCTTCCTTGCTTGGCCAATCTATTGGGCCATCCAAGGCTGCATTCTCACTGGTGTTTGGGTGATTGCTCATGAGTGTGGCCACCATGCCTTCAGCAAGTACCAACTTGTTGATGACATGGTTGGTTTGACCCTTCACTCTTGTCTATTAGTTCCTTATTTCTCATGGAAAATCAGCCACCGCCGCCACCACTCCAACACCGGTTCCCTCGACCGCAACGAAGTGTTTGTCCCAAAACCAAAATCAAAGGTATCATGGTATAACAAGTACATGAACAATCCACCAGGGAGGGCTATCTCCCTCTTCATCACACTCACACTAGGATGGCCAAT

>67_FAD2_Plate_Plate 01_F07

TTNNANCCNTCCATTCAGTGTTGGCCAACTCAAGAAAGCAATTCCACCACATTGCTTTGAACGTTCTCTTTTCATATCATTCTCATATGTTGTCTATGATCTCTTAATGGCCTACTTACTCTTCTACATTGCCACCACTTATTTCCACAAGCTTCCATACCCATTTTCCTTCCTTGCTTGGCCAATCTATTGGGCCATCCAAGGCTGCATTCTCACCGGTGTTTGGGTGATTGCTCATGAGTGTGGCCACCATGCCTTCAGCAAGTACCAACTTGTTGATGACATGGTTGGTTTGACCCTTCACTCTTGTCTATTAGTTCCTTATTTCTCATGGAAAATCAGCCACCGCCGCCACCACTCCAACACAGGTTCCCTCAGACCGCGACGAAGTGTTTGTCCCGAAACCAAAATCAAAGGTATCATGGTATAACAAGTACATGAACAATCCACCAGGGAGGGCTATTTCCCTTTTCATCACACTCACACTAGGATGGCCAAT

>70_FAD2_Plate_Plate 01_F10

TNNAAACCCTCCATTCAGTGTTGGCCAACTCAAGAAAGCAATTCCACCACATTGCTTTGAACGTTCTCTTTTCATATCATTCTCCTATGTTGTCTATGATCTCTTAGTGGCCTACTTACTCTTCTACATTGCCACCACTTATTTCCACAAGCTTCCATACCCATTTTCCTTCCTTGCTTGGCCAATCTATTGGGCCATCCAAGGCTGCATTCTCACTGGTGTTTGGGTGATTGCTCATGAGTGTGGCCACCATGCCTTCAGCAAGTACCAACTTGTTGATGACATGGTCGGTTTGACCCTTCACTCTTGTCTATTAGTTCCTTATTTCTCATGGAAAATCAGCCACCGCCGCCACCACTCCAACACCGGTTCCCTCGACCGCAACGAAGTGTTTGTCCCAAAACCAAAATCAAAGGTATCATGGTATAACAAGTACATGAACAATCCACCAGGGAGGGCTATCTCCCTCTTCATCACACTCACACTAGGATGGCCAAT

>71_FAD2_Plate_Plate 01_F11

TTNNAACCCTCCATTCANTGTTGGCCAACTCAAGAAAGCAATTCCACCACATTGCTTTGAACGTTCTCTTTTCATATCATTCTCCTATGTTGTCTATGATCTCTTAGTGGCCTACTTACTCTTCTACATTGCCACCACTTATTTCCACAAGCTTCCATACCCATTTTCCTTCCTTGCTTGGCCAATCTATTGGGCCATCCAAGGCTGCATTCTCACTGGTGTTTGGGTGATTGCTCATGAGTGTGGCCACCATGCCTTCAGCAAGTACCAACTTGTTGATGACATGGTTGGTTTGACCCTTCACTCTTGTCTATTAGTTCCTTATTTCTCATGGAAAATCAGCCACCGCCGCCACCACTCCAACACCGGTTCCCTCGACCGCAACGAAGTGTTTGTCCCAAAACCAAAATCAAAGGTATCGTGGTATAACAAGTACATGAACAATCCACCAGGGAGGGCTATCTCCCTCTTCATCACACTCACACTANGATGGCCAAT

>72_FAD2_Plate_Plate 01_F12

TTCNAACCCTCCATTCAGTGTTGGCCAACTCAAGAAAGCAATTCCACCACATTGCTTTGAACGTTCTCTTTTCATATCATTCTCCTATGTTGTCTATGATCTCTTAATGGCCTACTTACTCTTCTACATTGCCACCACTTATTTCCACCAGCTTCCATACCCATTTTCCTTCCTTGCTTGGCCAATCTATTGGGCCATCCAAGGCTGCATTCTCACTGGTGTTTGGGTGATTGCTCATGAGTGTGGCCACCATGCCTTCAGCAAGTATCAACTTGTTGATGACATGGTTGGTTTGATCCTTCACTCTTGTCTATTAGTCCCTTATTTCTCATGGAAAATCAGCCACCGCCACCTCCACTCCAACACCGGTTCCCTCGACCGCGACTAAGTGTTTGTCCTGAAACCAAAATCAAAGGTATCACGGTATAACAAGTACATGAACAATCCACTAGAGAGGGCTATTTCCCTTTTCATCACACTCACACTAGGATGGCCAAT

>73_FAD2_Plate_Plate 01_G01

TTCAANCCCTCCATTCAGTGTTGGCCAACTCAAGAAAGCAATTCCACCACATTGCTTTGAACGTTCTCTTTTCATATCATTCTCCTATGTTGTCTATGATCTCTTAGTGGCCTACTTACTCTTCTACATTGCCACCACTTATTTCCACAAGCTTCCATACCCATTTTCCTTCCTTGCTTGGCCAATCTATTGGGCCATCCAAGGCTGCATTCTCACTGGTGTTTGGGTGATTGCTCATGAGTGTGGCCACCATGCCTTCAGCAAGTACCAACTTGTTGATGACATGGTTGGTTTGACCCTTCACTCTTGTCTATTAGTTCCTTATTTCTCATGGAAAATCAGCCACCGCCGCCACCACTCCAACACCGGTTCCCTCGACCGCAACGAAGTGTTTGTCCCAAAACCAAAATCAAAGGCATCATGGTATAACAAGTACATGAACAATCCACCAGGGAGGGCTATCTCCCTCTTCATCACACTCACACTAGGATGGCCAAT

>74_FAD2_Plate_Plate 01_G02

TTCAANCCCTCCATTCAGTGTTGGCCAACTCAAGAAAGCAATTCCACNACATTGCTTTGAACGTTCTCTTTTCATATCATTCTCCTATGTTGTCTATGATCTCTTAGTGGCCTACTTACTCTTCTACATTGCCACCACTTATTTCCACAAGCTTCCATACCCATTTTCCTTCCTTGCTTGGCCAATCTATTGGGCCATCCAAGGCTGCATTCTCACTGGTGTTTGGGTGATTGCTCATGAGTGTGGCCACCATGCCTTCAGCAAGTACCAACTTGTTGATGACATGGTTGGTTTGACCCTTCACTCTTGTCTATTAGTTCCTTATTTCTCATGGAAAATCAGCCACCGCCGCCACCACTCCAACACCGGTTCCCTCGACCGCAACGAAGTGTTTGTCCCAAAACCAAAATCAAAGGTATCATGGTATAACAAGTACATGAACAATCCACCAGGGAGGGCTATCTCCCTCTTCATCACACTCACACTAGGATGGCCAAT

>76_FAD2_Plate_Plate 01_G04

TTCNAACCCTCCATTCAGTGTTGGCCAACTCAAGAAAGCAATTCCACCACATTGCTTTGAACGTTCTCTTTTCATATCATTCTCCTATGTTGTCTATGATCTCTTAATGGCCTACTTACTCTTCTACATTGCCACCACTTATTTCCACCAGCTTCCATACCCATTTTCCTTCCTTGCTTGGCCAATCTATTGGGCCATCCAAGGCTGCATTCTCACTGGTGTTTGGGTGATTGCTCATGAGTGTGGCCACCATGCCTTCAGCAAGTATCAACTTGTTGATGACATGGTTGGTTTGATCCTTCACTCTTGTCTATTAGTCCCTTATTTCTCATGGAAAATCAGCCACCGCCACCTCCACTCCAACACCGGTTCCCTCGACCGCGACTAAGTGTTTGTCCTGAAACCAAAATCAAAGGTATCACGGTATAACAAGTACATGAACAATCCACTAGAGAGGGCTATTTCCCTTTTCATCACACTCACACTAGGATGGCCAAT

>77_FAD2_Plate_Plate 01_G05

ATTNNACCCTCCATTCAGTGTTGGCCAACTCAAGAAAGCAATTCCACCACATTGCTTTGAACGTTCTCTTTTCATATCATTCTCCTATGTTGTCTATGATCTCTTAGTGGCCTACTTACTCTTCTACATTGCCACCACTTATTTCCACAAGCTTCCATACCCATTTTCCTTCCTTGCTTGGCCAATCTATTGGGCCATCCAAGGCTGCATTCTCACTGGTGTTTGGGTGATTGCTCATGAGTGTGGCCACCATGCCTTCAGCAAGTACCAACTTGTTGATGACATGGTTGGTTTGACCCTTCACTCTTGTCTATTAGTTCCTTATTTCTCATGGAAAGTCAGCCACCGCCGCCACCACTCCAACACCGGTTCCCTCGACCGCAACGAAGTGTTTGTCCCAAAACCAAAATCAAAGGTATCATGGTATAACAAGTACATGAACAATCCACCAGGGAGGGCTATCTCCCTCTTCATCACACTCACACTAGGATGGCCAAT

>79_FAD2_Plate_Plate 01_G07

NTNNAACCCTCCATTCAGTGTTGGCCAACTCAAGAAAGCAATTCCACCACATTGCTTTGAACGTTCTCTTTTCATATCATTCTCCTATGTTGTCTATGATCTCTTAGTGGCCTACTTACTCTTCTACATTGCCACCACTTATTTCCACAAGCTTCCATACCCATTTTCCTTCCTTGCTTGGCCAATCTATTGGGCCATCCAAGGCTGCATTCTCACTGGTGTTTGGGTGATTGCTCATGAGTGTGGCCACCATGCCTTCAGCAAGTACCAACTTGTTGATGACATGGTTGGTTTGACCCTTCACTCTTGTCTATTAGTTCCTTATTTCTCATGGAAAATCAGCCACCGCCGCCACCACTCCAACACCGGTTCCCTCGACCGCAACGAAGTGTTTGTCCCAAAACCAAAATCAAAGGTATCATGGTATAACAAGTACATGAACAATCCACCAGGGAGGGCTATCTCCCTCTTCATCACACTCACACTAGGATGGCCAAT

>80_FAD2_Plate_Plate 01_G08

TTNNAACCCTCCATTCAGTGTTGGNCNACTCAAGAAAGCAATTCCACCACATTGCTTTGAACGTTCTCTTTTCATATCATTCTCCTATGTTGTCTATGATCTCTTAGTGGCCTACTTACTCTTCTACATTGCCACCACTTATTTCCACAAGCTTCCATACCCATTTTCCTTCCTTGCTTGGCCAATCTATTGGGCCATCCAAGGCTGCATTCTCACCGGTGTTTGGGTGATTGCTCATGAGTGTGGCCACCATGCCTTCAGCAAGTACCAACTTGTTGATGACATGGTTGGTTTGACCCTTCACTCTTGTCTATTAGTTCCTTATTTCTCATGGAAAATCAGCCACCGCCGCCACCACTCCAACACAGGTTCCCTCAGACCGCGACGAAGTGTTTGTCCCGAAACCAAAATCAAAGGTATCATGGTATAACAAGTACATGAACAATCCACCAGGGAGGGCTATTTCCCTTTTCATCACACTCACACTAGGATGGCCAAT

>58_FAD2_Plate_Plate 01_E10

TTCAANCCCTCCATTCAGTGTTGGCCAACTCAAGAAAGCAATTCCACCACATTGCTTTGAACGTTCTCTTTTCATATCATTCTCCTATGTTGTCTATGATCTCTTAATGGCCTACTTACTCTTCTACATTGCCGCCACTTATTTCCACCAGCTTCCATACCCATTTTCCTTCCTTGCTTGGCCAATCTATTGGGCCATCCAAGGCTGCATTCTCACTGGTGTTTGGGTGATTGCTCATGAGTGTGGCCACCATGCCTTCAGCAAATATCAACTTGTTGATGACATGGTTGGTTTGATCCTTCACTCTTGTCTATTAGTCCCTTATTTCTCATGGAAAATCAGCCACCGCCACCTCCACTCCAACACCGGTTCCCTCGACCGCGACTAAGTGTTTGTCCTGAAACCAAAATCAAAGGTATCACGGTATAACAAGTACATGAACAATCCACTAGAGAGGGCTATTTCCCTTTTCATCACACTCACACTAGGATGGCCAAT

>59_FAD2_Plate_Plate 01_E11

TTCNAACCCTCCATTCAGTGTTGGCCAACTCAAGAAAGCAATTCCACCACATTGCTTTGAACGTTCTCTTTTCATATCATTCTCCTATGTTGTCTATGATCTCTTAGTGGCCTACTTACTCTTCTACATTGCCACCACTTATTTCCACAAGCTTCCATACCCATTTTCCTTCCTTGCTTGGCCAATCTATTGGGCCATCCAAGGCTGCATTCTCACTGGTGTTTGGGTGATTGCTCATGAGTGTGGCCACCATGCCTTCAGCAAGTACCAACTTGTTGATGACATGGTTGGTTTGACCCTTCACTCTTGTCTATTAGTTCCTTATTTCTCATGGAAAATCAGCCACCGCCGCCACCACTCCAACACCGGTTCCCTCGACCGCAACGAAGTGTTTGTCCCAAAACCAAAATCAAAGGTATCATGGTATAACAAGTACATGAACAATCCACCAGGGAGGGCTATCTCCCTCTTCATCACACTCACACTAGGATGGCCAAT

>61_FAD2_Plate_Plate 01_F05

TTCAAACCCTCCATTCAGTGTTGGCCAACTCAAGAAAGCAATTCCACCACATTGCTTTGAACGTTCTCTTTTCATATCATTCTCCTATGTTGTCTATGATCTCTTAGTGGCCTACTTACTCTTCTACATTGCCACCACTTATTTCCACAAGCTTCCATACCCATTTTCCTTCCTTGCTTGGCCAATCTATTGGGCCATCCAAGGCTGCATTCTCACTGGTGTTTGGGTGATTGCTCATGAGTGTGGCCACCATGCCTTCAGCAAGTACCAACTTGTTGATGACATGGTTGGTTCGACCCTTCACTCTTGTCTATTAGTTCCTTATTTCTCATGGAAAATCAGCCACCGCCGCCACCACTCCAACACCGGTTCCCTCGACCGCAACGAAGTGTTTGTCCCAAAACCAAAATNNAAGGN

>64_FAD2_Plate_Plate 01_F04

TTCAAACCCTCCATTCAGTGTTGGCCAACTCAAGAAAGCAATTCCACCACATTGCTTTGAACGTTCTCTTTTCATATCATTCTCCTATGTTGTCTATGATCTCTTAGTGGCCTACTTACTCTTCTACATTGCCACCACTTATTTCCACAAGCTTCCATACCCATTTTCCTTCCTTGCTTGGCCAATCTATTGGGCCATCCAAGGCTGCATTCTCACTGGTGTTTGGGTGATTGCTCATGAGTGTGGCCACCATGCCTTCAGCAAGTACCAACTTGTTGATGACATGGTTGGTTTGACCCTTCACTCTTGTCTATTAGTTCCTTATTTCTCATGGAAAATCAGCCACCGCCGCCACCACTCCAACACCGGTTCCCTCGACCGCAACGAAGTGTTTGTCCCAAAACCAANATCAAAGGTATCATGGTATAACAAGNNCATGAACNATCCANCAGGGANGG

>68_FAD2_Plate_Plate 01_F08

TTCAAACCCTCCATTCAGTGTTGGCCAACTCAAGAAAGCAATTCCACCACATTGCTTTGAACGTTCTCTTTTCATATCATTCTCCTATGTTGTCTATGATCTCTTAGTGGCCTACTTACTCTTCTACATTGCCACCACTTATTTCCACAAGCTTCCATACCCATTTTCCTTCCTTGCTTGGCCAATCTATTGGGCCATCCAAGGCTGCATTCTCACTGGTGTTTGGGTGATTGCTCATGAGTGTGGCCACCATGCCTTCAGCAAGTACCAACTTGTTGATGACATGGTTGGTTTGACCCTTCACTCTTGTCTATTAGTTCCTTATTTCTCATGGAAAATCAGCCACCGCCGCCACCACTCCAACACCGGTTCCCTCGACCGCAACGAAGTGTTTGTCCCAAAACCAAAATCAAAGGTATCATGGTATAACAAGTACATGAACAATCCACCAGGGAGGGCTATCTCCCTCTTCATCACACTCACACTAGGATGGCCAAT

>69_FAD2_Plate_Plate 01_F09

TTNNAACCCTCCATTCAGTGTTGGCCAACTCAAGAAAGCAATTCCACCACATTGCTTTGAACGTTCTCTTTTCATATCATTCTCCTATGTTGTCTATGATCTCTTAGTGGCCTACTTACTCTTCTACATTGCCACCACTTATTTCCACAAGCTTCCATACCCATTTTCCTTCCTTGCTTGGCCAATCTATTGGGCCATCCAAGGCTGCATTCTCACTGGTGTTTGGGTGATTGCTCATGAGTGTGGCCACCATGCCTTCAGCAAGTACCAACTTGTTGATGACATGGTTGGTTTGACCCTTCACTCTTGTCTATTAGTTCCTTATTTCTCATGGAAAATCAGCCACCGCCGCCACCACTCCAACACCGGTTCCCTCGACCGCAACGAAGTGTTTGTCCCAAAACCAAAATCAAAGGTATCATGGTATAACAAGAACATGATCATTCCACCAGGGAGGGTTATCTCCCTCTTCATCACACTCACTCTAGGATGCNCGAT

>1_FAD2_Plate_Plate 02_A01

TTCNAACCCTCCATTCAGTGTTGGCCAACTCAAGAAAGCAATTCCNCCNNANTGCTTTGAACGTTCTCTTTTCATATCATTCTCCTATGTTGTCTATGATCTCTTAATGGCCTACTTACTCTTCTACATTGCCACCACTTATTTCCACCAGCTTCCATACCCATTTTCCTTCCTTGCTTGGCCAATCTATTGGGCCATCCAAGGCTGCATTCCCACTGGTGTTTGGGTGATTGCTCATGAGTGTGGCCACCATGCCTTCAGCAAGTATCAACTTGTTGATGACATGGTTGGTTTGATCCTTCACTCTTGTCTATTAGTCCCTTATTTCTCATGGAAAATCAGCCACCGCCACCTCCACTCCAGCACCGGTTCCCTCGACCGCGACTAAGTGTTTGTCCTGAAACCAAAATCAAAGGTATCACGGTATAACAAGTACATGAACAATCCACTAGAGAGGGCTATTTCCCTTTTCATCACACTCACACTAGGATGGCCAAT

>4_FAD2_Plate_Plate 02_A04

TTCNAACCCTCCATTCAGTGTTGGCCAACTCAAGAAAGCAATTCCACCACATTGCTTTGAACGTTCTCTTTTCATATCATTCTCCTATGTTGTCTATGATCTCTTAGTGGCCTACTTACTCTTCTACATTGCCACCACTTATTTCCACAAGCTTCCATACCCATTTTCCTTCCTTGCTTGGCCAATCTATTGGGCCATCCAAGGCTGCATTCTCACTGGTGTTTGGGTGATTGCTCATGAGTGTGGCCACCATGCCTTCAGCAAGTACCAACTTGTTGATGACATGGTTGGTTTGACTCTTCACTCTTGTCTATTAGTTCCTTATTTCTCATGGAAAATCAGCCACCGCCGCCACCACTCCAACACCGGTTCCCTCGACCGCAACGAAGTGTTTGTCTCAAAACCAAAATCAAAGGTATCATGGTATAACAAGTACATGAACAATCCACCAGGGAGGGCTATCTCCCTCTTCATCACACTCACACTAGGATGGCCAAT

>16_FAD2_Plate_Plate 02_B04

TTCAANCCCTCCATTCAGTGTTGGCCAACTCAAGAAAGCAATTCCACCACNTTGCTTTGAACGTTCTCTTTTCATATCATTCTCATATGTTGTCTATGATCTCTTAATGGCCTACTTACTCTTCTACATTGCCACCACTTATTTCCACAAGCTTCCATACCCATTTTCCTTCCTTGCTTGGCCAATCTATTGGGCCATCCAAGGCTGCATTCTCACCGGTGTTTGGGTGATTGCTCATGAGTGTGGCCACCATGCCTTCAGCAAGTACCAACTTGTTGATGACATGGTTGGTTTGACCCTTCACTCTTGTCTATTAGTTCCTTATTTCTCATGGAAAATCAGCCACCGCCGCCACCACTCCAACACAGGTTCCCTCAGACCGCGACGAAGTGTTTGTCCCGAAACCAAAATCAAAGGTATCATGGTATAACAAGTACATGAACAATCCACCAGGGAGGGCTATTTCCCTTTTCATCACACTCACACTAGGATGGCCAAT

>32_FAD2_Plate_Plate 02_C08

TTCNAACCCTCCATTCAGTGTTGGCCAACTCAAGAAAGCAATTCCACCACATTGCTTTGAACGTTCTCTTTTCATATCATTCTCCTATGTTGTCTATGATCTCTTAGTGGCCTACTTACTCTTCTACATTGCCACCACTTATTTCCACAAGCTTCCATACCCATTTTCCTTCCTTGCTTGGCCAATCTATTGGGCCATCCAAGGCTGCATTCTCACTGGTGTTTGGGTGATTGCTCATGAGTGTGGCCACCATGCCTTCAGCAAGTACCAACTTGTTGATGACATGGTTGGTTTGACCCTTCACTCTTGTCTATTAGTTCCTTATTTCTCATGGAAAATCAGCCACCGCCGCCACCACTCCAACACCGGTTCCCTCGACCGCAACGAAGTGTTTGTCCCAAAACCAAAATCAAAGGTATCATGGTATAACAAGTACATGAACAATCCACCAGGGAGGGCTATCTCCCTCTTCATCACACTCACACTAGGATGGCCAAT

>37_FAD2_Plate_Plate 02_D01

TTCAANCCCTCCATTCAGTGTTGGCCAACTCAAGAAAGCAATTCCACNNNNTNGCTTTGAACGTTCTCTTTTCATATCATTCTCCTATGTTGTCTGTGATCTCTTAGTGGCCTACTTACTCTTCTACATTGCCACCACTTATTTCCACAAGCTTCCATACCCATTTTCCTTCCTTGCTTGGCCAATCTATTGGGCCATCCAAGGCTGCATTCTCACTGGTGTTTGGGTGATTGCTCATGAGTGTGGCCACCATGCCTTCAGCAAGTACCAACTTGTTGATGACATGGTTGGTTTGACCCTTCACTCTTGTCTATTAGTTCCTTATTTCTCATGGAAAATCAGCCACCGCCGCCACCACTCCAACACCGGTTCCCTCGACCGCAACGAAGTGTTTGTCCCAAAACCAAAATCAAAGGTATCATGGTATAACAAGTACATGAACAATCCACCAGGGAGGGCTATCTCCCTCTTCATCACACTCACACTAGGATGGCCAAT
