# Supplementary material for: Mutagenesis of FAD2 genes in peanut with CRISPR/Cas9 based gene editing
Source: BMC Biotechnol. 2019 Apr 29;19:24. doi: 10.1186/s12896-019-0516-8 (PMC6489235; doi:10.1186/s12896-019-0516-8)
Supplement: Supplementary file 3 — 10 FAD2A gene sequences isolated from hairy root DNAs transformed with gRNA6 in GT-C20. (DOCX 12 kb) [file 12896_2019_516_MOESM3_ESM.docx]

Additional file 3

10 FAD2A gene sequences isolated from hairy root DNAs transformed with gRNA6 in GT-C20.

>2_FAD2A-F sequence exported from 2_FAD2A-F_Plate_Plate 01_A02.ab1

ATGGGAGCTGGAGGGCGTGTCACTAAGATTGAAGCTCAAAAGAAGCCTCTTTCAAGGGTTCCACATTCAAACCCTCCATTCAGTGTTGGCCAACTCAAGAAAGCAATTCCACCACATTGCTTTGAACGTTCTCTTTTCATATCATTCTCCTATGTTGTCTATGATCTCTTAGTGGCCTACTTACTCTTCTACATTGCCACCACTTATTTCCACAAGCTTCCATACCCATTTTCCTTCCTTGCTTGGCCAATCTATTGGGCCATCCAAGGCTGCATTCTCACTGGTGTTTGGGTGATTGCTCATGAGTGTGGCCACCATGCCTTCAGCAAGTACCAACTTGTTGATGACATGGTTGGTTTGACCCTTCACTCTTGTCTATTAGTTCCTTATTTCTCATGGAAAATCAGCCACCGCCGCCACCACTCCAACACCGGTTCCCTCGACCGCAACGAAGTGTTTGTCCCAAAACCAAAATCAAAGGTATCATGGTATAACAAGTACATGAACAATCCACCAGGGAGGGCTATCTCCCTCTTCATCACACTCACACTAGGATGGCCCTTGTACTTGGCCTTCAATGTTTCTGGCAGACCCTATGATAGATTTGCAAGCCACTATGACCCTTATGC

>3_FAD2A-F sequence exported from 3_FAD2A-F_Plate_Plate 01_A03.ab1

ATGGGAGCTGGAGGGCGTGTCACTAAGATTGAAGCTCAAAAGAAGCCTCTTTCAAGGGTTCCACATTCAAACCCTCCATTCAGTGTTGGCCAACTCAAGAAAGCAATTCCACCACATTGCTTTGAACGTTCTCTTTTCATATCATTCTCCTATGTTGTCTATGATCTCTTAGTGGCCTACTTACTCTTCTACATTGCCACCACTTATTTCCACAAGCTTCCATACCCATTTTCCTTCCTTGCTTGGCCAATCTATTGGGCCATCCAAGGCTGCATTCTCACTGGTGTTTGGGTGATTGCTCATGAGTGTGGCCACCATGCCTTCAGCAAGTACCAACTTGTTGATGACATGGTTGGTTTGACCCTTCACTCTTGTCTATTAGTTCCTTATTTCTCATGGAAAATCAGCCACCGCCGCCACCACTCCAACACCGGTTCCCTCGACCGCAACGAAGTGTTTGTCCCAAAACCAAAATCAAAGGTATCATGGTATAACAAGTACATGAACAATCCACCAGGGAGGGCTATCTCCCTCTTCATCACACTCACACTAGGATGGCCCTTGTACTTGGCCTTCAATGTTTCTGGCAGACCCTATGATAGATTTGCAAGCCACTATGACCCTTANGC

>4_FAD2A-F sequence exported from 4_FAD2A-F_Plate_Plate 01_A04.ab1

ATGGGAGCTGGAGGGCGTGTCACTAAGATTGAAGCTCAAAAGAAGCCTCTTTCAAGGGTTCCACATTCAAACCCTCCATTCAGTGTTGGCCAACTCAAGAAAGCAATTCCACCACATTGCTTTGAACGTTCTCTTTTCATATCATTCTCCTATGTTGTCTATGATCTCTTAGTGGCCTACTTACTCTTCTACATTGCCACCACTTATTTCCACAAGCTTCCATACCCATTTTCCTTCCTTGCTTGGCCAATCTATTGGGCCATCCAAGGCTGCATTCTCACTGGTGTTTGGGTGATTGCTCATGAGTGTGGCCACCATGCCTTCAGCAAGTACCAACTTGTTGATGACATGGTTGGTTTGACCCTTCACTCTTGTCTATTAGTTCCTTATTTCTCATGGAAAATCAGCCACCGCCGCCACCACTCCAACACCGGTTCCCTCGACCGCAACGAAGTGTTTGTCCCAAAACCAAAATCAAAGGTATCATGGTATAACAAGTACATGAACAATCCACCAGGGAGGGCTATCTCCCTCTTCATCACACTCACACTAGGATGGCCCTTGTACTTGGCCTTCAATGTTTCTGGCAGACCCTATGATAGATTTGCAAGCCACTATGACCCTTATGC

>5_FAD2A-F sequence exported from 5_FAD2A-F_Plate_Plate 01_A05.ab1

ATGGGAGCTGGAGGGCGTGTCACTAAGATTGAAGCTCAAAAGAAGCCTCTTTCAAGGGTTCCACATTCAAACCCTCCATTCAGTGTTGGCCAACTCAAGAAAGCAATTCCACCACATTGCTTTGAACGTTCTCTTTTCATATCATTCTCCTATGTTGTCTATGATCTCTTAGTGGCCTACTTACTCTTCTACATTGCCACCACTTATTTCCACAAGCTTCCATACCCATTTTCCTTCCTTGCTTGGCCAATCTATTGGGCCATCCAAGGCTGCATTCTCACTGGTGTTTGGGTGATTGCTCATGAGTGTGGCCACCATGCCTTCAGCAAGTACCAACTTGTTGATGACATGGTTGGTTTGACCCTTCACTCTTGTCTATTAGTTCCTTATTTCTCATGGAAAATCAGCCACCGCCGCCACCACTCCAACACCGGTTCCCTCGACCGCAACGAAGTGTTTGTCCCAAAACCAAAATCAAAGGTATCATGGTATAACAAGTACATGAACAATCCACCAGGGAGGGCTATCTCCCTCTTCATCACACTCACACTAGGATGGCCCTTGTACTTGGCCTTCAATGTTTCTGGCAGACCCTATGATAGATTTGCAAGCCACTATGACCCTTATGC

>6_FAD2A-F sequence exported from 6_FAD2A-F_Plate_Plate 01_A06.ab1

ATGGGAGCTGGAGGGCGTGTCACTAAGATTGAAGCTCAAAAGAAGCCTCTTTCAAGGGTTCCACATTCAAACCCTCCATTCAGTGTTGGCCAACTCAAGAAAGCAATTCCACCACATTGCTTTGAACGTTCTCTTTTCATATCATTCTCCTATGTTGTCTATGATCTCTTAGTGGCCTACTTACTCTTCTACATTGCCACCACTTATTTCCACAAGCTTCCATACCCATTTTCCTTCCTTGCTTGGCCAATCTATTGGGCCATCCAAGGCTGCATTCTCACTGGTGTTTGGGTGATTGCTCATGAGTGTGGCCACCATGCCTTCAGCAAGTACCAACTTGTTGATGACATGGTTGGTTTGACCCTTCACTCTTGTCTATTAGTTCCTTATTTCTCATGGAAAATCAGCCACCGCCGCCACCACTCCAACACCGGTTCCCTCGACCGCAACGAAGTGTTTGTCCCAAAACCAAAATCAAAGGTATCATGGTATAACAAGTACATGAACAATCCACCAGGGAGGGCTATCTCCCTCTTCATCACACTCACACTAGGATGGCCCTTGTACTTGGCCTTCAATGTTTCTGGCAGACCCTATGATAGATTTGCAAGCCACTATGACCCTTATGC

>7_FAD2A-F sequence exported from 7_FAD2A-F_Plate_Plate 01_A07.ab1

ATGGGGGCTGGAGGGCGTGTCACTAAGATTAAAGCTCAAAAGAAGCCTCTTTCAAGGGTTCCACATTNAAACCCTCCATTCAGTGTTGGCCAACTCAAGAAAGCAATTCCACCACATTGCTTTGAACGTTCTCTTTTCATATCATTCTCCTATGTTGTCTATGATCTCTTAGTGGCCTACTTACTCTTCTACATTGCCACCACTTATTTCCACAAGCTTCCATACCCATTTTCCTTCCTTGCTTGGCCAATCTATTGGGCCATCCAAGGCTGCATTCTCACTGGTGTTTGGGTGATTGCTCATGAGTGTGGCCACCATGCCTTCAGCAAGTACCAACTTGTTGATGACATGGTTGGTTTGACCCTTCACTCTTGTCTATTAGTTCCTTATTTCTCATGGAAAATCAGCCACCGCCGCCACCACTCCAACACCGGTTCCCTCGACCGCAACGAAGTGTTTGTCCCAAAACCAAAATCAAAGGTATCATGGTATAACAAGTACATGAACAATCCACCAGGGAGGGCTATCTCCCTCTTCATCACACTCACACTAGGATGGCCCTTGTACTTGGCCTTCAATGTTTCTGGCAGACCCTATGATAGATTTGCAAGCCACTATGACCCTTATGC

>8_FAD2A-F sequence exported from 8_FAD2A-F_Plate_Plate 01_A08.ab1

ATGGGAGCTGGAGGGCGTGTCACTAAGATTGAAGCTCAAAAGAAGCCTCTTTCAAGGGTTCCACATTCAAACCCTCCATTCAGTGTTGGCCAACTCAAGAAAGCAATTCCACCACATTGCTTTGAACGTTCTCTTTTCATATCATTCTCCTATGTTGTCTATGATCTCTTAGTGGCCTACTTACTCTTCTACATTGCCACCACTTATTTCCACAAGCTTCCATACCCATTTTCCTTCCTTGCTTGGCCAATCTATTGGGCCATCCAAGGCTGCATTCTCACTGGTGTTTGGGTGATTGCTCATGAGTGTGGCCACCATGCCTTCAGCAAGTACCAACTTGTTGATGACATGGTTGGTTTGACCCTTCACTCTTGTCTATTAGTTCCTTATTTCTCATGGAAAATCAGCCACCGCCGCCACCACTCCAACACCGGTTCCCTCGACCGCAACGAAGTGTTTGTCCCAAAACCAAAATCAAAGGTATCATGGTATAACAAGTACATGAACAATCCACCAGGGAGGGCTATCTCCCTCTTCATCACACTCACACTAGGATGGCCCTTGTACTTGGCCTTCAATGTTTCTGGCAGACCCTATGATAGATTTGCAAGCCACTATGACCCTTATGC

>9_FAD2A-F sequence exported from 9_FAD2A-F_Plate_Plate 01_A09.ab1

ATGGGAGCTGGAGGGCGTGTCACTAAGATTGAAGCTCAAAAGAAGCCTCTTTCAAGGGTTCCACATTCAAACCCTCCATTCAGTGTTGGCCAACTCAAGAAAGCAATTCCACCACATTGCTTTGAACGTTCTCTTTTCATATCATTCTCCTATGTTGTCTATGATCTCTTAGTGGCCTACTTACTCTTCTACATTGCCACCACTTATTTCCACAAGCTTCCATACCCATTTTCCTTCCTTGCTTGGCCAATCTATTGGGCCATCCAAGGCTGCATTCTCACTGGTGTTTGGGTGATTGCTCATGAGTGTGGCCACCATGCCTTCAGCAAGTACCAACTTGTTGATGACATGGTTGGTTTGACCCTTCACTCTTGTCTATTAGTTCCTTATTTCTCATGGAAAATCAGCCACCGCCGCCACCACTCCAACACCGGTTCCCTCGACCGCAACGAAGTGTTTGTCCCAAAACCAAAATCAAAGGTATCATGGTATAACAAGTACATGAACAATCCACCAGGGAGGGCTATCTCCCTCTTCATCACACTCACACTAGGATGGCCCTTGTACTTGGCCTTCAATGTTTCTGGCAGACCCTATGATAGATTTGCAAGCCACTATGACCCTTATGC

>10_FAD2A-F sequence exported from 10_FAD2A-F_Plate_Plate 01_A10.ab1

ATGGGAGCTGGAGGGCGTGTCACTAAGATTGAAGCTCAAAAGAAGCCTCTTTCAAGGGTTCCACATTCAAACCCTCCATTCAGTGTTGGCCAACTCAAGAAAGCAATTCCACCACATTGCTTTGAACGTTCTCTTTTCATATCATTCTCCTATGTTGTCTATGATCTCTTAGTGGCCTACTTACTCTTCTACATTGCCACCACTTATTTCCACAAGCTTCCATACCCATTTTCCTTCCTTGCTTGGCCAATCTATTGGGCCATCCAAGGCTGCATTCTCACTGGTGTTTGGGTGATTGCTCATGAGTGTGGCCACCATGCCTTCAGCAAGTACCAACTTGTTGATGACATGGTTGGTTTGACCCTTCACTCTTGTCTATTAGTTCCTTATTTCTCATGGAAAATCAGCCACCGCCGCCACCACTCCAACACCGGTTCCCTCGACCGCAACGAAGTGTTTGTCCCAAAACCAAAATCAAAGGTATCATGGTATAACAAGTACATGAACAATCCACCAGGGAGGGCTATCTCCCTCTTCATCACACTCACACTAGGATGGCCCTTGTACTTGGCCTTCAATGTTTCTGGCAGACCCTATGATAGATTTGCAAGCCACTATGACCCTTATGC

>11_FAD2A-F sequence exported from 11_FAD2A-F_Plate_Plate 01_A11.ab1

ATGGGAGCTGGAGGGCGTGTCACTAAGATTGAAGCTCAAAAGAAGCCTCTTTCAAGGGTTCCACATTCAAACCCTCCATTCAGTGTTGGCCAACTCAAGAAAGCAATTCCACCACATTGCTTTGAACGTTCTCTTTTCATATCATTCTCCTATGTTGTCTATGATCTCTTAGTGGCCTACTTACTCTTCTACATTGCCACCACTTATTTCCACAAGCTTCCATACCCATTTTCCTTCCTTGCTTGGCCAATCTATTGGNCCATCCAAGGCTGCATTCTNNCTGGTGTTTGGGTGATTGCTCATGAGTGTGGCCNCCATGCCTTCAGCAAGTACCAACTTGTTGATGACATGGTTGGTTTGACCCTTCACTCTTGTCTATTAGTTCCTTATTTCTCATGGAAAATCACCCACCGCCGCCGCCACTCCAACACCGGTTCCCTCGACCGCAACGAAGTGTTTGTCCCAAAACCAAAATCAAAGGTATCATGGTATAACAAGTACATGAACAATCCACCAGGGAGGGCTATCTCCCTCTTCATCACACTCACACTAGGATGGCCCTTGTCCTTGGCCTTCAATGTTTCTGGCAGACCCTATGATAGATTTGCAAGCCACTATGACCCTTATGC
